# *Staphylococcus arlettae* Genomics: Novel Insights on Candidate Antibiotic Resistance and Virulence Genes in an Emerging Opportunistic Pathogen

**DOI:** 10.3390/microorganisms7110580

**Published:** 2019-11-19

**Authors:** Anna Lavecchia, Matteo Chiara, Caterina De Virgilio, Caterina Manzari, Rosa Monno, Armando De Carlo, Carlo Pazzani, David Horner, Graziano Pesole, Antonio Placido

**Affiliations:** 1 Department of Biosciences, Biotechnology and Biopharmaceutics, University of Bari “Aldo Moro”, 70126 Bari, Italy; anna.lavecchia5@gmail.com (A.L.); caterina.devirgilio@uniba.it (C.D.V.); graziano.pesole@uniba.it (G.P.); 2 Department of Biosciences, University of Milan, 20133 Milan, Italy; matteo.chiara@unimi.it (M.C.); david.horner@unimi.it (D.H.); 3 Institute of Biomembranes, Bioenergetics and Molecular Biotechnologies, National Research Council, 70126 Bari, Italy; c.manzari@ibiom.cnr.it; 4 Department of Basic Medical Sciences, Neurosciences and Sense Organs, University of Bari Aldo Moro, 70124 Bari, Italy; rosa.monno@uniba.it; 5 U.O.C. Hospital-University Analysis Laboratory-U.O.S.D. Microbiology and Virology-OO. RR., 71122 Foggia, Italy; armdeca@gmail.com; 6 Department of Biology, University of Bari Aldo Moro, 70126 Bari, Italy; carlo.pazzani@uniba.it

**Keywords:** genomics, NGS sequencing, draft genome, coagulase negative staphylococci, *Staphylococcus arlettae*, antibiotic resistance, virulence

## Abstract

Coagulase Negative Staphylococci (CoNS) are becoming increasingly recognized as an important cause of human and animal infections. Notwithstanding their clinical relevance, annotation of genes potentially involved in pathogenicity and/or antibiotic resistance in the CoNS species *Staphylococcus arlettae* (SAR) is currently very limited. In the current work we describe the genome of a novel methicillin resistant isolate of SAR, which we named Bari, and present a comprehensive analysis of predicted antibiotic resistance profiles and virulence determinants for all the 22 currently available SAR genomes. By comparing predicted antibiotic resistance and virulence-associated genes with those obtained from a manual selection of 148 bacterial strains belonging to 14 different species of staphylococci and to two “outgroup” species, *Bacillus subtilis* (BS) and *Macrococcus caseoliticus* (MC), we derived some interesting observations concerning the types and number of antibiotic resistance-related and virulence-like genes in SAR. Interestingly, almost 50% of the putative antibiotic resistance determinants identified in this work, which include the clinically relevant *mec*, *van,* and *cls* genes, were shared among all the SAR strains herein considered (Bari included). Moreover, comparison of predicted antibiotic resistance profiles suggest that SAR is closely related to well-known pathogenic *Staphylococcus* species, such as *Staphylococcus aureus* (SA) and *Staphylococcus epidermidis* (SE). A similar analysis of predicted virulence factors, revealed that several genes associated with pathogenesis (including, for example, *ica*, *nuc*, and *ssp*), which are commonly found in the genomes of pathogenic staphylococci such as *Staphylococcus haemolyticus* (SH) and *Staphylococcus saprophyticus* (SS), are observed also in the SAR strains for which a genomic sequence is available. All in all, we believe that the analyses presented in the current study, by providing a consistent and comprehensive annotation of virulence and antibiotic resistance-related genes in SAR, can constitute a valuable resource for the study of molecular mechanisms of opportunistic pathogenicity in this species.

## 1. Introduction

The first strains of *Staphylococcus arlettae* (SAR) were reported in 1984, isolated from the skin and nares of poultry and goats [1]. Since then, SAR strains have been isolated from different animals (mainly mammals and birds) and environments including salt mines, estuaries, fermented foods, and biological safety cabinets [2,3,4,5,6]. Although SAR is normally considered a commensal species, it may also be associated to different types of infections or in contexts where a large use of antibiotics is applied. For example, SAR strains were isolated from bovine mastitis, pig exudative epidermidis, dairy goat intramammary infection, a human patient affected by rheumatic mitral stenosis, as well as from blood clinical samples [7,8,9]. Recently a plasmid encoding for nine antibiotic resistance genes, *cfr*, *erm*(C), *tet*(L), *erm*(T), *aadD*, *fosD*, *fexB*, *aacA-aphD*, and *erm*(B), was characterized in SA-01, a SAR strain isolated from a chicken farm. The plasmid contained three IS431 elements mediating intra- or inter-plasmid recombination, and was considered as a potential vector of antibiotic resistance genes with relevant implications on the effectiveness of clinical therapy based on antimicrobials [10]. More recently, an operon encoding for a novel functional *β*-lactamase (bla_ARL_) was detected in SAN1670, a SAR strain isolated from bovine mastitis. Interestingly, bla_ARL_ was located in a high-mobility genomic island, suggesting its potential for mobilization and lateral gene transfer [11]. Independent studies have also identified several multidrug efflux pumps (e.g., *norA*) coding genes as well as other genes related to resistance to antibiotics such as chloramphenicol (e.g., *fexA*), tetracycline (e.g., *tetL*), and erythromycin (e.g., *msrA*, *mphC*) in the genomes of SAR strains isolated from chicken farm and dairy herds affected by mastitis [10,12,13]. The fosfomycin resistant *fos*D gene was described in a novel plasmid of the SAR SA-01 strain [10]. Since *fos*D-resistance genes are typically located in mobile genetic elements, they may contribute to multi-resistant traits to other staphylococci [14,15,16].

Additionally, several virulence-associated genes, including fibronectin/fibrinogen binding protein, programmed cell death toxin *ydc*D, hemolysin III, autolysins (*atl*), and genes involved in the regulation of virulence accessory factors such as *agr*A, *agr*B, *agr*R, *agr*V, and *agr*Z, were identified in the SAR CVD059 strain isolated from the blood of a cardiovascular disease patient [8,17,18].

Although SAR is emerging as an important opportunistic pathogen, apart from the studies discussed above, to date there is no comprehensive information on antibiotic resistance and presence/absence of virulence-associated genes for most of the 22 SAR isolates from which genomic sequences are available.

The resistance to most *β*-lactam antibiotics is a typical trait of many pathogenic staphylococci, collectively defined as methicillin-resistant staphylococci, and currently represents a relevant problem in clinical treatment of *Staphylococcus* infections. Indeed, methicillin resistance is usually associated with resistance to additional antibacterial agents, producing a multi-resistant phenotype that may further compromise the therapy [19]. However, at present, information concerning the presence/absence of *β*-lactam resistance genes is currently lacking for several Coagulase Negative Staphylococci (CoNS) species including SAR.

In this study, we present the draft genome of a novel SAR methicillin resistant strain, which we named Bari, isolated from a disused biological safety cabinet, and perform systematic bioinformatics predictions of antibiotic resistance and virulence-associated genes for all 22 SAR genomes available in the NCBI database. By comparing the predicted profiles with equivalent profiles obtained from a manually curated selection of 148 *Staphylococcus* genomes belonging to fourteen species and two distantly related groups of Firmicutes, *Bacillus subtilis* (BS) and *Macrococcus caseolyticus* (MC), we highlight for all the SAR genomes considered, the presence of several antibiotic-resistance genes and virulence determinants never reported thus far, which are commonly detected in pathogenic staphylococci.

The analyses presented in the current study, by providing a consistent and comprehensive annotation of virulence and antibiotic resistance-related genes in SAR, can constitute a valuable resource for the study of molecular mechanisms of opportunistic pathogenicity in this species.

## 2. Materials and Methods

### 2.1. Isolation and Sequencing of SAR Bari

The SAR Bari strain was isolated from a Nunc bioassay plate, containing LB (Lauria Bertani) agar supplemented with hexavalent chromate Cr (VI), after an incubation at room temperature on the top of a disused biological safety cabinet. SAR Bari exhibited a high resistance to Cr (VI), up to 150 mM in LB and M9 minimal broth. Genomic DNA was extracted with the DNeasy blood and tissue kit (Qiagen, Hilden, Germany) and sequenced by an Illumina MiSeq instrument (San Diego, CA, USA) by producing 2 × 250 nucleotide bp paired-end reads.

### 2.2. Genome Assembly and Taxonomic Classification of SAR Bari

Raw sequencing data from the Illumina platform were processed using a modified version of the “Fosmid1” pipeline in the A-GAME Galaxy framework [20,21]. Quality trimming was executed using the sliding-window operation in Trimmomatic with default parameters [22]. Overlapping reads were merged using PEAR with standard parameters [23]. The final assembly was performed using the SPAdes assembler (version 3.50) using kmers of 33, 55, 77, 99, and 121 nt [24]. Annotation was performed with PROKKA with default parameters [25]. The draft genome of SAR Bari was deposited in NCBI under the accession number WEIN00000000, BioSample number SAMN12991358 and BioProject number ID PRJNA576354. Taxonomic classification of SAR Bari was performed by using Tetra Correlation Search (TCS) and ANI (Average Nucleotide Identity), as implemented by the JspeciesWS web server http://jspecies.ribohost.com/jspeciesws/) [26].

### 2.3. Antimicrobial Susceptibility Testing

Antimicrobial susceptibility of the SAR Bari strain was determined by a BD PHOENIX^™^ 100 instrument (Becton Dickinson, Franklyn Lake, NJ). Data were elaborated by the BD Epicenter Expert System according to EUCAST rules (http://www.eucast.org). The PMIC/ID-88 (BD) panel was used to test susceptibility to ampicillin, cefoxitin, ceftaroline, ciprofloxacin, clindamycin, daptomycin, erythro-mycin, fosfomycin, fusidic acid, gentamicin, imipenem, linezolid, moxifloxacin, mupirocin, nitro-furantoin, oxacillin, penicillin, rifampin, teicoplanin, tetracyclin, tigecycline, trimethoprim/ sulfamethoxazole, and vancomycin. The Epsilometer Test (ETest) was used for testing resistance to ciprofloxacin, daptomycin, erythromycin, gentamicin, moxifloxacin, tetracyclin, tigecycline, trimethoprim/sulfamethoxazole, and vancomycin (bioMérieux, Marcy-L’Étolie, France and Liofilchem, Roseto degli Abruzzi, Italy). All tests were repeated on four independent technical replicates. MIC interpretative breakpoints were defined according to EUCAST recommendations. *Staphylococcus aureus* ATCC 29213 was used as a control strain.

### 2.4. Comparative Genomics Dataset

In order to carry out a comparative analysis of predicted profiles of antibiotic resistance and virulence-associated genes in the genus *Staphylococcus*, 148 strains from 14 different species of *Staphylococcus* (SAR included) were selected, including two distantly related species (BS and MC) to provide an outgroup for comparative analyses.

*Staphylococcus* species included in these analyses were selected based on the criteria previously proposed [27]. Briefly, the *Staphylococcus* genus was subdivided in six major sub-clades. For each sub-clade one or more representative species were selected based on the availability of genomic sequences and annotation of protein coding genes in GenBank. Only species for which genome annotation for at least four distinct strains and for which the genome sequences of the representative types of strains were available were included in the study. A total of 148 strains belonging to 16 species were selected based on these criteria. These include: 14 *Staphylococcus arlettae* (SAR), 13 *Staphylococcus aureus* (SA), 12 *Staphylococcus saprophyticus* (SS), 12 *Staphylococcus cohnii* (SC), 11 *Staphylococcus epidermidis* (SE), 11 *Staphylococcus haemolyticus* (SH), 11 *Staphylococcus simulans* (SSI), 11 *Staphylococcus sciuri* (SSC), 5 *Staphylococcus kloosii* (SK) 5 *Staphylococcus hyicus* (SHY), 5 *Staphylococcus chromogenes* (SCH), 5 *Staphylococcus agnetis* (SAG), 5 *Staphylococcus felis* (SF), 4 *Staphylococcus auricularis* (SAU), 13 *Bacillus subtilis* (BS), and 11 *Macrococcus caseolyticus* (MC). The complete list of SAR strains used in this study is reported in Table S1. All genomes deposited before June 2019 were considered. The complete list of species included in the dataset for the comparative genomics analysis, with the corresponding GenBank accession numbers, are in Table S2.

### 2.5. Genomics of Antibiotic Resistance

Putative antibiotic resistance genes were detected by RGI v.5.1.0 (Resistance Gene Identifier, https://card.mcmaster.ca/analyze/rgi) using the CARD (Comprehensive Antibiotic Resistance Database, https://card.mcmaster.ca/home) reference collection on the predicted annotated CDS (CoDing Sequence) for all considered species [28]. All the analyses were performed using the RGI web portal with default parameters. The RGI output was imported in Cytoscape v.3.7.2. to generate a network of shared antibiotic resistance determinants, where the distance among species was proportional to the number of unique elements detected per species and connections among species [29].

### 2.6. Genomics of Virulence

Prediction of virulence-associated genes was performed by VRprofile v.2.0 (http://db-mml.sjtu.edu.cn/STEP/index.php) and VFanalyzer (http://www.mgc.ac.cn/cgi-bin/VFs/v5/main.cgi?func=VFanalyzer) tools, using default parameters [30,31]. VRprofile can predict 15 different types of virulence-related elements, including Virulence Factors (VF), Acquired Antibiotic Resistance Determinants (AR), Type III secretion effectors (T3SE), Type IV secretion effectors (T4SE), Type VI secretion effectors (T6SE), Type VII secretion effectors (T7SE), Type III secretion systems (T3SS), Type VI secretion systems (T6SS), Type VII secretion systems (T7SS), Prophage, Class I Integrons, Insertion Sequence elements (IS), Pathogenicity Islands (PAI), Antibiotic Resistance Islands (ARI), and Integrative and Conjugative Elements (ICE). VFanalyzer was instead applied for the prediction of specific virulence factors (e.g., enzymes, toxins, capsule). Clustering of SAR and other species investigated based on the number of virulence-related genes per species was performed by applying PermutMatrix (version 1.9.3) [32]. The comparison of virulence elements and factors among species were carried out by the Draw Venn Diagram web resource (http://bioinformatics.psb.ugent.be/webtools/Venn).

## 3. Results

### 3.1. Genomics and Taxonomy of SAR (Staphylococcus arlettae) Bari

The final assembly of the SAR Bari strain consisted of 75 scaffolds, for a total size of 2,547,240 bp and an N50 of 90.3 Kb. A total of 2530 genes and 2460 coding sequences (CDS) were annotated. Taxonomic characterization based on TCS suggested that SAR Bari was very closely related to the SAR type strain (NCTC 12413^T^), with a z-score of 0.99915, which is marginally above the cut-off value for species identification. Average Nucleotide Identity (ANI) between the draft genome assembly of SAR Bari and NCTC 12413^T^ was 98.68%, that is well above the value typically used for species delineation (95–96%). Based on this evidence, both TCS and ANI suggested the classification of the isolate as a novel SAR strain, which was then named *Staphylococcus arlettae* Bari.

### 3.2. Antibiotic Resistance of SAR Bari

The experimental results of the antimicrobial susceptibility test showed that SAR Bari was resistant to oxacillin and to all *β*-lactam antibiotics tested (ampicillin, cefoxitin, ceftaroline, imipenem, penicillin). Resistance to clindamycin, erythromycin, fosfomycin, and fusidic acid was also observed. SAR Bari was sensitive to ciprofloxacin, moxifloxacin, daptomycin, teicoplanin, vancomycin, tetracycline, tigecycline, gentamicin, linezolid, trimethoprim/sulfamethoxazole, mupirocin, nitrofurantoin, and rifampin. A full concordance between results obtained by the BD Phoenix and ETest was observed.

Experimental data were in line with the prediction carried out by the RGI software, which identified a total of 227 potential antibiotic resistance genes in the SAR Bari potential proteome, showing significant sequence similarity with protein sequences included in the CARD database (Table 1).

The majority of these genes were annotated as multidrug resistance proteins, however, proteins likely involved in the resistance to specific antibiotic classes such as aminoglycosides (e.g., *aac*(6′)-*iw*, *aac*(6′)-*ie*-*aph*(2″)-*ia*, *spd*), fluoroquinolones (e.g., *gyr*A, *eme*A), fosfomycine (e.g., *mur*A, *fos*D, *fos*A6), glycopeptides (e.g., *van*E, *van*L), and tetracycline (e.g., *tet*T, *otr*A) were also observed.

**Figure 1:**
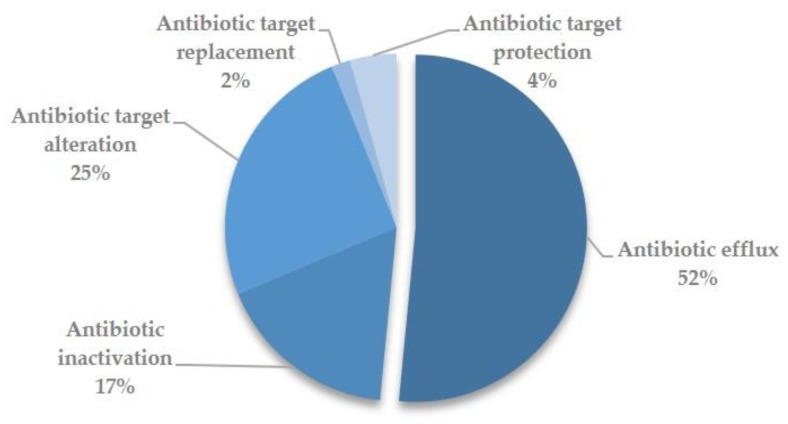
Pie chart of molecular determinants involved in antibiotic resistance mechanisms of SAR Bari draft genome.

The majority of candidate antibiotic resistance proteins identified by RGI were associated with efflux-based mechanisms of antibiotic resistance (52%), with other hits correlated to resistance mechanisms involving antibiotic target alteration, protection and replacement (31%), and antibiotic inactivation (17%) (Figure 1).

### 3.3. Antibiotic Resistance Genomics of SAR and Species Dataset

The number of putative antibiotic resistance genes identified by RGI in other SAR strains was substantially equivalent to that identified for SAR Bari (Table 2), ranging from 234 (AR15) to 212 (AR12). Consistent with our previous observations, 112–125 of these proteins were associated with efflux-based mechanisms of antibiotic resistance (Table 2).

Comparative analysis between resistomes of SAR Bari and all other SAR strains evidenced 94 shared antibiotic determinants, whereas eleven strains (SAR Bari included) were characterized by unique antibiotic determinants (Table S3). However, we noticed that the numbers of these unique determinants were consistently low (≤3) for most of the strains, with *mex*L; *aac*(6′)-34; *arl*-1; *aac*(6′)-*ie*-*aph*(2″)-*ia* exclusively found in SAR Bari.

The RGI analysis of antibiotic resistance determinants in the extended dataset of 148 strains detected a total of 505 distinct antibiotic resistance-like genes (Table S4) with thirty-six shared between all species investigated. Unique determinants were identified for all 16 species of the dataset. While nine genes were found to be specific to SAR isolates (*cat*A8, *fos*B3, *emr*A, *gim*A, *spd*, tetA, *fos*A6, *mdt*H, and *otr*C genes), a number of SAR genes were shared exclusively with SA (*imp*-35 gene), BS (*van*SN, *qep*A4, *cau*-1, *cat*B9 genes), and SK (*sox*S, *qac*B genes).

**Figure 2:**
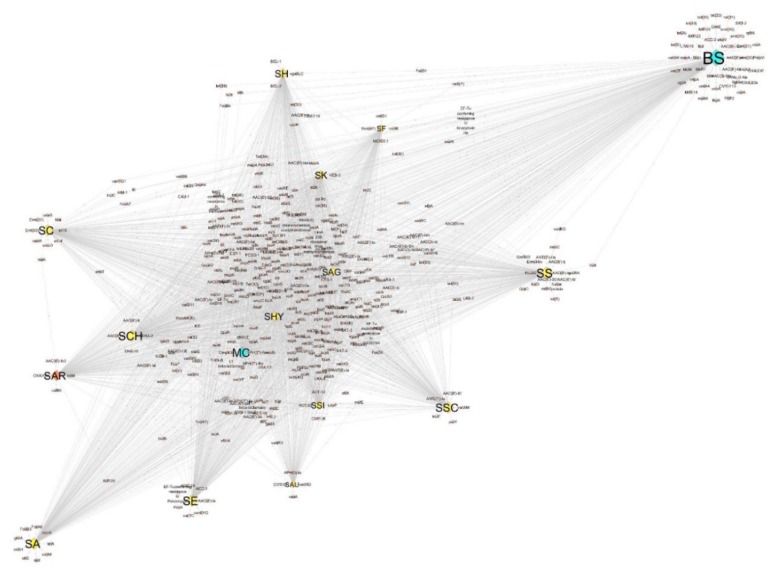
Network of antibiotic resistance determinants shared between all investigated species. Network was built by Cytoscape v. 3.7.2. The distance between species is proportional to the number of unique elements per species and connections among species. Orange diamond, SAR; yellow diamonds, *Staphylococcus* species (SA, SE, SAU, SSI, SSC, SCH, SC, SHY, SS, SAG, SK, SH, SF); light blue diamonds, outgroup species (BS, MC). The size of each diamond corresponds to the number of antibiotic resistance determinants per species. See Table S4 for details on the molecular elements of antibiotic resistance shared among the species in the dataset. Species abbreviations are in the Material and Methods Section (Section 2.4).

Similarity of global profiles of predicted antibiotic resistance patterns are represented in the form of a network graph in Figure 2, where nodes represent species and edges represent shared predicted antibiotic resistance genes. As outlined in the figure, predicted resistome genes identified in BS are remarkably different from predicted resistome genes of other staphylococci and MC. Reduced levels of diversity are observed for SH, SS, SSC, SAU, SE, and SC, with SAR more closely related to SA, SE, SC, and SCH.

### 3.4. Analysis of the Distribution of Putative Virulence Elements

Prediction of virulence-associated genes in SAR Bari, carried out by VRprofile, identified a total of 440 putative virulence-related genes, showing significant levels of similarity with VF, AR, T3SE, T4SE, T6SE, T7SE, T3SS, T6SS, T7S, Prophage, Integrons, IS, PAI, and ARI virulence elements in the VFDB database (Table S5). The majority of the genes identified by the VRprofile were annotated as VF (135), ARI (66), PAI (84), Prophage (111), and AR (120), while T3SE, T4SE, T3SS, T6SS, Integrons, and IS were much less represented.

Comparative analysis did not evidence relevant differences between the virulence profile of SAR Bari and all other SAR strains (Table S6) with, for example, AR2 (657) and TSAR (644) showing a higher number of virulence elements identified with respect to other strains such as AR4 (521) or B (Bari, 542).

**Figure 3:**
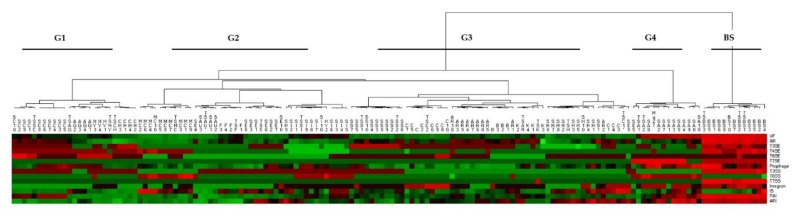
Heatmap of virulence elements detected in the dataset. Clustering was based on comparative quantitative analysis of similar virulence elements identified within 148 genomes of the dataset. The different groups represent five different sets of virulence profiles. Color scale corresponds to the number of predicted genes associated to specific virulence element, from green (lowest) to red (highest). Note: Abbreviations used are relative to single strains (see Table S2 for details).

Clustering of virulence elements profiles performed on the 148 genomes dataset, which are represented in the heatmap in Figure 3 (data in Table S6), highlighted two major groups, one of them possibly split into four additional subgroups (G1–G4) and the other corresponding to BS. G1 and G2 include virulence profiles of SSC, SAG, SHY, SCH, SAU, SSI, SE, SH, and MC strains. G3 includes virulence profiles of SAR strains as well as other species (SC, SK, SH, and SS). In particular, SAR Bari (B) showed a high similarity with SAR B1, B2, and B3. G3 also included the SE strain (SE9). G4, well separated from the other groups, was instead represented by virulence profiles of SA strains.

### 3.5. Virulence Factors within SAR and Species Dataset

VFanalyzer identified a total of 23 putative virulence factors, related to five different classes (VFclass) in the genome of SAR Bari (Table 3). Most of these (14) were related to genes (capsule undetermined, *cap*B, *cap*C, *gal*E) coding for virulence factors involved in immune system evasion (VFclass 2). Additional predicted virulence factors were associated with allantoine utilization (4 hits, nutritional factors); lipase (1 hit, *lip*), protease (1 hit, *ssp*A), and nuclease activity (1 hit, *nuc*); virulence-factor-related to the process of antiphagocytosis (1 hit, *uge*); and serum resistance and immune evasion (1 hit, *wbtP*).

All the predicted virulence factors identified in SAR Bari were present in at least one of the SAR strains included in the study (Table 4).

In total, 39 distinct virulence-related genes were identified by VFanalyzer in SAR. Of these, only two genes, one related to VFclass-immune evasion (capsule undetermined) and one to the VFclass-enzyme (*ssp*A gene related to a serine protease), were shared between all SAR strains. The *gro*EL and *lpl*A1 genes were found exclusively in the AR8 strain, while *hem*L and *ica*C genes were identified solely in the AR12 and AR17 strains.

Profiles of presence/absence of virulence-factor-related genes identified within SAR strains were compared with equivalent profiles derived from the analyses of the other 15 bacterial species considered in this study. The complete results of these analyses are reported in Table S7. Comparison of the presence/absence of virulence-related genes identified in SAR with the other species considered in the study are reported in Table 5. A total of 160 distinct virulence-factor-related genes were identified in the 148 genomes included in our dataset, of these, 37 were shared between SAR and different species in the dataset (Table 5). Among these, SAR shared four genes uniquely with BS (*plr*/*gap*A, *kat*A, *acp*XL, and *gnd*), one gene with SAU (*lpe*A), and one gene with SE (*eno*). Further, SAR had six unique genes (*flm*H, *T6SS*-II, *tuf*, *gro*EL, *slr*A, and *sig*A/*rpo*V).

## 4. Discussion

Despite the considerable clinical and environmental relevance of CoNS and the availability of an increasing number of genomic sequences, at present, information concerning SAR, particularly the presence/absence of putative virulence factors and antibiotic resistance genes, is limited.

In this study we present a detailed comparative genomics analysis of predicted resistomes and virulence factors of a carefully selected collection of 124 representative staphylococci, including all the currently available SAR genomes.

Our analyses highlight the presence of numerous candidate antibiotic resistance genes in SAR. Interestingly, several of these genes are commonly observed in other *Staphylococcus* species, suggesting a widespread presence of putative antibiotic resistance genes in the genomes of staphylococci. On the other hand, some genes (e.g., *fos*B3 and *tet*A) are consistently observed only in SAR isolates, or are shared between SAR and a limited number of commensal (*sox*S with SK) and pathogenic species (e.g., *imp-*35 and *cat*B9 with SA and BS, respectively).

The global analysis of predicted antibiotic resistance profiles, as shown in Figure 2, clearly indicated that the predicted resistome of SAR is closely related to the predicted resistomes of pathogenic species, including SE and SA.

Notably, we observe that the predicted antibiotic resistance profile of MC, which was included in our analyses to serve as a reasonably phylogenetically distant outgroup, was very closely related to that of several *Staphylococcus* species, suggesting that these bacteria have very similar antibiotic resistance profiles. This observation might reflect that *Macrococcus* and *Staphylococcus* can be found associated in different environments (e.g., animal infections, milk, meat products) were horizontal gene transfer could take place [33].

However, the observation that *Macrococcus* and *Staphylococcus* are very closely related from a phylogenetic point of view could provide an equally likely explanation [34]. Importantly, predicted antibiotic resistance profiles recovered for BS, which in this study was used as an alternative outgroup species, were considerably different from those of staphylococcal genomes.

All the SAR strains herein considered contained a similar number of predicted antibiotic resistance genes. Unsurprisingly these genes were consistently annotated to similar functional classes. The majority (~50%) of putative antibiotic resistance genes identified in SAR strains were consistently associated with the efflux-based mechanism of resistance (Table 2). In this respect, it should be noted that several recent studies suggest an increasing relevance of efflux pumps in antibiotic resistance mechanisms [35]. Efflux pumps displayed a tendency to promote loss of substrate specificity, which could translate into multi-resistance [36,37]. Moreover, as evidenced in a recent large-scale genomics investigation of pathogenicity determinants, many efflux pumps do not exclusively export antimicrobials, but could be implicated in bacterial virulence, representing potent targets for adaptation to a pathogenic lifestyle [38,39].

Our genomic analysis also predicted some genes of clinical relevance for all SAR strains. These include methicillin resistance (*mec*D, *mec*R1, and *mec*I), vancomycin resistance (e.g., *van*KI, *van*HA, *van*TG, and *van*YM) as well as daptomycin resistance (*cls*, *rpo*B, and *pgs*A). Moreover, the SAR Bari strain, which was described and characterized for the first time in this study, contained a putative *arl-1* gene encoding for a novel β-lactamase, which was recently identified by whole-genome sequencing of a penicillin-resistant SAR strain (SAN1670) [11]. Interestingly, this gene was observed only in the SAN1670 and Bari strains, while all the other SAR strains and *Staphylococcus* species considered in this study contained the *blaZ* gene, coding for the most common β-lactamase found in staphylococci.

Notwithstanding these very interesting considerations, we observe that the predicted profile of antibiotic resistance of the SAR Bari strain does not completely match the experimental data obtained in this study. Indeed, while we observe a perfect correspondence between candidate resistance genes and in vitro resistance to several antibiotics (e.g., all used β-lactam antibiotics, fosfomycin, fusidic acid, erythromycin), it is also true that SAR Bari was sensitive to some antimicrobials (e.g., tetracycline, gentamicin, teicoplanin) for which putative resistance genes were predicted. These observations could be explained by several possible reasons, including complex regulatory mechanisms of gene expression, reduced enzymatic activity of some predicted candidate genes, or involvement of these genes in the resistance to other types of antimicrobials. Notwithstanding these limitations, we believe that the availability of a large and comprehensive annotation of candidate antimicrobial resistance genes in CoNS will constitute an important resource for the functional characterization of these genes and a more detailed understanding of the molecular mechanisms involved in antibiotic resistance.

Bioinformatics prediction of virulence-related genes identified a consistent number of putative virulence elements and factors (Table 5 and Table S6) in the genomes of all the SAR strains. Comparative analyses suggested that the predicted virulence profiles of SAR isolates have a relevant similarity with other pathogenic staphylococci, including SS and SH (Figure 3, Table S6).

These observations are confirmed even when a specific set of virulence factors (e.g., *ssp*, *nuc*, *cap*) involved in immune evasion is considered.

Remarkably, the putative orthologs of the *ssp*, *nuc,* and *cap*, which have recently been described in the majority of opportunistic CoNS pathogenic species (SE, SH, and SS), were observed in all SAR strains for which genomic sequences are available [40].

The presence of a high number of predicted antibiotic resistance genes and virulence-related elements in SAR suggests that these genes might constitute an important genetic reservoir of genes of clinical relevance for other pathogenic bacterial strains and species.

In conclusion, we believe that by providing, for the first time, an extensive annotation of potentially clinically relevant pathogenic genes in SAR isolates, the analyses here presented can constitute a valuable resource for the study of molecular mechanisms of opportunistic pathogenicity in this species and the functional characterization of antibiotic resistance and virulence genes of CoNS in general.

## Figures and Tables

**Table 1:** In vitro antibiotic susceptibility of *Staphylococcus arlettae* (SAR) Bari and correlated predicted genes.

	Drug Class	Antibiotic Tested	In Vitro Susceptibility	Predicted Genes
1.	*β*-Lactam	Oxacillin	R	*amp*S, *amp*C, *gob*-2, *gob*-18, *imp*-35, *arl*-1, *nmc*A, *nmc*R, *mec*D, *mec*I, *mec*R1
2.		Ampicillin	R	
3.		Cefoxitin	R	
4.		Ceftaroline	R	
5.		Imipenem	R	
6.		Penicillin	R	
7.	Lincosamide	Clindamycin	R	*erm*C, *erm*K, *cfr*C, *ole*C, *mex*L, *mex*S
8.	Macrolide	Erythromycin	R	
9.	-	Fosfomycin	R	*fos*D, *fos*B5, *fos*A6, *fos*B2
10.	Fusidic Acid	Fusidic Acid	R	*fus*A
11.	Fluoroquinolone	Ciprofloxacin	S	*gyr*A, *gyr*B, *nor*A, *nor*B, *pmr*A, *pat*A, *arl*S, *arl*R
12.		Moxifloxacin	S	
13.	Peptide	Daptomycin	S	*cls*, *pgs*A, *mpr*f
14.	Glycopeptide	Teicoplanin	S	-
15.		Vancomycin	S	*van*KI, *van*K, *van*TG, *van*HM, *van*H, *van*T, *van*L
16.	Tetracycline	Tetracycline	S	*emr*A, *emr*B, *emr*Y, *emr*R, *tet*(X3), *tet*T, *tet*35, *tet*47, *ade*N, *ade*L, *ade*R,
17.		Tigecycline	S	
18.	Aminoglycoside	Gentamicin	S	*aac*(6′), *aph*(2″), *mex*S, *mex*L, *acr*S
19.	Oxazolidinone	Linezolid	S	*cfr*B, *cfr*C, *lmr*S, *poxt*A
20.	Diaminopyrimidine-Sulfonamide	Trimethoprim-Sulfamethoxazole	S	*dfr*
21.	Monoxycarbolic Acid	Mupirocin	S	*mup*, *ile*S
22.	Nitrofuran	Nitrofurantoin	S	-
23.	Rifamycin	Rifampin	S	*rpo*B2

**Table 2:** Number of potential antibiotic resistance genes involved in antibiotic resistance mechanisms detected for each SAR strain after querying the Comprehensive Antibiotic Resistance Database (CARD).

Strain *	N	Antibiotic Efflux	Antibiotic Inactivation	Antibiotic Target Alteration	Antibiotic Target Protection	Antibiotic Target Replacement
B	227	117	39	57	10	4
TS	227	119	37	55	11	5
B1	224	117	35	53	14	5
B2	219	116	33	51	14	5
B3	215	116	29	52	13	5
AR1	219	113	32	59	9	6
AR2	215	114	30	54	12	5
AR3	220	114	33	56	12	5
AR4	225	120	34	54	12	5
AR5	216	113	32	54	12	5
AR6	215	112	30	57	11	5
AR7	220	113	32	58	12	5
AR8	220	114	33	56	12	5
AR9	223	116	35	56	11	5
AR10	228	118	34	58	13	5
AR11	225	119	32	57	12	5
AR12	212	113	30	53	11	5
AR13	219	112	31	59	12	5
AR14	215	113	33	53	11	5
AR15	234	125	35	57	12	5
AR16	222	115	35	55	12	5
AR17	217	114	35	55	8	5
AR18	218	114	33	55	10	6

* B, SAR Bari strain; other abbreviations used are relative to the SAR genomes publicly available in the NCBI (see Table S1 for details).

**Table 3:** Virulence factors encoded by SAR Bari draft genome *.

Vfclass	Virulence Factors	Related Genes	Hits
1-Enzymes	Lipase	*lip*	PROKKA_00412
	Serine V8 protease	*sspA*	PROKKA_02492
	Thermonuclease	*nuc*	PROKKA_01673
2-Immune evasion	Capsule	undetermined	PROKKA_00763; PROKKA_01266; PROKKA_01584; PROKKA_02340; PROKKA_02341; PROKKA_02342; PROKKA_02343; PROKKA_02439; PROKKA_02440; PROKKA_02511;
	Capsule (*Acinetobacter*)	-	PROKKA_02505
	Polyglutamic acid capsule (*Bacillus*)	*capB*	PROKKA_01886
		*capC*	PROKKA_01887
	Polysaccharide capsule (*Bacillus*)	*galE*	PROKKA_01613
3-Antiphagocytosis	Capsule (*Klebsiella*)	*uge*	PROKKA_02504
4-Nutritional factor	Allantoin utilization (*Klebsiella*)	-	PROKKA_00370
			PROKKA_00367
			PROKKA_00364; PROKKA_00365
5-Serum resistance and immune evasion	LPS (*Francisella*)	*wbtP*	PROKKA_02442

* VFanalyzer original output.

**Table 4:** Virulence-factor genes predicted within SAR.

Strains *^.^	N	Genes
B1; B2; B3; AR1; AR10; AR11; AR12; AR13; AR14; AR15; AR16; AR17; AR18; AR2; AR3; AR4; AR5; AR6; AR7; AR8; AR9; TS; B	2	Undetermined capsule, *sspA*
B1; B2; B3; AR1; AR10; AR11; AR12; AR14; AR15; AR16; AR17; AR18; AR2; AR3; AR4; AR5; AR6; AR7; AR8; AR9; TS; B	1	*lip*
B1; B2; B3; AR10; AR11; AR12; AR13; AR14; AR15; AR16; AR17; AR18; AR2; AR3; AR4; AR5; AR6; AR7; AR8; AR9; TS; B	1	*nuc*
B1; B2; B3; AR1; AR10; AR11; AR12; AR13; AR14; AR15; AR16; AR17; AR18; AR2; AR3; AR4; AR5; AR6; AR7; AR8; AR9; B	3	*capC, capB, galE*
AR1; AR10; AR11; AR13; AR14; AR15; AR16; AR17; AR18; AR2; AR3; AR4; AR5; AR6; AR7; AR8; AR9; B	1	*wbtP*
AR10; AR11; AR12; AR13; AR14; AR15; AR16; AR2; AR3; AR4; AR5; AR6; AR7; AR8; AR9	6	*esaB, esaA, esBA, ess, essC, essA*
B2; AR10; AR13; AR3; AR7; AR8; AR9	1	*lspA*
B2; AR12; AR13; AR18; AR7; AR8	1	*gtaB*
AR12; AR13; AR7; AR8	2	*tuf, katA*
B1; B2; B3	1	*vctC*
B2; AR12; AR8	5	*plr/gapA, ndk, eno, acpBL, flmH*
B2; AR13; AR8	2	*lgt, lpeA*
AR1; AR15; AR8	1	*cylR2*
AR12; AR13; AR8	1	*slrA*
AR17; B	1	*uge*
B1; AR17	2	*icaB, icaA*
B2; AR8	1	*lisR*
B2; AR12	1	*gnd*
B2; AR13	1	*sigA/rpoV*
B3; B	1	Allantoin utilization
AR8	2	*groEL, lplA1*
AR12	1	*hemL*
AR17	1	*icaC*

* B, SAR Bari strain; other abbreviations used are relative to the SAR genomes publicly available in the NCBI (see Table S1 for details).

**Table 5:** Genes encoding virulence factors shared between SAR and other species in the dataset.

Species *	N	Genes
BS; MC; SA; SAG; SAR; SAU; SC; SCH; SE; SF; SH; SHY; SK; SS; SSC; SSI	1	*Capsule Undetermined*
SAG; SAR; SAU; SC; SCH; SE; SF; SH; SHY; SK; SS; SSI	1	*nuc*
BS; SAG; SAR; SAU; SC; SCH; SE; SH; SHY; SK; SS; SSI	2	*capB; capC*
BS; MC; SAG; SAR; SC; SCH; SE; SF; SHY; SK; SS	1	*vctC*
SA; SAR; SC; SCH; SE; SF; SH; SK; SS	1	*lip*
MC; SA; SAR; SC; SE; SK; SS; SSC; SSI	1	*icaA*
BS; MC; SAG; SAR; SCH; SF; SHY; SS; SSC	1	*lgt*
BS; SAR; SAU; SC; SF; SHY; SK; SS; SSI	1	*galE*
SA; SAR; SC; SE; SK; SS; SSC; SSI	1	*icaB*
MC; SAG; SAR; SC; SH; SHY; SK; SSI	1	*wbtP*
BS; SA; SAG; SAR; SE; SHY; SSI	1	*essC*
SA; SAR; SC; SE; SK; SS; SSC	1	*sspA*
MC; SAR; SAU; SC; SF; SH; SSI	1	*cylR2*
SA; SAG; SAR; SE; SHY; SSI	3	*esaB; essB; esxA*
MC; SAG; SAR; SCH; SHY; SSC	1	*lisR*
BS; SAG; SAR; SHY; SK; SSI	1	*lspA*
MC; SAR; SC; SH; SHY; SSI	1	*uge*
BS; SAR; SH; SHY; SK; SSI	1	*Capsule (Acinetobacter)*
SA; SAG; SAR; SE; SSI	1	*esaA*
SAR; SC; SHY; SSC; SSI	1	*Allantoin utilization (Klebsiella)*
BS; MC; SAR; SF; SS	1	*gtaB*
MC; SAR; SC; SSI	1	*LPS O-antigen (P. aeruginosa)*
BS; MC; SAR; SSC	1	*ndk*
SA; SAR; SE	1	*essA*
BS; SAR; SAU	1	*Capsule (Enterococcus)*
BS; SAR; SK	1	*LPS rfb locus (Klebsiella)*
BS; MC; SAR	1	*lplA1*
SAR; SAU	1	*lpeA*
SAR; SE	1	*eno*
BS; SAR	4	*plr/gapA; katA; acpXL; gnd*
SAR	6	*flmH; T6SS-II(Klebsiella); tuf; groEL; slrA; sigA/rpoV*

* Meanings of the abbreviations used are available in the Material and Methods (Section 2.4 Dataset). The complete list of all genes shared among all species in the dataset is available in Table S7.

## Supplementary Materials

The following are available online at https://www.mdpi.com/2076-2607/7/11/580/s1, Table S1: Genome assembly of *Staphylococcus arlettae*, Table S2: DATASET: Species and Genome Assembly, Table S3: Antibiotic resistance determinants within SAR, Table S4: Antibiotic resistance determinants shared among SAR and other species in the dataset, Table S5: Virulence elements of the SAR Bari draft genome, Table S6: Number of predicted genes correlated to virulence elements identified within species in the dataset, Table S7: Distribution of putative genes related to virulence factors identified within species in the dataset.
