# Supplementary material for: Staphylococcus arlettae Genomics: Novel Insights on Candidate Antibiotic Resistance and Virulence Genes in an Emerging Opportunistic Pathogen
Source: Microorganisms. 2019 Nov 19;7(11):580. doi: 10.3390/microorganisms7110580 (PMC6920755; doi:10.3390/microorganisms7110580)
Supplement: Supplementary file 1 [file microorganisms-07-00580-s001.zip › Table S1.docx]

| Organism name | Strain | Assembly | Size (Mb) | Gene | Protein | Release | Level | Abbreviation  (this study) |
| --- | --- | --- | --- | --- | --- | --- | --- | --- |
| Staphylococcus arlettae CVD059 | CVD059 | GCA_000295715.1 | 2.56568 | 2524 | 2308 | 10/09/2012 | Contig | AR1 |
| Staphylococcus arlettae | NCTC12413 | GCA_900457375.1 | 2.7227 | 2666 | 2541 | 30/07/2018 | Contig | TS |
| Staphylococcus arlettae | NCTC 12413 | GCA_002902345.1 | 2.53822 | 2591 | 2500 | 27/01/2018 | Scaffold | - |
| Staphylococcus arlettae | SNUC 1330 | GCA_003040795.1 | 2.60738 | 2463 | 2347 | 05/04/2018 | Contig | AR2 |
| Staphylococcus arlettae | IOV5 | GCA_004570895.1 | 2.61239 | 2591 | 2436 | 02/04/2019 | Contig | AR17 |
| Staphylococcus arlettae | Bari1 | GCA_003290105.1 | 2.4989 | 2582 | 2389 | 06/07/2018 | Contig | B1 |
| Staphylococcus arlettae | Bari2 | GCA_003290035.1 | 2.72491 | 2442 | 2267 | 06/07/2018 | Contig | B2 |
| Staphylococcus arlettae | SNUC 4786 | GCA_003040735.1 | 2.62605 | 2684 | 2568 | 05/04/2018 | Contig | AR3 |
| Staphylococcus arlettae | SNUC 4935 | GCA_003578645.1 | 2.49015 | 2556 | 2430 | 20/09/2018 | Contig | AR4 |
| Staphylococcus arlettae | Bari3 | GCA_003290115.1 | 2.54222 | 2430 | 2250 | 06/07/2018 | Contig | B3 |
| Staphylococcus arlettae | SNUC 4426 | GCA_003040775.1 | 2.54784 | 2472 | 2362 | 05/04/2018 | Contig | AR5 |
| Staphylococcus arlettae | SNUC 3447 | GCA_003041115.1 | 2.67355 | 2495 | 2372 | 05/04/2018 | Contig | AR6 |
| Staphylococcus arlettae | SNUC 1715.2 | GCA_003040755.1 | 2.61021 | 2646 | 2506 | 05/04/2018 | Contig | AR7 |
| Staphylococcus arlettae | SNUC 3029 | GCA_003041155.1 | 2.5305 | 2557 | 2435 | 05/04/2018 | Contig | AR8 |
| Staphylococcus arlettae | SNUC 2101 | GCA_003041135.1 | 2.63012 | 2465 | 2373 | 05/04/2018 | Contig | AR9 |
| Staphylococcus arlettae | SNUC 1576 | GCA_003041175.1 | 2.60443 | 2549 | 2432 | 05/04/2018 | Contig | AR10 |
| Staphylococcus arlettae | SNUC 3131 | GCA_003578665.1 | 2.5203 | 2576 | 2449 | 20/09/2018 | Contig | AR11 |
| Staphylococcus arlettae | SNUC 5134 | GCA_003579095.1 | 2.61355 | 2471 | 2343 | 20/09/2018 | Contig | AR12 |
| Staphylococcus arlettae | SNUC 4292 | GCA_003041095.1 | 2.60199 | 2586 | 2424 | 05/04/2018 | Contig | AR13 |
| Staphylococcus arlettae | SNUC 4202 | GCA_003578655.1 | 2.6151 | 2650 | 2443 | 20/09/2018 | Contig | AR14 |
| Staphylococcus arlettae | SNUC 1480 | GCA_003578705.1 | 2.47962 | 2640 | 2463 | 20/09/2018 | Contig | AR15 |
| Staphylococcus arlettae | SNUC 1401 | GCA_003578715.1 | 2.65226 | 2528 | 2337 | 20/09/2018 | Contig | AR16 |
| Staphylococcus arlettae | P2 | GCA_006741705.1 |  | 2633 | 2550 | 03/06/2019 | Complete genome | AR18 |

**Table S1.** Genome assembly of *Staphylococcus arlettae*^*^

^*^Data are from the NCBI original table (https://www.ncbi.nlm.nih.gov/genome/genomes/11830?) related to June, 2019. Abbreviations used in this study were added.
