# Supplementary material for: Staphylococcus arlettae Genomics: Novel Insights on Candidate Antibiotic Resistance and Virulence Genes in an Emerging Opportunistic Pathogen
Source: Microorganisms. 2019 Nov 19;7(11):580. doi: 10.3390/microorganisms7110580 (PMC6920755; doi:10.3390/microorganisms7110580)
Supplement: Supplementary file 1 [file microorganisms-07-00580-s001.zip › Table S2.docx]

**Table S2.** DATASET: Species and Genome Assembly^*^

|  | Strain Abbreviation | Organism Name | Strain | Assembly |
| --- | --- | --- | --- | --- |
| SAR | B | *Staphylococcus arlettae* | Bari | (This study) ^§^ |
|  | B1 | *Staphylococcus arlettae* | BARI1 | GCA_003290105.1 |
|  | B2 | *Staphylococcus arlettae* | BARI2 | GCA_003290035.1 |
|  | B3 | *Staphylococcus arlettae* | BARI3 | GCA_003290115.1 |
|  | TSAR | *Staphylococcus arlettae* | NCTC12413 | GCA_900457375.1 |
|  | AR1 | *Staphylococcus arlettae CVD059* | CVD059 | GCA_000295715.1 |
|  | AR2 | *Staphylococcus arlettae* | SNUC 4786 | GCA_003040735.1 |
|  | AR3 | *Staphylococcus arlettae* | SNUC 1715.2 | GCA_003040755.1 |
|  | AR4 | *Staphylococcus arlettae* | SNUC 4426 | GCA_003040775.1 |
|  | AR5 | *Staphylococcus arlettae* | SNUC 1330 | GCA_003040795.1 |
|  | AR6 | *Staphylococcus arlettae* | SNUC 4292 | GCA_003041095.1 |
|  | AR7 | *Staphylococcus arlettae* | SNUC 3447 | GCA_003041115.1 |
|  | AR8 | *Staphylococcus arlettae* | SNUC 2101 | GCA_003041135.1 |
|  | AR9 | *Staphylococcus arlettae* | SNUC 3029 | GCA_003041155.1 |
|  | AR10 | *Staphylococcus arlettae* | SNUC 1576 | GCA_003041175.1 |
| SA | TSA1 | *Staphylococcus aureus subsp. anaerobius* | DSM 20714 | GCA_002902425.1 |
|  | TSA2 | *Staphylococcus aureus subsp. aureus DSM 20231* | DSM 20231 | GCA_001027105.1 |
|  | SA1 | *Staphylococcus aureus RF122* | RF122 | GCA_000009005.1 |
|  | SA2 | *Staphylococcus aureus subsp. aureus N315* | N315 | GCA_000009645.1 |
|  | SA3 | *Staphylococcus aureus subsp. aureus JH1* | JH1 | GCA_000017125.1 |
|  | SA4 | *Staphylococcus aureus subsp. aureus Mu50* | Mu50 | GCA_000009665.1 |
|  | SA5 | *Staphylococcus aureus subsp. aureus Mu3* | Mu3 | GCA_000010445.1 |
|  | SA6 | *Staphylococcus aureus subsp. aureus str. Newman* | Newman | GCA_000010465.1 |
|  | SA7 | *Staphylococcus aureus subsp. aureus COL* | COL | GCA_000012045.1 |
|  | SA8 | *Staphylococcus aureus subsp. aureus* | Gv69 | GCA_000769575.1 |
|  | SA9 | *Staphylococcus aureus subsp. aureus ST772-MRSA-V* | DAR4145 | GCA_000828035.1 |
|  | SA10 | *Staphylococcus aureus* | ILRI_Eymole1/1 | GCA_000953255.1 |
|  | SA11 | *Staphylococcus aureus subsp. Anaerobius* | Mq2T | GCA_000981205.1 |
| SE | TSE | *Staphylococcus epidermidis* | ATCC 14990 | GCA_002087975.1 |
|  | SE1 | *Staphylococcus epidermidis ATCC 12228* | ATCC 12228 | GCA_000007645.1 |
|  | SE2 | *Staphylococcus epidermidis PM221* | PM221 | GCA_000751035.1 |
|  | SE3 | *Staphylococcus epidermidis* | FDAARGOS_153 | GCA_002944995.1 |
|  | SE4 | *Staphylococcus epidermidis* | NCTC4133 | GCA_900636255.1 |
|  | SE5 | *Staphylococcus epidermidis W23144* | W23144 | GCA_000160235.1 |
|  | SE6 | *Staphylococcus epidermidis M0881* | M0881 | GCA_000362145.1 |
|  | SE7 | *Staphylococcus epidermidis* | C10C | GCA_000763625.1 |
|  | SE8 | *Staphylococcus epidermidis VCU037* | VCU037 | GCA_000221705.2 |
|  | SE9 | *Staphylococcus epidermidis IS-250* | IS-250 | GCA_000257945.1 |
|  | SE10 | *Staphylococcus epidermidis VCU111* | VCU111 | GCA_000697805.1 |
| SH | TSH | *Staphylococcus haemolyticus* | NCTC11042 | GCA_900458595.1 |
|  | SH1 | *Staphylococcus haemolyticus JCSC1435* | JCSC1435 | GCA_000009865.1 |
|  | SH2 | *Staphylococcus haemolyticus* | S167 | GCA_001611955.1 |
|  | SH3 | *Staphylococcus haemolyticus* | 83131A | GCA_002906595.1 |
|  | SH4 | *Staphylococcus haemolyticus* | SGAir0252 | GCA_002952715.2 |
|  | SH5 | *Staphylococcus haemolyticus* | FDAARGOS_517 | GCA_003956005.1 |
|  | SH6 | *Staphylococcus haemolyticus DNF00585* | DNF00585 | GCA_000759125.1 |
|  | SH7 | *Staphylococcus haemolyticus* | 1HT3 | GCA_000981145.1 |
|  | SH8 | *Staphylococcus haemolyticus* | MTCC 3383 | GCA_001412465.1 |
|  | SH9 | *Staphylococcus haemolyticus* | FDAARGOS_130 | GCA_001471515.2 |
|  | SH10 | *Staphylococcus haemolyticus* | 864-1 | GCA_001705095.1 |
| SS | TSS1 | *Staphylococcus saprophyticus subsp. bovis* | CCUG 38042 | GCA_002902545.1 |
|  | TSS2 | *Staphylococcus saprophyticus subsp. saprophyticus ATCC 15305* | ATCC 15305 | GCA_000010125.1 |
|  | SS1 | *Staphylococcus saprophyticus* | 82C | GCA_900097975.1 |
|  | SS2 | *Staphylococcus saprophyticus* | FDAARGOS_168 | GCA_001558275.2 |
|  | SS3 | *Staphylococcus saprophyticus* | FDAARGOS_137 | GCA_001558375.2 |
|  | SS4 | *Staphylococcus saprophyticus* | FDAARGOS_336 | GCA_002208905.2 |
|  | SS5 | *Staphylococcus saprophyticus* | FDAARGOS_355 | GCA_002209265.2 |
|  | SS6 | *Staphylococcus saprophyticus subsp. saprophyticus* | NCTC7666 | GCA_900635295.1 |
|  | SS7 | *Staphylococcus saprophyticus* | DPC5671 | GCA_002009035.1 |
|  | SS8 | *Staphylococcus saprophyticus* | 725A_RS6 | GCA_001747665.1 |
|  | SS9 | *Staphylococcus saprophyticus* | AG1 | GCA_004100065.1 |
|  | SS10 | *Staphylococcus saprophyticus* | SS116 | GCA_004122665.1 |
| SK | TSK | *Staphylococcus kloosii* | ATCC 43959 | GCA_003019255.1 |
|  | K1 | *Staphylococcus kloosii* | CNV2 | GCA_001593625.1 |
|  | K2 | *Staphylococcus kloosii* | SNUC 4696 | GCA_003042035.1 |
|  | K3 | *Staphylococcus kloosii* | NCTC12415 | GCA_900458805.1 |
|  | K4 | *Staphylococcus kloosii* | NCTC 12415 | GCA_002902055.1 |
| SC | TSC1 | *Staphylococcus cohnii* | NCTC11041 | GCA_900458255.1 |
|  | TSC2 | *Staphylococcus cohnii subsp. urealyticus* | DSM 6718 | GCA_002902235.1 |
|  | C1 | *Staphylococcus cohnii subsp. cohnii* | 532 | GCA_000972575.1 |
|  | C2 | *Staphylococcus cohnii* | FDAARGOS_334 | GCA_002984565.1 |
|  | C3 | *Staphylococcus cohnii* | FDAARGOS_538 | GCA_003956025.1 |
|  | C4 | *Staphylococcus cohnii subsp. cohnii* | 57 | GCA_000972565.1 |
|  | C5 | *Staphylococcus cohnii subsp. cohnii* | G22B2 | GCA_000981215.1 |
|  | C6 | *Staphylococcus cohnii* | H62 | GCA_001650645.1 |
|  | C7 | *Staphylococcus cohnii* | MF1844 | GCA_001651275.1 |
|  | C8 | *Staphylococcus cohnii* | SE4.1 | GCA_001876705.1 |
|  | C9 | *Staphylococcus cohnii* | SE3.10 | GCA_001876725.1 |
|  | C10 | *Staphylococcus cohnii* | SE4.2 | GCA_001876735.1 |
| SAU | TSAU | *Staphylococcus auricularis* | NCTC12101 | GCA_900478415.1 |
|  | SAU1 | *Staphylococcus auricularis* | SNUC 3034 | GCA_003040695.1 |
|  | SAU2 | *Staphylococcus auricularis* | SNUC 993 | GCA_003040715.1 |
|  | SAU3 | *Staphylococcus auricularis* | NCTC 12101 | GCA_002902455.1 |
| SHY | TSHY | *Staphylococcus hyicus* | ATCC 11249 | GCA_000816085.1 |
|  | HY1 | *Staphylococcus hyicus* | NCTC10350 | GCA_900474585.1 |
|  | HY2 | *Staphylococcus hyicus* | NCTC7944 | GCA_900636345.1 |
|  | HY3 | *Staphylococcus hyicus* | SNUC 5426 | GCA_003042065.1 |
|  | HY4 | *Staphylococcus hyicus* | SNUC 4992 | GCA_003042905.1 |
| SCH | TSCH | *Staphylococcus chromogenes* | NCTC10530 | GCA_900458195.1 |
|  | CH1 | *Staphylococcus chromogenes MU 970* | MU 970 | GCA_000696815.1 |
|  | CH2 | *Staphylococcus chromogenes* | SNUC 4584 | GCA_003036025.1 |
|  | CH3 | *Staphylococcus chromogenes* | SNUC 5997 | GCA_003036035.1 |
|  | CH4 | *Staphylococcus chromogenes* | SNUC 5084 | GCA_003036065.1 |
| SAG | TSAG | *Staphylococcus agnetis* | DSM 23656 | GCA_002901865.1 |
|  | AG1 | *Staphylococcus agnetis* | 908 | GCA_001442815.1 |
|  | AG2 | *Staphylococcus agnetis* | CBMRN20813338 | GCA_000737185.1 |
|  | AG3 | *Staphylococcus agnetis* | 722_260714_1_8_heart | GCA_002114335.1 |
|  | AG4 | *Staphylococcus agnetis* | 722_230714_2_5_spleen | GCA_002114365.1 |
| SF | TSF | *Staphylococcus felis* | DSM 7377 | GCA_002902185.1 |
|  | F1 | *Staphylococcus felis* | ATCC 49168 | GCA_003012915.1 |
|  | F2 | *Staphylococcus felis* | F19 | GCA_003385325.1 |
|  | F3 | *Staphylococcus felis* | F17 | GCA_003385345.1 |
|  | F4 | *Staphylococcus felis* | F4 | GCA_003385435.1 |
| SI | TSSI | *Staphylococcus simulans* | NCTC11046 | GCA_900474685.1 |
|  | SI1 | *Staphylococcus simulans* | FDAARGOS_124 | GCA_001559115.2 |
|  | SI2 | *Staphylococcus simulans* | FDAARGOS_383 | GCA_002386185.1 |
|  | SI3 | *Staphylococcus simulans* | MR3 | GCA_003006055.1 |
|  | SI4 | *Staphylococcus simulans* | MR4 | GCA_003006075.1 |
|  | SI5 | *Staphylococcus simulans* | MR2 | GCA_003076375.1 |
|  | SI6 | *Staphylococcus simulans* | MR1 | GCA_003096155.1 |
|  | SI7 | *Staphylococcus simulans ACS-120-V-Sch1* | ACS-120-V-Sch1 | GCA_000314755.2 |
|  | SI8 | *Staphylococcus simulans* | MJR7712 | GCA_001546635.1 |
|  | SI9 | *Staphylococcus simulans UMC-CNS-990* | UMC-CNS-990 | GCA_000477455.1 |
|  | SI10 | *Staphylococcus simulans* | SNUC 5405 | GCA_003041655.1 |
| SSC | TSSC | *Staphylococcus sciuri subsp. sciuri* | NCTC12103 | GCA_900474615.1 |
|  | SC1 | *Staphylococcus sciuri* | FDAARGOS_285 | GCA_002209165.2 |
|  | SC2 | *Staphylococcus sciuri* | NS202 | GCA_001476555.1 |
|  | SC3 | *Staphylococcus sciuri* | NS53 | GCA_001476575.1 |
|  | SC4 | *Staphylococcus sciuri* | RSA37 | GCA_001476585.1 |
|  | SC5 | *Staphylococcus sciuri* | NS1 | GCA_001476955.1 |
|  | SC6 | *Staphylococcus sciuri* | NS36 | GCA_001477335.1 |
|  | SC7 | *Staphylococcus sciuri* | NS112 | GCA_001477395.1 |
|  | SC8 | *Staphylococcus sciuri* | NS44 | GCA_001477405.1 |
|  | SC9 | *Staphylococcus sciuri* | SAP15-1 | GCA_001684285.1 |
|  | SC10 | *Staphylococcus sciuri* | P575 | GCA_001766775.1 |
| BS | TSBS1 | *Bacillus subtilis subsp. inaquosorum* | KCTC 13429 | GCA_003148415.1 |
|  | TSBS2 | *Bacillus subtilis subsp. spizizenii TU-B-10* | TU-B-10 | GCA_000227465.1 |
|  | TSBS3 | *Bacillus subtilis* | NCIB 3610 | GCA_002055965.1 |
|  | BS1 | *Bacillus subtilis subsp. subtilis str. 168* | 168 | GCA_000009045.1 |
|  | BS2 | *Bacillus subtilis subsp. spizizenii str. W23* | W23 | GCA_000146565.1 |
|  | BS3 | *Bacillus subtilis BSn5* | BSn5 | GCA_000186745.1 |
|  | BS4 | *Bacillus subtilis subsp. natto BEST195* | BEST195 | GCA_000209795.2 |
|  | BS5 | *Bacillus subtilis subsp. subtilis str. RO-NN-1* | RO-NN-1 | GCA_000227485.1 |
|  | BS6 | *Bacillus subtilis QB928* | QB928 | GCA_000293765.1 |
|  | BS7 | *Bacillus subtilis subsp. subtilis str. BSP1* | BSP1 | GCA_000321395.1 |
|  | BS8 | *Bacillus subtilis XF-1* | XF-1 | GCA_000338735.1 |
|  | BS9 | *Bacillus subtilis subsp. subtilis 6051-HGW* | 6051-HGW | GCA_000344745.1 |
|  | BS10 | *Bacillus subtilis subsp. subtilis str. BAB-1* | BAB-1 | GCA_000349795.1 |
| MC | TSMC | *Macrococcus caseolyticus* | DSM 20597 | GCA_002902665.1 |
|  | MC1 | *Macrococcus caseolyticus JCSC5402* | JCSC5402 | GCA_000010585.1 |
|  | MC2 | *Macrococcus caseolyticus* | IMD0819 | GCA_002119825.1 |
|  | MC3 | *Macrococcus caseolyticus subsp. hominis* | CCM 7927 | GCA_002742395.2 |
|  | MC4 | *Macrococcus caseolyticus* | 5815_BC85 | GCA_002834575.1 |
|  | MC5 | *Macrococcus caseolyticus* | 5814_BC75 | GCA_002834595.1 |
|  | MC6 | *Macrococcus caseolyticus* | 5813_BC74 | GCA_002834615.1 |
|  | MC7 | *Macrococcus caseolyticus* | 5804_BC29 | GCA_002834635.1 |
|  | MC8 | *Macrococcus caseolyticus* | 5798_EF375 | GCA_002834665.1 |
|  | MC9 | *Macrococcus caseolyticus* | 5795_EF335 | GCA_002834675.1 |
|  | MC10 | *Macrococcus caseolyticus* | 5794_EF323 | GCA_002834705.1 |

* Organism name, strain and assembly are indicated as reported in NCBI-Genome (https://www.ncbi.nlm.nih.gov/genome). ^§^Genome assembly under NCBI submission.
