# Supplementary material for: Staphylococcus arlettae Genomics: Novel Insights on Candidate Antibiotic Resistance and Virulence Genes in an Emerging Opportunistic Pathogen
Source: Microorganisms. 2019 Nov 19;7(11):580. doi: 10.3390/microorganisms7110580 (PMC6920755; doi:10.3390/microorganisms7110580)
Supplement: Supplementary file 1 [file microorganisms-07-00580-s001.zip › Table S3.docx]

**Table S3**. Antibiotic resistance determinants within SAR

| SAR strains | N | Antibiotic resistance determinants |
| --- | --- | --- |
| B1; B2; B3; AR1; AR10; AR11; AR12; AR13; AR14; AR15; AR16; AR17; AR18; AR2; AR3; AR4; AR5; AR6; AR7; AR8; AR9; TS; B | 94 | *golS; Mycobacterium tuberculosis inhA mutations conferring resistance to isoniazid; Clostridioides difficile gyrA conferring resistance to fluoroquinolones; tetA(60); Bacillus subtilis pgsA with mutation conferring resistance to daptomycin; arlR; Streptococcus pneumoniae PBP2x conferring resistance to amoxicillin; abeS; vanKI; rphB; vanHA; emrR; tet(35); TaeA; evgS; nalD; lmrB; Mycobacterium tuberculosis kasA mutant conferring resistance to isoniazid; vanTG; PmrF; tsnR; optrA ; dfrC; vanYM; ykkD; arnA; msbA; Acinetobacter baumannii AbaF; Acinetobacter baumannii AbaQ; SAT-3; Escherichia coli soxS with mutation conferring antibiotic resistance; vanRI; sul4; MexS; norB; AcrS; rpoB2; bcr-1; tetB(60); Staphylococcus aureus gyrB conferring resistance to aminocoumarin; baeS; otr(A); bcrA; arlS; Staphylococcus mupA conferring resistance to mupirocin; FusF; patA; norA; nalC; vatB; AAC(6')-Iw; Staphylococcys aureus LmrS; NmcR; vatF; vanRM; Enterococcus faecalis cls with mutation conferring resistance to daptomycin; Staphylococcus aureus murA with mutation conferring resistance to fosfomycin; Escherichia coli fabI mutations conferring resistance to isoniazid and triclosan; vanHO; bacA; gadX; vanHM; novA; emrA; mgrA; pmrA; tetT; emeA; gimA; ampS; ykkC; vanL; evgA; mecD; adeL; Clostridioides difficile gyrB conferring resistance to fluoroquinolone; Staphylococcus aureus fusA with mutation conferring resistance to fusidic acid; Borreliella burgdorferi murA with mutation conferring resistance to fosfomycin; Escherichia coli EF-Tu mutants conferring resistance to kirromycin; cmlv; mecR1; gadW; DHA-1; tetB(P); mtrA; CAU-1; Mycobacterium tuberculosis pncA mutations conferring resistance to pyrazinamide; cfrC; vanHB; vanE; macB; fexA; Erm(K); mecI;* |
| B1; B2; B3; AR1; AR10; AR11; AR12; AR13; AR14; AR15; AR16; AR18; AR2; AR3; AR4; AR5; AR6; AR7; AR8; AR9; TS; B | 1 | *salA* |
| B1; B2; B3; AR1; AR10; AR11; AR12; AR13; AR14; AR16; AR17; AR18; AR2; AR3; AR4; AR5; AR6; AR7; AR8; AR9; TS; B | 1 | *SAT-2* |
| B1; B2; B3; AR1; AR10; AR11; AR13; AR14; AR15; AR16; AR17; AR18; AR2; AR3; AR4; AR5; AR6; AR7; AR8; AR9; TS; B | 1 | *mdtG* |
| B1; B2; B3; AR1; AR11; AR12; AR13; AR14; AR15; AR16; AR17; AR18; AR2; AR3; AR4; AR5; AR6; AR7; AR8; AR9; TS; B | 1 | *FosA6* |
| B1; B2; B3; AR1; AR10; AR11; AR12; AR13; AR14; AR15; AR16; AR17; AR18; AR2; AR3; AR4; AR5; AR6; AR7; AR9; TS; B | 1 | *mdtH* |
| B1; B2; B3; AR1; AR10; AR11; AR12; AR13; AR14; AR15; AR16; AR17; AR18; AR2; AR3; AR5; AR6; AR7; AR8; AR9; TS; B | 1 | *Bacillus; subtilis; mprF* |
| B1; B2; B3; AR10; AR11; AR12; AR13; AR14; AR15; AR16; AR17; AR18; AR2; AR3; AR4; AR5; AR6; AR7; AR8; AR9; TS; B | 1 | *Tet(47)* |
| B1; B2; AR1; AR10; AR11; AR12; AR13; AR14; AR15; AR16; AR17; AR18; AR2; AR3; AR4; AR5; AR6; AR7; AR8; AR9; TS; B | 1 | *sta* |
| B1; B2; B3; AR1; AR10; AR11; AR12; AR13; AR14; AR15; AR16; AR2; AR3; AR4; AR5; AR6; AR7; AR8; AR9; TS; B | 1 | *adeR* |
| B1; B2; B3; AR1; AR10; AR11; AR12; AR13; AR14; AR16; AR17; AR2; AR3; AR4; AR5; AR6; AR7; AR8; AR9; TS; B | 1 | *GOB-18* |
| B1; B2; B3; AR1; AR10; AR11; AR14; AR15; AR16; AR17; AR18; AR2; AR3; AR4; AR5; AR6; AR7; AR8; AR9; TS; B | 1 | *vatH* |
| B1; B2; B3; AR1; AR10; AR11; AR13; AR14; AR15; AR16; AR17; AR18; AR2; AR3; AR4; AR5; AR6; AR8; AR9; TS; B | 1 | *poxtA* |
| B1; B2; B3; AR1; AR10; AR11; AR12; AR13; AR14; AR15; AR16; AR17; AR2; AR3; AR4; AR5; AR6; AR7; AR8; AR9; TS | 3 | *tetA(48); IMP-31; PC1; beta-lactamase (blaZ)* |
| B1; B2; B3; AR1; AR10; AR11; AR12; AR13; AR14; AR15; AR16; AR18; AR2; AR3; AR4; AR5; AR6; AR7; AR8; AR9; TS | 1 | *Staphylococcus mupB conferring resistance to mupirocin* |
| B1; B2; B3; AR10; AR11; AR12; AR13; AR14; AR15; AR16; AR2; AR3; AR4; AR5; AR6; AR7; AR8; AR9; TS; B | 1 | *lmrC* |
| B1; B2; AR10; AR11; AR12; AR13; AR14; AR15; AR16; AR17; AR2; AR3; AR4; AR5; AR6; AR7; AR8; AR9; TS; B | 1 | *FosD* |
| B1; B2; B3; AR1; AR10; AR11; AR12; AR13; AR14; AR15; AR16; AR18; AR2; AR3; AR4; AR5; AR6; AR7; AR8; AR9 | 1 | *Escherichia coli emrE* |
| B1; B2; AR1; AR10; AR11; AR12; AR13; AR14; AR15; AR16; AR18; AR2; AR3; AR4; AR5; AR6; AR7; AR8; AR9; TS | 1 | *AAC(6')-I30* |
| AR1; AR10; AR11; AR13; AR14; AR15; AR16; AR17; AR18; AR2; AR3; AR4; AR5; AR6; AR7; AR8; AR9; TS; B | 1 | *ugd* |
| AR1; AR10; AR11; AR12; AR13; AR14; AR15; AR16; AR17; AR18; AR2; AR3; AR4; AR5; AR6; AR7; AR8; AR9; B | 1 | *lmrD* |
| B1; B2; B3; AR10; AR11; AR12; AR13; AR16; AR17; AR2; AR3; AR4; AR5; AR7; AR8; AR9; TS; B | 1 | *LRA-2* |
| AR1; AR10; AR11; AR13; AR14; AR16; AR17; AR18; AR2; AR3; AR4; AR5; AR6; AR7; AR8; AR9; TS; B | 1 | *oleC* |
| B1; B2; AR1; AR10; AR11; AR12; AR13; AR14; AR15; AR18; AR2; AR3; AR4; AR5; AR6; AR8; AR9; TS | 1 | *msrA* |
| B1; B2; B3; AR1; AR10; AR12; AR13; AR14; AR15; AR2; AR3; AR4; AR8; AR9; TS; B | 1 | *blt* |
| AR1; AR10; AR11; AR12; AR13; AR16; AR17; AR2; AR3; AR4; AR5; AR6; AR7; AR8; AR9; B | 1 | *adeN* |
| AR1; AR10; AR12; AR13; AR14; AR15; AR17; AR18; AR2; AR3; AR4; AR9; TS; B | 1 | *arr-1* |
| AR10; AR11; AR13; AR14; AR15; AR16; AR2; AR3; AR4; AR5; AR6; AR7; AR9 | 1 | *tet(49)* |
| AR1; AR10; AR12; AR13; AR17; AR18; AR2; AR3; AR4; AR9; TS; B | 1 | *Tet(X3)* |
| AR10; AR11; AR13; AR14; AR16; AR2; AR3; AR4; AR5; AR6; AR8; AR9 | 1 | *vanSC* |
| B1; B2; B3; AR10; AR14; AR15; AR3; AR4; AR6; AR9; TS | 1 | *catB9* |
| AR1; AR11; AR13; AR16; AR17; AR18; AR2; AR5; AR7; B | 1 | *catB3* |
| AR10; AR11; AR13; AR14; AR16; AR17; AR6; AR9; TS | 1 | *tetU* |
| AR1; AR13; AR15; AR2; AR3; AR6; AR8; AR9 | 1 | *vanSF* |
| AR12; AR13; AR14; AR15; AR2; AR3; AR4; AR9 | 1 | *otr(B)* |
| AR1; AR10; AR12; AR14; AR15; AR6; AR8 | 1 | *LRA-8* |
| AR11; AR16; AR17; AR18; AR5; AR7 | 1 | *Staphylococcus aureus norA* |
| B1; B2; B3; AR10; B | 1 | *tlrC* |
| AR10; AR11; AR13; AR14; TS | 1 | *tet(K)* |
| AR10; AR14; AR15; AR3; AR6 | 1 | *vanWG* |
| B1; B2; B3; B | 2 | *cmeR; qacB* |
| AR14; AR15; AR6; AR8 | 1 | *Tet(X4)* |
| AR1; AR17; B | 1 | *Bifidobacterium ileS conferring resistance to mupirocin* |
| AR11; AR15; B | 1 | *spd* |
| B1; B2; B3 | 3 | *catA8 Corynebacterium striatum; tetA; tetB(46)* |
| B1; B2; TS | 1 | *mphC* |
| B1; B3; AR18 | 1 | *AAC(6')-IIa* |
| AR1; AR15; AR18 | 1 | *PEDO-2* |
| AR12; AR15; AR7 | 1 | *vanSG* |
| AR11; AR14; AR15 | 1 | *lnuA* |
| AR11; B | 1 | *FosB5* |
| AR17; B | 1 | *ErmC* |
| AR18; B | 3 | *IMP-35; tetA(58); GOB-2* |
| AR1; AR18 | 2 | *vanHD; dfrG* |
| AR12; AR2 | 1 | *efmA* |
| AR3; AR4 | 1 | *Salmonella serovars soxS with mutation conferring antibiotic resistance* |
| AR7; AR8 | 1 | *vanUG* |
| AR12; AR14 | 1 | *GOB-1* |
| AR13; AR14 | 1 | *lmrP* |
| B | 4 | *MexL; AAC(6')-34; ARL-1; AAC(6')-Ie-APH(2'')-Ia* |
| TS | 3 | *EdeQ; aadK; Enterococcus faecium chloramphenicol acetyltransferase* |
| AR1 | 2 | *tet(48); otrC* |
| AR4 | 1 | *tet36* |
| AR7 | 2 | *mepA; vanSN* |
| AR8 | 2 | *AAC(6')-Ip; catB11* |
| AR10 | 1 | *FosB3* |
| AR13 | 1 | *vatA* |
| AR14 | 3 | *SMB-1; vanSA; efrA* |
| AR17 | 3 | *QepA4; ANT(6)-Ia; Escherichia coli acrR with mutation conferring multidrug antibiotic resistance* |
| AR18 | 3 | *ARL-2; BJP-1; tva(A)* |

^*^N is the number of antibiotic resistance determinants shared among SAR strains: **B**, Bari; **TS**, type strain NCTC 12413; **B1**, BARI1; **B2**, BARI2; **B3**, BARI3; **AR1**, CVD059; **AR2**, SNUC1330; **AR3**, SNUC4786; **AR4**, SNUC4935; **AR5**, SNUC4426; **AR6**, SNUC3447; **AR7**, SNUC1715.2; **AR8**, SNUC3029; **AR9**, SNUC2101; **AR10**, SNUC1576; **AR11**, SNUC3131; **AR12**, SNUC5134; **AR13**, SNUC4292; **AR14**, SNUC4202; **AR15**, SNUC1480; **AR16**, SNUC1401; **AR17**, IOV5; **AR18**, P2.
