# Supplementary material for: Staphylococcus arlettae Genomics: Novel Insights on Candidate Antibiotic Resistance and Virulence Genes in an Emerging Opportunistic Pathogen
Source: Microorganisms. 2019 Nov 19;7(11):580. doi: 10.3390/microorganisms7110580 (PMC6920755; doi:10.3390/microorganisms7110580)
Supplement: Supplementary file 1 [file microorganisms-07-00580-s001.zip › Table S4.docx]

**Table S4.** Antibiotic resistance determinants shared among SAR and other species dataset

| Species^*^ | N | Antibiotic resistance determinants |
| --- | --- | --- |
| SAG; SAR; BS; SC; SCH; SF; SHY; SK; MC; SA; SAU; SSC; SE; SH; SI; SS | 36 | Clostridioides difficile gyrA conferring resistance to fluoroquinolones; arlR; Streptococcus pneumoniae PBP2x conferring resistance to amoxicillin; tet(35); TaeA; tsnR; arlS; vatB; Staphylococcys aureus LmrS; Enterococcus faecalis cls with mutation conferring resistance to daptomycin; novA; mtrA; Mycobacterium tuberculosis pncA mutations conferring resistance to pyrazinamide; macB; rphB; vanTG; PmrF; optrA; arnA; msbA; MexS; sul4; bcr-1; bcrA; tetA(48); Staphylococcus mupA conferring resistance to mupirocin; NmcR; Escherichia coli fabI mutations conferring resistance to isoniazid and triclosan; bacA; mgrA; tetT; ampS; evgA; Staphylococcus mupB conferring resistance to mupirocin; Clostridioides difficile gyrB conferring resistance to fluoroquinolone; DHA-1 |
| SAG; SAR; BS; SC; SCH; SF; SHY; SK; MC; SA; SAU; SSC; SE; SH; SS | 1 | adeL |
| SAG; SAR; BS; SC; SCH; SF; SHY; SK; SA; SAU; SSC; SE; SH; SI; SS | 2 | Acinetobacter baumannii AbaQ; otr(A) |
| SAG; SAR; BS; SC; SCH; SHY; SK; MC; SA; SAU; SSC; SE; SH; SI; SS | 4 | golS; baeS; Bacillus subtilis mprF; Erm(K) |
| SAG; SAR; SC; SCH; SF; SHY; SK; MC; SA; SAU; SSC; SE; SH; SI; SS | 6 | dfrC; norB; Staphylococcus aureus fusA with mutation conferring resistance to fusidic acid; Escherichia coli EF-Tu mutants conferring resistance to kirromycin; vanKI; vanHO |
| SAG; SAR; BS; SC; SCH; SF; SHY; SK; MC; SA; SSC; SE; SH; SI; SS | 2 | vanRI; tetB(60) |
| SAG; SAR; BS; SC; SCH; SF; SHY; SK; MC; SAU; SSC; SE; SH; SI; SS | 2 | ykkD; ykkC |
| SAR; BS; SC; SCH; SF; SHY; SK; MC; SA; SAU; SSC; SE; SH; SI; SS | 1 | vanHA |
| SAG; SAR; BS; SCH; SF; SHY; SK; MC; SA; SAU; SSC; SE; SI; SS | 1 | emrR |
| SAG; SAR; SC; SCH; SF; SHY; SK; SA; SAU; SSC; SE; SH; SI; SS | 1 | poxtA |
| SAG; SAR; SC; SCH; SHY; SK; MC; SA; SAU; SSC; SE; SH; SI; SS | 1 | vanL |
| SAG; SAR; SCH; SF; SHY; SK; MC; SA; SAU; SSC; SE; SH; SI; SS | 1 | tetB(P) |
| SAG; SAR; SC; SCH; SF; SHY; SK; MC; SA; SSC; SE; SH; SI; SS | 1 | mecD |
| SAG; SAR; BS; SC; SCH; SF; SHY; SK; SAU; SSC; SE; SH; SI; SS | 1 | fexA |
| SAG; SAR; BS; SC; SCH; SHY; SK; MC; SAU; SSC; SE; SH; SI; SS | 1 | cmlv |
| SAG; BS; SC; SCH; SF; SHY; SK; SA; SAU; SSC; SE; SH; SI; SS | 1 | vanRF |
| SAR; BS; SC; SCH; SF; SHY; MC; SA; SAU; SSC; SE; SH; SI; SS | 1 | oleC |
| SAR; BS; SC; SCH; SF; SHY; SK; MC; SAU; SSC; SE; SH; SI; SS | 1 | lmrB |
| SAG; SAR; BS; SC; SCH; SF; SHY; MC; SA; SAU; SE; SH; SS | 1 | evgS |
| SAG; SAR; BS; SC; SCH; SF; SHY; SK; MC; SA; SAU; SE; SS | 1 | lmrD |
| SAG; SAR; BS; SC; SCH; SHY; SK; SA; SAU; SSC; SH; SI; SS | 1 | Borreliella burgdorferi murA with mutation conferring resistance to fosfomycin |
| SAG; SAR; SC; SCH; SK; MC; SA; SAU; SSC; SE; SH; SI; SS | 1 | PC1 beta-lactamase (blaZ) |
| SAG; SAR; BS; SC; SCH; SF; SHY; SK; MC; SA; SE; SH; SI | 1 | Staphylococcus aureus murA with mutation conferring resistance to fosfomycin |
| SAG; SAR; BS; SC; SCH; SF; SHY; SK; MC; SA; SSC; SI; SS | 1 | tetA(60) |
| SAG; SAR; SC; SCH; SHY; SK; MC; SA; SSC; SE; SH; SI; SS | 1 | Acinetobacter baumannii AbaF |
| SAG; SAR; BS; SCH; SF; SHY; MC; SAU; SSC; SE; SH; SI; SS | 1 | vanHM |
| SAG; SC; SCH; SF; SHY; SK; MC; SAU; SSC; SE; SH; SI; SS | 1 | vatE |
| SAR; BS; SC; SCH; SHY; SK; SA; SAU; SSC; SE; SH; SI; SS | 1 | norA |
| BS; SC; SCH; SF; SHY; SK; MC; SA; SSC; SE; SH; SI; SS | 1 | marA |
| SAG; SAR; BS; SCH; SF; SHY; SA; SAU; SSC; SE; SH; SI | 1 | vanHB |
| SAG; SAR; SC; SCH; SK; MC; SA; SAU; SE; SH; SI; SS | 2 | mecR1; mecI |
| SAG; SAR; BS; SC; SCH; SF; SHY; SK; SA; SSC; SE; SS | 1 | Staphylococcus aureus norA |
| SAG; SAR; BS; SC; SCH; SHY; SK; MC; SA; SSC; SI; SS | 1 | PEDO-2 |
| SAG; SAR; BS; SC; SCH; SF; SHY; SK; MC; SSC; SE; SH | 1 | vanRM |
| SAG; BS; SC; SCH; SHY; SK; SA; SAU; SE; SH; SI; SS | 1 | MuxC |
| SAG; BS; SC; SCH; SF; SHY; SK; SA; SSC; SE; SH; SS | 1 | ramA |
| SAG; BS; SCH; SF; SHY; SK; MC; SSC; SE; SH; SI; SS | 1 | patB |
| SAR; SC; SCH; SK; MC; SA; SAU; SSC; SE; SH; SI; SS | 1 | salA |
| SAG; SAR; BS; SC; SCH; SHY; SK; MC; SAU; SE; SS | 1 | lmrC |
| SAG; SC; SCH; SF; SHY; SK; MC; SA; SSC; SE; SS | 1 | farA |
| SAG; SAR; BS; SCH; SK; MC; SA; SAU; SE; SH | 1 | Bifidobacterium ileS conferring resistance to mupirocin |
| SAG; SAR; BS; SCH; SHY; SK; SE; SH; SI; SS | 1 | vanE |
| SAG; SAR; BS; SCH; SF; SHY; MC; SSC; SI; SS | 1 | LRA-8 |
| SAG; BS; SC; SCH; SF; SHY; SA; SSC; SI; SS | 1 | AAC(6')-Ia |
| SAG; BS; SHY; MC; SA; SSC; SE; SH; SI; SS | 1 | Escherichia coli ampC1 beta-lactamase |
| SAG; BS; SCH; SHY; SK; MC; SAU; SSC; SE; SI | 1 | vmlR |
| SAG; SC; SCH; SHY; MC; SAU; SSC; SE; SH; SS | 1 | Mycobacterium tuberculosis rpoB mutants conferring resistance to rifampicin |
| SAR; BS; SC; SK; MC; SA; SAU; SE; SH; SS | 1 | lmrP |
| SAR; BS; SC; SCH; SK; MC; SA; SSC; SH; SS | 1 | mtrR |
| SAR; BS; SC; SHY; SK; MC; SA; SE; SH; SI | 1 | blt |
| SAR; BS; SC; SHY; SK; SA; SE; SH; SI; SS | 1 | nalD |
| SAR; SC; SCH; MC; SA; SSC; SE; SH; SI; SS | 1 | tetU |
| SAR; BS; SC; SCH; SK; MC; SSC; SH; SI; SS | 1 | abeS |
| BS; SCH; SHY; MC; SA; SAU; SSC; SE; SH; SI | 1 | SAT-4 |
| BS; SCH; SF; SHY; MC; SAU; SSC; SH; SI; SS | 1 | CRP |
| SAG; SAR; BS; SC; SCH; SHY; SSC; SI; SS | 1 | adeN |
| SAG; SAR; SC; SCH; SHY; MC; SSC; SE; SS | 1 | Escherichia coli soxS with mutation conferring antibiotic resistance |
| SAG; BS; SCH; SHY; MC; SA; SSC; SE; SI | 1 | Staphylococcus aureus GlpT with mutation conferring resistance to fosfomycin |
| SAG; BS; SF; SHY; MC; SA; SE; SH; SS | 1 | kdpE |
| SAG; BS; SC; SCH; SHY; SK; MC; SE; SH | 1 | tet(44) |
| SAR; BS; SC; SF; MC; SA; SAU; SI; SS | 1 | Mycobacterium tuberculosis kasA mutant conferring resistance to isoniazid |
| SAR; SC; SCH; SHY; MC; SA; SAU; SE; SS | 1 | tet(49) |
| SAR; SC; SF; SK; SA; SAU; SE; SH; SS | 1 | GOB-18 |
| SAR; BS; SC; SCH; SHY; SK; SA; SSC; SS | 1 | vanHD |
| SAR; SC; SCH; SHY; SA; SSC; SE; SI; SS | 1 | cfrC |
| BS; SCH; SF; SHY; MC; SA; SE; SH; SS | 1 | vanSM |
| SAG; SAR; BS; SHY; MC; SA; SAU; SE | 1 | Escherichia coli acrR with mutation conferring multidrug antibiotic resistance |
| SAG; SAR; BS; SC; SCH; SA; SH; SS | 1 | vanSG |
| SAG; SAR; BS; SC; SK; SA; SE; SS | 1 | AcrS |
| SAG; SAR; BS; SF; SHY; MC; SA; SH | 1 | sta |
| SAG; SAR; SC; SHY; MC; SA; SH; SS | 1 | AAC(6')-Ie-APH(2'')-Ia |
| SAG; SAR; BS; SCH; SHY; MC; SAU; SSC | 1 | vanUG |
| SAG; SAR; BS; SCH; SHY; MC; SSC; SI | 1 | vanSF |
| SAG; SAR; BS; SC; SF; SK; SE; SS | 1 | pmrA |
| SAG; SCH; SK; SA; SSC; SE; SI; SS | 1 | tetA(46) |
| SAR; SC; SK; SA; SAU; SE; SH; SS | 1 | Mycobacterium tuberculosis inhA mutations conferring resistance to isoniazid |
| SAR; BS; SC; MC; SA; SE; SH; SS | 1 | msrA |
| SAR; SC; SCH; SA; SSC; SE; SH; SS | 1 | mphC |
| SAR; SF; SHY; SK; SA; SE; SI; SS | 1 | rpoB2 |
| SAR; BS; SCH; SF; SAU; SSC; SE; SS | 1 | otr(B) |
| SAR; BS; SC; SHY; MC; SAU; SH; SI | 1 | vanYM |
| SC; SCH; SK; MC; SA; SAU; SH; SS | 1 | MSI-1 |
| BS; SHY; SA; SSC; SE; SH; SI; SS | 1 | mepR |
| SCH; SHY; SK; MC; SA; SSC; SI; SS | 1 | tetX |
| SAG; SAR; BS; SC; SCH; SHY; SA | 1 | mdtG |
| SAG; SAR; BS; SCH; SHY; SA; SSC | 1 | patA |
| SAG; SAR; BS; SC; SCH; SHY; SH | 1 | LRA-2 |
| SAG; SAR; BS; SCH; SHY; SSC; SH | 1 | adeR |
| SAG; BS; SCH; SHY; SSC; SE; SI | 1 | LlmA 23S ribosomal RNA methyltransferase |
| SAG; BS; SCH; SHY; MC; SSC; SH | 1 | mef(B) |
| SAG; BS; SCH; SHY; SSC; SI; SS | 1 | Acinetobacter baumannii AmvA |
| SAR; BS; SCH; SF; MC; SA; SSC | 1 | tet36 |
| SAR; SC; SCH; SK; SA; SSC; SS | 1 | tlrC |
| SAR; SHY; SK; MC; SA; SE; SI | 1 | vatH |
| SAR; BS; SC; SCH; SK; MC; SS | 1 | aadK |
| SAR; BS; SC; SK; MC; SI; SS | 1 | ugd |
| SAR; SC; SCH; SK; SSC; SE; SS | 1 | FosD |
| BS; CS; SCH; SA; SSC; SE; SH | 1 | mecA |
| BS; SK; SA; SSC; SE; SH; SS | 1 | emrY |
| SAG; SAR; BS; SHY; SA; SS | 1 | ANT(6)-Ia |
| SAG; SAR; SCH; SHY; MC; SSC | 1 | SAT-3 |
| SAG; SAR; SF; SHY; MC; SS | 1 | tetB(46) |
| SAG; SCH; SF; SHY; SK; SA | 1 | tet(38) |
| SAG; SCH; SF; SHY; SAU; SI | 1 | Staphylococcus aureus fusE with mutation conferring resistance to fusidic acid |
| SAG; BS; SCH; SF; SHY; MC | 1 | cpxA |
| SAG; SCH; MC; SSC; SE; SH | 1 | vanSE |
| SAR; BS; SC; SA; SE; SH | 1 | cmeR |
| SAR; BS; SA; SE; SH; SS | 1 | mepA |
| SAR; SC; SA; SSC; SE; SH | 1 | tet(K) |
| SAR; BS; SAU; SE; SH; SS | 1 | Bacillus subtilis pgsA with mutation conferring resistance to daptomycin |
| SAR; SC; SAU; SSC; SH; SS | 1 | gadW |
| SAR; BS; SC; SSC; SI; SS | 1 | nalC |
| SAR; SC; SCH; SF; SSC; SS | 1 | SAT-2 |
| SAR; SCH; SK; MC; SSC; SE | 1 | tet(48) |
| SAR; SCH; SK; MC; SSC; SS | 1 | AAC(6')-34 |
| SAR; SC; SHY; SK; SE; SI | 1 | vanWG |
| BS; SF; MC; SA; SAU; SE | 1 | vanRE |
| SCH; SHY; MC; SA; SSC; SI | 1 | Agrobacterium fabrum chloramphenicol acetyltransferase |
| SC; SF; SA; SE; SI; SS | 1 | vgaB |
| BS; SF; SK; SAU; SH; SS | 1 | vanYB |
| BS; SCH; MC; SE; SH; SS | 1 | tet(45) |
| BS; SCH; SF; SHY; SSC; SI | 1 | pgpB |
| BS; SF; MC; SE; SH; SS | 1 | cfrA |
| BS; SHY; SE; SH; SI; SS | 1 | emrK |
| SHY; SK; MC; SH; SI; SS | 1 | msrC |
| SHY; SK; MC; SSC; SI; SS | 1 | vanC |
| SAG; SAR; SK; MC; SA | 1 | catB3 |
| SAG; SC; SHY; SK; SAU | 1 | clbA |
| SAG; SCH; SF; SHY; SE | 1 | srmB |
| SAG; SHY; SK; MC; SH | 1 | tet(L) |
| SAR; MC; SAU; SE; SI | 1 | lnuA |
| SAR; BS; SCH; SHY; SSC | 1 | vanSA |
| SAR; SC; SCH; SSC; SE | 1 | Tet(X4) |
| SAR; SCH; SK; MC; SSC | 1 | emeA |
| SAR; SC; SE; SH; SS | 2 | AAC(6')-IIa ErmC |
| SAR; SC; MC; SE; SS | 1 | vatF |
| BS; SC; SA; SE; SH | 1 | qacA |
| BS; MC; SA; SE; SH | 1 | Mycobacterium tuberculosis rpsL mutations conferring resistance to Streptomycin |
| BS; SA; SSC; SE; SS | 1 | MexR |
| BS; SF; SA; SH; SS | 1 | vga(E) Staphylococcus cohnii |
| BS; SF; SK; SA; SSC | 1 | mefC |
| SCH; SF; MC; SA; SSC | 1 | mdtB |
| SC; SAU; SSC; SE; SS | 1 | lsaC |
| BS; SCH; MC; SSC; SH | 1 | Mycobacterium tuberculosis variant bovis ndh with mutation conferring resistance to isoniazid |
| BS; SC; SK; SE; SS | 2 | Klebsiella pneumoniae KpnH oleI |
| BS; SC; SSC; SE; SS | 1 | vanHF |
| BS; SC; MC; SH; SS | 1 | facT |
| BS; SF; MC; SE; SS | 1 | carA |
| BS; SF; SK; SSC; SH | 1 | aacA43 |
| SAG; BS; SC; SS | 1 | pp-flo |
| SAG; BS; SH; SI | 1 | apmA |
| SAG; SHY; SI; SS | 1 | FosB6 |
| SAR; BS; SA; SH | 1 | dfrG |
| SAR; BS; MC; SA | 1 | efrA |
| SAR; SC; SA; SS | 1 | IMP-31 |
| SAR; BS; SCH; SSC | 1 | EdeQ |
| SAR; BS; SF; SE | 1 | vanSC |
| SAR; SC; SCH; SE | 1 | Staphylococcus aureus gyrB conferring resistance to aminocoumarin |
| SAR; SC; SHY; SI | 1 | GOB-1 |
| SAR; SC; SK; SS | 1 | gadX |
| SAR; SHY; SE; SI | 1 | Tet(47) |
| SAR; SK; SE; SS | 1 | Tet(X3) |
| BS; SA; SE; SH | 1 | CPS-1 |
| SC; SA; SE; SS | 1 | ANT(4')-Ib |
| SC; SA; SH; SS | 1 | AAC(6')-Iaj |
| SC; MC; SA; SI | 1 | aadA9 |
| MC; SA; SE; SH | 1 | APH(3')-IIIa |
| BS; SC; SCH; SS | 1 | RlmA(II) |
| BS; SCH; SK; SSC | 1 | Escherichia coli ampH beta-lactamase |
| BS; SCH; MC; SSC | 1 | tmrB |
| BS; SC; SE; SH | 1 | tetR |
| BS; SC; SF; SS | 1 | vanN |
| BS; SC; MC; SH | 1 | Pseudomonas aeruginosa soxR |
| BS; SK; SE; SS | 1 | BJP-1 |
| BS; SK; SSC; SE | 1 | MexL |
| BS; SSC; SH; SS | 1 | efpA |
| BS; SHY; SSC; SI | 1 | AAC(3)-Ic |
| BS; SK; SSC; SS | 1 | AAC(6')-Im |
| SCH; SSC; SE; SH | 1 | fusB |
| SCH; SK; MC; SSC | 1 | AAC(6')-Iad |
| SK; MC; SE; SH | 1 | vanYA |
| SAG; MC; SA | 1 | vgaC |
| SAG; BS; SE | 1 | tet(43) |
| SAG; SC; SS | 1 | hp1181 |
| SAG; SHY; SS | 1 | Enterococcus faecium EF-Tu mutants conferring resistance to GE2270A |
| SAR; SA; SE | 1 | vatA |
| SAR; SF; SA | 1 | SMB-1 |
| SAR; SF; SAU | 1 | AAC(6')-Iw |
| SAR; BS; SC | 3 | Escherichia coli emrE; FosB5; arr-1 |
| SAR; SCH; SK | 1 | AAC(6')-I30 |
| SAR; SC; SK | 1 | FusF |
| SAR; SC; SS | 2 | AAC(6')-Ip; Enterococcus faecium chloramphenicol acetyltransferase |
| SAR; SK; SH | 1 | catB11 |
| SAR; MC; SSC | 1 | efmA |
| BS; SA; SE | 1 | Staphylococcus aureus parC conferring resistance to fluoroquinolone |
| BS; SK; SA | 1 | mel |
| SC; SA; SSC | 1 | Chlamydia trachomatis intrinsic murA conferring resistance to fosfomycin |
| SC; SA; SI | 1 | ErmA |
| MC; SA; SE | 1 | aad(6) |
| SHY; MC; SA | 1 | catB2 |
| BS; SAU; SS | 1 | Rhodococcus fascians cmr |
| BS; SAU; SSC | 1 | Klebsiella pneumoniae acrR with mutation conferring multidrug antibiotic resistance |
| SAU; SSC; SI | 1 | mefE |
| BS; SCH; SF | 1 | vatC |
| BS; SCH; SSC | 5 | QnrS2; tet(J); LRA-3; vanSL; smeR |
| BS; SK; SE | 3 | Escherichia coli fabG mutations conferring resistance to triclosan; Rm3; adeS |
| BS; MC; SE | 1 | smeS |
| BS; SF; MC | 1 | rosB |
| BS; MC; SH | 1 | Enterococcus faecium liaS mutant conferring daptomycin resistance |
| BS; SSC; SH | 1 | tcr3 |
| BS; SHY; MC | 1 | vanO |
| BS; SI; SS | 1 | basS |
| SCH; SF; SSC | 1 | ANT(6)-Ib |
| SCH; MC; SSC | 1 | Pseudomonas aeruginosa emrE |
| SC; SE; SI | 1 | vgaA |
| MC; SE; SH | 1 | PEDO-1 |
| MC; SSC; SE | 1 | Escherichia coli gyrA conferring resistance to fluoroquinolones |
| SF; SHY; SI | 1 | Mycobacterium tuberculosis gidB mutation conferring resistance to streptomycin |
| SHY; SI; SS | 1 | tet(Z) |
| SAG; SA | 1 | MexZ |
| SAG; SF | 2 | AAC(6')-Ix tet(54) |
| SAG; SH | 1 | tet(55) |
| SAG; SHY | 1 | AAC(6')-Ir |
| SAG; SK | 1 | pexA |
| SAG; MC | 2 | fusC; mdtC |
| SAR; SA | 1 | IMP-35 |
| SAR; BS | 4 | vanSN; QepA4; CAU-1; catB9 |
| SAR; SK | 2 | Salmonella serovars soxS with mutation conferring antibiotic resistance; qacB |
| BS; SA | 4 | AAC(6')-33; tet(56); Staphylococcus aureus rpoB mutants conferring resistance to rifampicin; vanD |
| SC; SA | 1 | lsaA |
| SA; SE | 2 | bcrB; Staphylococcus aureus ileS with mutation conferring resistance to mupirocin |
| SA; SH | 2 | Staphylococcus aureus gyrA conferring resistance to fluoroquinolones; rpsJ |
| MC; SA | 2 | tetS; AAC(6')-Iai |
| SA; SS | 1 | AAC(6')-Ik |
| SC; SAU | 1 | tet(V) |
| MC; SAU | 1 | vanRD |
| BS; SCH | 4 | qacH; tet(30); TEM-116; AAC(6')-Iy |
| BS; SC | 6 | FosA7; Enterococcus faecium cls conferring resistance to daptomycin; AIM-1; vanYG1; iri; bcrC |
| BS; SE | 4 | lrfA; farB; tet(59); FosB4 |
| BS; SF | 2 | tetA(P); Escherichia coli EF-Tu mutants conferring resistance to Enacyloxin; Iia |
| BS; SH | 2 | AAC(6')-Iae; FosB1 |
| BS; SK | 1 | Escherichia coli marR mutant conferring antibiotic resistance |
| BS; MC | 4 | Mycolicibacterium smegmatis ndh with mutation conferring resistance to isoniazid; vanRB; vanRC; Rhodobacter sphaeroides ampC beta-lactamase |
| BS; SS | 2 | VatI; vanRG |
| BS; SSC | 1 | tet(Y) |
| BS; SI | 2 | tet(50); LRA-7 |
| SCH; SK | 1 | QepA2 |
| SCH; SSC | 11 | Mycoplasma genitalium parC mutations confers resistance to Moxifloxacin; Enterococcus faecium liaF mutant conferring daptomycin resistance; AAC(3)-Id; AAC(6')-Ib-SK; vanXYE; AAC(6')-Ian; AAC(3)-Ib/AAC(6')-Ib''; Staphylococcus aureus mprF with mutation conferring resistance to daptomycin; aadA12; AAC(6')-Ib11; tet(53) |
| SC; SH | 1 | Ureaplasma urealyticum parC conferring resistance to fluoroquinolone |
| SC; SS | 1 | vatD |
| SE; SH | 1 | ESP-1 |
| SK; SE | 1 | tet(42) |
| SF; MC | 1 | YojI |
| SH; SS | 2 | Staphylococcus intermedius chloramphenicol acetyltransferase; tet(52) |
| SHY; SI | 2 | Staphylococcus aureus cls conferring resistance to daptomycin; ACT-18 |
| SK; MC | 1 | baeR |
| SK; SS | 1 | vanXYL |
| MC; SS | 2 | ErmB; dfrK |
| SAG | 2 | tet(H); tet(41) |
| SAR | 9 | catA8; FosB3; emrA; gimA; spd; Corynebacterium striatum tetA; FosA6; mdtH; otrC |
| SA | 6 | OXA-192; Pseudomonas aeruginosa catB6; AAC(6')-Ib3; tetM; Clostridioides difficile rpoB with mutation conferring resistance to rifampicin; Staphylococcus aureus FosB |
| SAU | 4 | vanA; CMY-51; APH(9)-Ia; vanRO |
| BS | 48 | MdtK; tet(33); AAC(6')-Ic; Mycobacterium tuberculosis thyA with mutation conferring resistance to para-aminosalicylic acid; D-Ala-D-Ala ligase; mphK; tet(39); Bla1; LRA-19; tet(40); CMY-113; erm(45); rphA; OXA-204; EreA2; Neisseria meningititis PBP2 conferring resistance to beta-lactam; erm(49); tet(A); vgbA; macA; rosA; ACC-2; IMP-22; oqxA; FosB; erm(30); vanWI; AAC(6')-Il; tet(D); cmlA4; bmr; Mycoplasma hominis parC conferring resistance to fluoroquinolone; SRT-2; PmpM; Erm(31); BcI; abeM; AAC(3)-IXa; OXA-247; MIR-14; ErmE; IMP-20; blaR1; mfpA; AAC(6')-Isa; tet(31); vanZF; cmrA |
| SCH | 5 | DHA-15; DHA-2; AAC(6')-It; AAC(6')-Iv; ermT |
| SC | 10 | blaI; ErmT; smeD; Erm(37); cmeB; arr-4; Enterococcus faecalis chloramphenicol acetyltransferase; arr-5; Erm(43); Klebsiella pneumoniae ramR mutants |
| SE | 8 | AAC(6')-Is; Escherichia coli EF-Tu mutants conferring resistance to Pulvomycin; ADC-19; vanTC; ACC-3; Escherichia coli ampC beta-lactamase; FosA; vanXYG |
| SF | 4 | Erm(47); MCR-9.1; cmlB1; Streptomyces lividans cmlR |
| SH | 3 | BEL-2; vgaALC; BEL-1; |
| SHY | 2 | catB10; AAC(6')-Iu |
| SK | 3 | sdiA; VEB-3; MuxA |
| MC | 19 | LRA-12; mphM; vanSB; npmA; THIN-B; mecB; APH(2'')-IIIa; cpaA; dfrA12; vanYF; L1 beta-lactamase; Klebsiella pneumoniae OmpK37; lnuG; emtA; Staphylococcus aureus walK with mutation conferring resistance to daptomycin; lin; Pseudomonas aeruginosa CpxR; APH(2'')-IVa; msrE |
| SS | 13 | vanRA; tet(B); ANT(3'')-Iia; Erm(44)v; mecC; mdtM; AAC(6')-30/AAC(6')-Ib' fusion protein; FosA2; AAC(6')-Ig; QnrB29; QnrC; AAC(6')-Ij; fusD |
| SSC | 6 | vanXM; Escherichia coli soxR with mutation conferring antibiotic resistance; lnuE; ANT(2'')-Ia; Streptomyces rishiriensis parY mutant conferring resistance to aminocoumarin; AAC(6')-Ib' |
| SI | 4 | LnuP; ACT-22; ACT-12; CMY-26 |

^*^**SA**: *S. aureus*; **SE**: *S. epidermidis*; **SAR***: S. arlettae*; **SAU**: *S. auricularis*; **SI**: *S. simulans*; **SSC**: *S. sciuri*; **SCH**: *S. chromogenes*; **SC**: *S. cohnii*; **SHY**: *S. hyicus*; **SS**: *S. saprophyticus*; **SAG**: *S. agnetis*; **SK**: *S. kloosii*; **SH**: *S. haemolyticus***; SF**: *S. felis*; **MC**: *Macrococcus caseolyticus*; **BS**: *Bacillus subtilis*.
