# Supplementary material for: Staphylococcus arlettae Genomics: Novel Insights on Candidate Antibiotic Resistance and Virulence Genes in an Emerging Opportunistic Pathogen
Source: Microorganisms. 2019 Nov 19;7(11):580. doi: 10.3390/microorganisms7110580 (PMC6920755; doi:10.3390/microorganisms7110580)
Supplement: Supplementary file 1 [file microorganisms-07-00580-s001.zip › Table S5.docx]

**Table S5**. Virulence elements of SAR Bari draft genome^*^

| **N** | **Synonym** | **Original sequence name** | **Length(aa)** | **VF** | | **AR** | | **T3SE** | | **T4SE** | | **T6SE** | | **T7SE** | | **Prophage** | | **ICE** | | **T3SS** | | **T4SS** | | **T6SS** | | **T7SS** | | **Integron** | | **IS** | | **PAI** | | **ARI** | |
| --- | --- | --- | --- | --- | --- | --- | --- | --- | --- | --- | --- | --- | --- | --- | --- | --- | --- | --- | --- | --- | --- | --- | --- | --- | --- | --- | --- | --- | --- | --- | --- | --- | --- | --- | --- |
|  |  |  |  | **Hit** | **Ha-value** | **Hit** | **Ha-value** | **Hit** | **Ha-value** | **Hit** | **Ha-value** | **Hit** | **Ha-value** | **Hit** | **Ha-value** | **Hit** | **Ha-value** | **Hit** | **Ha-value** | **Hit** | **Ha-value** | **Hit** | **Ha-value** | **Hit** | **Ha-value** | **Hit** | **Ha-value** | **Hit** | **Ha-value** | **Hit** | **Ha-value** | **Hit** | **Ha-value** | **Hit** | **Ha-value** |
| **1** | P0006 | PROKKA_00006 | 263 |  |  |  |  |  |  |  |  |  |  |  |  | Prophage_61806026 | 0.278 |  |  |  |  |  |  |  |  |  |  |  |  |  |  |  |  |  |  |
| **2** | P0017 | PROKKA_00017 | 220 | VFG0596 | 0.427 |  |  |  |  |  |  |  |  |  |  |  |  |  |  |  |  |  |  |  |  |  |  |  |  |  |  |  |  |  |  |
| **3** | P0027 | PROKKA_00027 | 172 |  |  | AR_CAE53424 | 0.343 |  |  |  |  |  |  |  |  |  |  |  |  |  |  |  |  |  |  |  |  |  |  |  |  |  |  |  |  |
| **4** | P0028 | PROKKA_00028 | 318 |  |  |  |  |  |  |  |  |  |  |  |  | Prophage_148747783 | 0.459 |  |  |  |  |  |  |  |  |  |  |  |  |  |  |  |  |  |  |
| **5** | P0036 | PROKKA_00036 | 211 | VFG0921 | 0.246 |  |  |  |  |  |  |  |  |  |  |  |  |  |  |  |  |  |  |  |  |  |  |  |  |  |  |  |  |  |  |
| **6** | P0047 | PROKKA_00047 | 430 | VFG1668 | 0.241 |  |  |  |  |  |  |  |  |  |  |  |  |  |  |  |  |  |  |  |  |  |  |  |  |  |  | PAI_CAD66193 | 0.241 |  |  |
| **7** | P0059 | PROKKA_00059 | 388 |  |  |  |  |  |  |  |  |  |  |  |  |  |  |  |  |  |  |  |  |  |  |  |  |  |  |  |  | PAI_ABW69089 | 0.284 |  |  |
| **8** | P0064 | PROKKA_00064 | 90 |  |  |  |  |  |  |  |  |  |  |  |  | Prophage_156564019 | 0.711 |  |  |  |  |  |  |  |  |  |  |  |  |  |  |  |  |  |  |
| **9** | P0077 | PROKKA_00077 | 241 |  |  | AR_ZP_02422325 | 0.398 |  |  |  |  |  |  |  |  |  |  |  |  |  |  |  |  |  |  |  |  |  |  |  |  |  |  |  |  |
| **10** | P0082 | PROKKA_00082 | 295 |  |  |  |  |  |  |  |  |  |  |  |  |  |  |  |  |  |  |  |  |  |  |  |  |  |  |  |  | PAI_AAN64203 | 0.318 | ARI_409973533 | 0.312 |
| **11** | P0083 | PROKKA_00083 | 148 | VFG0478 | 0.324 |  |  |  |  |  |  |  |  |  |  |  |  |  |  |  |  |  |  |  |  |  |  |  |  |  |  |  |  |  |  |
| **12** | P0087 | PROKKA_00087 | 272 | VFG1384 | 0.246 |  |  |  |  |  |  |  |  |  |  |  |  |  |  |  |  |  |  |  |  |  |  |  |  |  |  |  |  |  |  |
| **13** | P0089 | PROKKA_00089 | 494 |  |  |  |  |  |  |  |  |  |  |  |  | Prophage_113200631 | 0.340 |  |  |  |  |  |  |  |  |  |  |  |  |  |  |  |  |  |  |
| **14** | P0099 | PROKKA_00099 | 473 |  |  |  |  |  |  |  |  |  |  |  |  |  |  |  |  |  |  |  |  |  |  |  |  |  |  |  |  |  |  | ARI_14021018 | 0.302 |
| **15** | P0114 | PROKKA_00114 | 276 | VFG2158 | 0.246 |  |  |  |  |  |  |  |  |  |  |  |  |  |  |  |  |  |  |  |  |  |  |  |  |  |  |  |  |  |  |
| **16** | P0115 | PROKKA_00115 | 128 |  |  |  |  |  |  |  |  |  |  |  |  |  |  |  |  |  |  |  |  |  |  |  |  |  |  |  |  |  |  | ARI_28465866 | 0.266 |
| **17** | P0119 | PROKKA_00119 | 170 |  |  |  |  | T3SE_Q989M4 | 0.259 |  |  |  |  |  |  |  |  |  |  |  |  |  |  |  |  |  |  |  |  |  |  |  |  |  |  |
| **18** | P0126 | PROKKA_00126 | 324 | VFG1876 | 0.321 |  |  |  |  |  |  |  |  |  |  |  |  |  |  |  |  |  |  |  |  |  |  |  |  |  |  | PAI_CAE85233 | 0.309 |  |  |
| **19** | P0127 | PROKKA_00127 | 205 |  |  | CBMAR_A4FI79 | 0.254 |  |  |  |  |  |  |  |  |  |  |  |  |  |  |  |  |  |  |  |  |  |  |  |  |  |  |  |  |
| **20** | P0135 | PROKKA_00135 | 199 | VFG1867 | 0.472 |  |  |  |  |  |  |  |  |  |  |  |  |  |  |  |  |  |  |  |  |  |  |  |  |  |  |  |  |  |  |
| **21** | P0136 | PROKKA_00136 | 137 | VFG0478 | 0.277 |  |  |  |  |  |  |  |  |  |  |  |  |  |  |  |  |  |  |  |  |  |  |  |  |  |  |  |  |  |  |
| **22** | P0138 | PROKKA_00138 | 258 |  |  |  |  |  |  |  |  |  |  |  |  |  |  |  |  |  |  |  |  |  |  |  |  |  |  |  |  | PAI_CAA21355 | 0.256 | ARI_15808722 | 0.271 |
| **23** | P0139 | PROKKA_00139 | 296 |  |  |  |  | T3SE_O84630 | 0.267 |  |  |  |  |  |  |  |  |  |  |  |  |  |  |  |  |  |  |  |  |  |  |  |  |  |  |
| **24** | P0143 | PROKKA_00143 | 368 | VFG1405 | 0.522 |  |  |  |  |  |  |  |  |  |  |  |  |  |  |  |  |  |  |  |  |  |  |  |  |  |  |  |  |  |  |
| **25** | P0153 | PROKKA_00153 | 315 |  |  |  |  |  |  |  |  |  |  |  |  | Prophage_19343479 | 0.225 |  |  |  |  |  |  |  |  |  |  |  |  |  |  | PAI_CAA21345 | 0.317 |  |  |
| **26** | P0169 | PROKKA_00169 | 137 |  |  |  |  |  |  |  |  |  |  |  |  | Prophage_115334647 | 0.314 |  |  |  |  |  |  |  |  |  |  |  |  |  |  |  |  |  |  |
| **27** | P0171 | PROKKA_00171 | 153 |  |  |  |  |  |  |  |  |  |  |  |  | Prophage_156564196 | 0.444 |  |  |  |  |  |  |  |  |  |  |  |  |  |  |  |  |  |  |
| **28** | P0200 | PROKKA_00200 | 380 |  |  |  |  |  |  |  |  |  |  |  |  |  |  |  |  |  |  |  |  |  |  |  |  |  |  |  |  | PAI_ABP49156 | 0.376 |  |  |
| **29** | P0201 | PROKKA_00201 | 512 |  |  | AR_CAA45050 | 0.255 |  |  |  |  |  |  |  |  |  |  |  |  |  |  |  |  |  |  |  |  |  |  |  |  |  |  |  |  |
| **30** | P0208 | PROKKA_00208 | 590 |  |  |  |  |  |  |  |  |  |  |  |  |  |  |  |  |  |  |  |  |  |  |  |  |  |  |  |  |  |  | ARI_484356532 | 0.519 |
| **31** | P0212 | PROKKA_00212 | 729 | VFG1826 | 0.405 |  |  |  |  |  |  |  |  |  |  |  |  |  |  |  |  |  |  |  |  |  |  |  |  |  |  |  |  |  |  |
| **32** | P0218 | PROKKA_00218 | 341 |  |  |  |  |  |  |  |  |  |  |  |  |  |  |  |  |  |  |  |  |  |  |  |  |  |  |  |  | PAI_AAF71483 | 0.468 |  |  |
| **33** | P0243 | PROKKA_00243 | 398 |  |  |  |  |  |  |  |  |  |  |  |  |  |  |  |  |  |  |  |  |  |  |  |  |  |  |  |  | PAI_CAA21341 | 0.342 | ARI_12698391 | 0.271 |
| **34** | P0245 | PROKKA_00245 | 465 |  |  |  |  |  |  |  |  |  |  |  |  | Prophage_38707949 | 0.271 |  |  |  |  |  |  |  |  |  |  |  |  |  |  | PAI_CAA21369 | 0.206 |  |  |
| **35** | P0249 | PROKKA_00249 | 128 | VFG1416 | 0.437 |  |  |  |  |  |  |  |  |  |  |  |  |  |  |  |  |  |  |  |  |  |  |  |  |  |  |  |  |  |  |
| **36** | P0250 | PROKKA_00250 | 284 | VFG1417 | 0.310 |  |  |  |  |  |  |  |  |  |  |  |  |  |  |  |  |  |  |  |  |  |  |  |  |  |  |  |  |  |  |
| **37** | P0255 | PROKKA_00255 | 496 | VFG0082 | 0.403 |  |  |  |  |  |  |  |  |  |  |  |  |  |  |  |  |  |  |  |  |  |  |  |  |  |  | PAI_AAK20776 | 0.401 |  |  |
| **38** | P0264 | PROKKA_00264 | 459 |  |  |  |  |  |  |  |  |  |  |  |  |  |  |  |  |  |  |  |  |  |  |  |  |  |  |  |  | PAI_AAO17183 | 0.477 |  |  |
| **39** | P0265 | PROKKA_00265 | 437 |  |  |  |  |  |  |  |  |  |  |  |  |  |  |  |  |  |  |  |  |  |  |  |  |  |  |  |  | PAI_CAA21341 | 0.304 |  |  |
| **40** | P0280 | PROKKA_00280 | 399 |  |  |  |  |  |  |  |  |  |  |  |  |  |  |  |  |  |  |  |  |  |  |  |  |  |  |  |  |  |  | ARI_16579875 | 0.429 |
| **41** | P0287 | PROKKA_00287 | 448 |  |  |  |  |  |  |  |  |  |  |  |  |  |  |  |  |  |  |  |  |  |  |  |  |  |  |  |  | PAI_CAA21364 | 0.299 |  |  |
| **42** | P0289 | PROKKA_00289 | 304 | VFG0483 | 0.355 |  |  |  |  |  |  |  |  |  |  |  |  |  |  |  |  |  |  |  |  |  |  |  |  |  |  | PAI_CAE85173 | 0.329 |  |  |
| **43** | P0292 | PROKKA_00292 | 336 | VFG2197 | 0.305 |  |  |  |  |  |  |  |  |  |  |  |  |  |  |  |  |  |  |  |  |  |  |  |  |  |  |  |  |  |  |
| **44** | P0304 | PROKKA_00304 | 479 |  |  |  |  |  |  |  |  |  |  |  |  |  |  |  |  |  |  |  |  |  |  |  |  |  |  |  |  | PAI_AAO17183 | 0.489 |  |  |
| **45** | P0305 | PROKKA_00305 | 466 | VFG1399 | 0.285 |  |  |  |  |  |  |  |  |  |  |  |  |  |  |  |  |  |  |  |  |  |  |  |  |  |  |  |  |  |  |
| **46** | P0306 | PROKKA_00306 | 255 |  |  |  |  |  |  |  |  |  |  |  |  |  |  |  |  |  |  |  |  |  |  |  |  |  |  |  |  | PAI_AAO17195 | 0.298 | ARI_12698385 | 0.314 |
| **47** | P0314 | PROKKA_00314 | 475 |  |  |  |  |  |  |  |  |  |  |  |  |  |  |  |  |  |  |  |  |  |  |  |  |  |  |  |  | PAI_AAO17183 | 0.381 |  |  |
| **48** | P0318 | PROKKA_00318 | 506 | VFG0082 | 0.585 |  |  |  |  |  |  |  |  |  |  |  |  |  |  |  |  |  |  |  |  |  |  |  |  |  |  | PAI_AAK20776 | 0.585 |  |  |
| **49** | P0321 | PROKKA_00321 | 293 |  |  |  |  |  |  |  |  |  |  |  |  |  |  |  |  |  |  |  |  |  |  |  |  |  |  |  |  | PAI_CAI77370 | 0.248 |  |  |
| **50** | P0327 | PROKKA_00327 | 504 |  |  |  |  |  |  |  |  |  |  |  |  |  |  |  |  |  |  |  |  |  |  |  |  |  |  |  |  | PAI_CAA21394 | 0.270 | ARI_12698386 | 0.287 |
| **51** | P0332 | PROKKA_00332 | 129 | VFG0331 | 0.323 |  |  |  |  |  |  |  |  |  |  | Prophage_61806180 | 0.295 |  |  |  |  |  |  |  |  |  |  |  |  |  |  |  |  |  |  |
| **52** | P0340 | PROKKA_00340 | 222 |  |  | AR_YP_001096294 | 0.279 |  |  |  |  |  |  |  |  |  |  |  |  |  |  |  |  |  |  |  |  |  |  |  |  |  |  |  |  |
| **53** | P0341 | PROKKA_00341 | 215 | VFG1583 | 0.335 |  |  |  |  |  |  |  |  |  |  |  |  |  |  |  |  |  |  |  |  |  |  |  |  |  |  |  |  |  |  |
| **54** | P0343 | PROKKA_00343 | 151 | VFG1583 | 0.265 |  |  |  |  |  |  |  |  |  |  |  |  |  |  |  |  |  |  |  |  |  |  |  |  |  |  |  |  |  |  |
| **55** | P0347 | PROKKA_00347 | 323 |  |  |  |  |  |  |  |  |  |  |  |  | Prophage_9635491 | 0.375 |  |  |  |  |  |  |  |  |  |  |  |  |  |  |  |  |  |  |
| **56** | P0353 | PROKKA_00353 | 251 |  |  |  |  |  |  |  |  |  |  |  |  |  |  |  |  |  |  |  |  |  |  |  |  |  |  |  |  |  |  | ARI_5360802 | 0.498 |
| **57** | P0377 | PROKKA_00377 | 250 |  |  |  |  |  |  |  |  |  |  |  |  |  |  |  |  |  |  |  |  |  |  |  |  |  |  |  |  | PAI_AAO17195 | 0.290 | ARI_12698385 | 0.320 |
| **58** | P0383 | PROKKA_00383 | 242 |  |  |  |  |  |  |  |  |  |  |  |  | Prophage_66395003 | 0.264 |  |  |  |  |  |  |  |  |  |  |  |  |  |  |  |  |  |  |
| **59** | P0389 | PROKKA_00389 | 254 |  |  |  |  |  |  |  |  |  |  |  |  |  |  |  |  |  |  |  |  |  |  |  |  |  |  |  |  | PAI_AAO17195 | 0.302 | ARI_12698385 | 0.283 |
| **60** | P0391 | PROKKA_00391 | 403 |  |  |  |  |  |  |  |  |  |  |  |  | Prophage_66391578 | 0.310 |  |  |  |  |  |  |  |  |  |  |  |  |  |  |  |  |  |  |
| **61** | P0399 | PROKKA_00399 | 479 | VFG0617 | 0.284 |  |  |  |  |  |  |  |  |  |  |  |  |  |  |  |  |  |  |  |  |  |  |  |  |  |  |  |  |  |  |
| **62** | P0401 | PROKKA_00401 | 570 | VFG0940 | 0.258 |  |  |  |  |  |  |  |  |  |  |  |  |  |  |  |  |  |  |  |  |  |  |  |  |  |  |  |  |  |  |
| **63** | P0402 | PROKKA_00402 | 419 |  |  | AR_YP_001005900 | 0.255 |  |  |  |  |  |  |  |  |  |  |  |  |  |  |  |  |  |  |  |  |  |  |  |  |  |  |  |  |
| **64** | P0407 | PROKKA_00407 | 331 | VFG0926 | 0.341 |  |  |  |  |  |  |  |  |  |  |  |  |  |  |  |  |  |  |  |  |  |  |  |  |  |  |  |  | ARI_15808723 | 0.308 |
| **65** | P0408 | PROKKA_00408 | 332 |  |  |  |  |  |  |  |  |  |  |  |  |  |  |  |  |  |  |  |  |  |  |  |  |  |  |  |  | PAI_AAL08452 | 0.292 | ARI_15808723 | 0.292 |
| **66** | P0412 | PROKKA_00412 | 736 | VFG1316 | 0.283 |  |  |  |  |  |  |  |  |  |  |  |  |  |  |  |  |  |  |  |  |  |  |  |  |  |  |  |  |  |  |
| **67** | P0415 | PROKKA_00415 | 280 |  |  | AR_ZP_03837411 | 0.246 |  |  |  |  |  |  |  |  |  |  |  |  |  |  |  |  |  |  |  |  |  |  |  |  |  |  |  |  |
| **68** | P0424 | PROKKA_00424 | 732 |  |  | AR_NP_344871 | 0.303 |  |  |  |  |  |  |  |  |  |  |  |  |  |  |  |  |  |  |  |  |  |  |  |  |  |  |  |  |
| **69** | P0438 | PROKKA_00438 | 171 | VFG2161 | 0.421 |  |  |  |  |  |  |  |  |  |  |  |  |  |  |  |  |  |  |  |  |  |  |  |  |  |  |  |  |  |  |
| **70** | P0447 | PROKKA_00447 | 203 |  |  |  |  |  |  |  |  |  |  |  |  | Prophage_61805915 | 0.246 |  |  |  |  |  |  |  |  |  |  |  |  |  |  |  |  |  |  |
| **71** | P0451 | PROKKA_00451 | 565 | VFG2160 | 0.453 |  |  |  |  |  |  |  |  |  |  |  |  |  |  |  |  |  |  |  |  |  |  |  |  |  |  |  |  |  |  |
| **72** | P0456 | PROKKA_00456 | 231 |  |  |  |  |  |  |  |  |  |  |  |  |  |  |  |  |  |  |  |  |  |  |  |  |  |  |  |  | PAI_CAA21346 | 0.364 |  |  |
| **73** | P0458 | PROKKA_00458 | 310 |  |  | AR_YP_002850217 | 0.287 |  |  |  |  |  |  |  |  |  |  |  |  |  |  |  |  |  |  |  |  |  |  |  |  |  |  |  |  |
| **74** | P0461 | PROKKA_00461 | 248 | VFG2061 | 0.278 |  |  |  |  |  |  |  |  |  |  |  |  |  |  |  |  |  |  |  |  |  |  |  |  |  |  | PAI_ACN89319 | 0.298 |  |  |
| **75** | P0471 | PROKKA_00471 | 681 |  |  |  |  |  |  |  |  |  |  |  |  |  |  |  |  |  |  |  |  |  |  |  |  |  |  |  |  |  |  | ARI_90265357 | 0.307 |
| **76** | P0472 | PROKKA_00472 | 121 | VFG1314 | 0.636 |  |  |  |  |  |  |  |  |  |  |  |  |  |  |  |  |  |  |  |  |  |  |  |  |  |  |  |  |  |  |
| **77** | P0473 | PROKKA_00473 | 347 | VFG1314 | 0.349 |  |  |  |  |  |  |  |  |  |  |  |  |  |  |  |  |  |  |  |  |  |  |  |  |  |  |  |  |  |  |
| **78** | P0476 | PROKKA_00476 | 308 | VFG0362 | 0.263 |  |  |  |  |  |  |  |  |  |  |  |  |  |  |  |  |  |  |  |  |  |  |  |  |  |  | PAI_CAA21391 | 0.263 |  |  |
| **79** | P0477 | PROKKA_00477 | 245 |  |  |  |  |  |  |  |  |  |  |  |  |  |  |  |  |  |  |  |  |  |  |  |  |  |  |  |  | PAI_AAO17195 | 0.344 | ARI_12698385 | 0.363 |
| **80** | P0495 | PROKKA_00495 | 688 |  |  |  |  |  |  |  |  |  |  |  |  |  |  |  |  |  |  |  |  |  |  |  |  |  |  |  |  |  |  | ARI_672940418 | 0.366 |
| **81** | P0497 | PROKKA_00497 | 296 |  |  |  |  |  |  |  |  |  |  |  |  |  |  |  |  |  |  |  |  |  |  |  |  |  |  |  |  |  |  | ARI_409973533 | 0.283 |
| **82** | P0527 | PROKKA_00527 | 234 | VFG0934 | 0.192 |  |  |  |  |  |  |  |  |  |  |  |  |  |  |  |  |  |  |  |  |  |  |  |  |  |  | PAI_AAO17195 | 0.274 |  |  |
| **83** | P0532 | PROKKA_00532 | 350 |  |  |  |  |  |  |  |  |  |  |  |  | Prophage_109302868 | 0.523 |  |  |  |  |  |  |  |  |  |  |  |  |  |  |  |  |  |  |
| **84** | P0538 | PROKKA_00538 | 206 |  |  |  |  |  |  |  |  |  |  |  |  | Prophage_9630246 | 0.277 |  |  |  |  |  |  |  |  |  |  |  |  |  |  |  |  |  |  |
| **85** | P0539 | PROKKA_00539 | 207 |  |  |  |  |  |  |  |  |  |  |  |  | Prophage_22911995 | 0.285 |  |  |  |  |  |  |  |  |  |  |  |  |  |  |  |  |  |  |
| **86** | P0544 | PROKKA_00544 | 869 | VFG0562 | 0.374 |  |  |  |  |  |  |  |  |  |  |  |  |  |  |  |  |  |  |  |  |  |  |  |  |  |  |  |  |  |  |
| **87** | P0557 | PROKKA_00557 | 446 | VFG1399 | 0.361 |  |  |  |  |  |  |  |  |  |  |  |  |  |  |  |  |  |  |  |  |  |  |  |  |  |  |  |  |  |  |
| **88** | P0568 | PROKKA_00568 | 450 | VFG0171 | 0.269 |  |  |  |  |  |  |  |  |  |  |  |  |  |  |  |  |  |  |  |  |  |  |  |  |  |  |  |  |  |  |
| **89** | P0578 | PROKKA_00580 | 329 | VFG2158 | 0.533 |  |  |  |  |  |  |  |  |  |  |  |  |  |  |  |  |  |  |  |  |  |  |  |  |  |  |  |  |  |  |
| **90** | P0585 | PROKKA_00587 | 520 |  |  | AR_AAO38916 | 0.248 |  |  |  |  |  |  |  |  |  |  |  |  |  |  |  |  |  |  |  |  |  |  |  |  |  |  |  |  |
| **91** | P0594 | PROKKA_00596 | 256 | VFG1922 | 0.254 |  |  |  |  |  |  |  |  |  |  |  |  |  |  |  |  |  |  |  |  |  |  |  |  |  |  | PAI_AAO17195 | 0.246 |  |  |
| **92** | P0611 | PROKKA_00613 | 314 | VFG0344 | 0.274 |  |  |  |  |  |  |  |  |  |  |  |  |  |  |  |  |  |  |  |  |  |  |  |  |  |  |  |  |  |  |
| **93** | P0616 | PROKKA_00618 | 414 |  |  |  |  |  |  |  |  |  |  |  |  |  |  |  |  |  |  |  |  |  |  |  |  |  |  |  |  | PAI_CAI36078 | 0.449 |  |  |
| **94** | P0619 | PROKKA_00621 | 864 | VFG0079 | 0.456 |  |  |  |  |  |  |  |  |  |  |  |  |  |  |  |  |  |  | T6SS_108761289_TssH | 0.461 |  |  |  |  |  |  |  |  |  |  |
| **95** | P0620 | PROKKA_00622 | 139 |  |  |  |  |  |  |  |  |  |  |  |  |  |  |  |  |  |  |  |  |  |  |  |  |  |  |  |  |  |  | ARI_229002244 | 0.424 |
| **96** | P0621 | PROKKA_00623 | 136 |  |  |  |  |  |  |  |  |  |  |  |  |  |  |  |  |  |  |  |  |  |  |  |  |  |  |  |  | PAI_ACN89292 | 0.191 | ARI_229002245 | 0.551 |
| **97** | P0626 | PROKKA_00628 | 300 |  |  |  |  |  |  |  |  |  |  |  |  |  |  |  |  |  |  |  |  |  |  |  |  |  |  |  |  | PAI_AAO17178 | 0.317 |  |  |
| **98** | P0630 | PROKKA_00632 | 190 | DBETH_Q73VP2 | 0.277 |  |  |  |  |  |  |  |  |  |  |  |  |  |  |  |  |  |  |  |  |  |  |  |  |  |  |  |  |  |  |
| **99** | P0636 | PROKKA_00638 | 321 |  |  |  |  |  |  |  |  |  |  |  |  | Prophage_66394953 | 0.327 |  |  |  |  |  |  |  |  |  |  |  |  |  |  |  |  |  |  |
| **100** | P0638 | PROKKA_00640 | 396 |  |  |  |  |  |  |  |  |  |  |  |  |  |  |  |  |  |  |  |  |  |  |  |  |  |  |  |  | PAI_CAA21341 | 0.343 |  |  |
| **101** | P0641 | PROKKA_00643 | 198 |  |  |  |  |  |  | T4SE_52842179 | 0.364 |  |  |  |  |  |  |  |  |  |  |  |  |  |  |  |  |  |  |  |  |  |  |  |  |
| **102** | P0659 | PROKKA_00661 | 80 |  |  |  |  |  |  |  |  |  |  |  |  | Prophage_61806099 | 0.375 |  |  |  |  |  |  |  |  |  |  |  |  |  |  |  |  |  |  |
| **103** | P0663 | PROKKA_00665 | 487 | VFG1821 | 0.284 |  |  |  |  |  |  |  |  |  |  |  |  |  |  |  |  |  |  |  |  |  |  |  |  |  |  |  |  |  |  |
| **104** | P0665 | PROKKA_00667 | 320 |  |  | AR_AAD41881 | 0.262 |  |  |  |  |  |  |  |  |  |  |  |  |  |  |  |  |  |  |  |  |  |  |  |  |  |  |  |  |
| **105** | P0694 | PROKKA_00696 | 102 |  |  |  |  |  |  |  |  |  |  |  |  | Prophage_13095675 | 0.284 |  |  |  |  |  |  |  |  |  |  |  |  |  |  |  |  |  |  |
| **106** | P0710 | PROKKA_00712 | 66 |  |  |  |  |  |  |  |  |  |  |  |  | Prophage_13095918 | 0.621 |  |  |  |  |  |  |  |  |  |  |  |  |  |  | PAI_AAO18076 | 0.591 |  |  |
| **107** | P0712 | PROKKA_00714 | 270 | VFG0934 | 0.219 |  |  |  |  |  |  |  |  |  |  |  |  |  |  |  |  |  |  |  |  |  |  |  |  |  |  | PAI_331029102 | 0.300 |  |  |
| **108** | P0731 | PROKKA_00734 | 823 | VFG0079 | 0.683 |  |  |  |  |  |  |  |  |  |  |  |  |  |  |  |  |  |  | T6SS_108761289_TssH | 0.499 |  |  |  |  |  |  |  |  |  |  |
| **109** | P0753 | PROKKA_00756 | 696 |  |  | AR_AAF01499 | 0.261 |  |  |  |  |  |  |  |  |  |  |  |  |  |  |  |  |  |  |  |  |  |  |  |  |  |  |  |  |
| **110** | P0755 | PROKKA_00758 | 391 |  |  |  |  |  |  |  |  |  |  |  |  |  |  |  |  |  |  |  |  |  |  |  |  |  |  |  |  |  |  | ARI_16579875 | 0.345 |
| **111** | P0756 | PROKKA_00759 | 396 |  |  |  |  |  |  |  |  |  |  |  |  |  |  |  |  |  |  |  |  |  |  |  |  |  |  |  |  | PAI_CAE85251 | 0.333 |  |  |
| **112** | P0760 | PROKKA_00763 | 222 | VFG1297 | 0.360 |  |  |  |  |  |  |  |  |  |  |  |  |  |  |  |  |  |  |  |  |  |  |  |  |  |  |  |  | ARI_506697 | 0.522 |
| **113** | P0761 | PROKKA_00764 | 242 | VFG1312 | 0.616 |  |  |  |  |  |  |  |  |  |  |  |  |  |  |  |  |  |  |  |  |  |  |  |  |  |  |  |  |  |  |
| **114** | P0762 | PROKKA_00765 | 234 |  |  |  |  |  |  |  |  |  |  |  |  |  |  |  |  |  |  |  |  |  |  |  |  |  |  |  |  | PAI_AAO18070 | 0.393 |  |  |
| **115** | P0764 | PROKKA_00767 | 258 |  |  |  |  |  |  |  |  |  |  |  |  |  |  |  |  |  |  |  |  |  |  |  |  |  |  |  |  | PAI_331029102 | 0.190 | ARI_12698385 | 0.298 |
| **116** | P0767 | PROKKA_00770 | 440 |  |  |  |  |  |  |  |  |  |  |  |  |  |  |  |  |  |  |  |  |  |  |  |  |  |  |  |  |  |  | ARI_14021018 | 0.327 |
| **117** | P0769 | PROKKA_00772 | 459 |  |  |  |  |  |  |  |  |  |  |  |  |  |  |  |  |  |  |  |  |  |  |  |  |  |  |  |  | PAI_CAA21364 | 0.342 |  |  |
| **118** | P0778 | PROKKA_00781 | 431 | VFG1110 | 0.357 |  |  |  |  |  |  |  |  |  |  |  |  |  |  |  |  |  |  |  |  |  |  |  |  |  |  |  |  |  |  |
| **119** | P0781 | PROKKA_00784 | 273 |  |  |  |  |  |  |  |  |  |  |  |  |  |  |  |  |  |  |  |  |  |  |  |  |  |  |  |  | PAI_AAO17195 | 0.253 | ARI_12698385 | 0.238 |
| **120** | P0784 | PROKKA_00787 | 393 |  |  | AR_CAA78046 | 0.257 |  |  |  |  |  |  |  |  |  |  |  |  |  |  |  |  |  |  |  |  |  |  |  |  |  |  |  |  |
| **121** | P0787 | PROKKA_00790 | 259 |  |  |  |  |  |  |  |  |  |  |  |  |  |  |  |  |  |  |  |  |  |  |  |  |  |  |  |  | PAI_CAA21363 | 0.285 |  |  |
| **122** | P0792 | PROKKA_00795 | 460 |  |  |  |  |  |  |  |  |  |  |  |  |  |  |  |  |  |  |  |  |  |  |  |  |  |  |  |  | PAI_CAA21364 | 0.356 |  |  |
| **123** | P0797 | PROKKA_00800 | 215 |  |  | AR_NP_932197 | 0.488 |  |  |  |  |  |  |  |  |  |  |  |  |  |  |  |  |  |  |  |  |  |  |  |  |  |  |  |  |
| **124** | P0798 | PROKKA_00801 | 468 |  |  | AR_NP_388149 | 0.417 |  |  |  |  |  |  |  |  |  |  |  |  |  |  |  |  |  |  |  |  |  |  |  |  |  |  |  |  |
| **125** | P0814 | PROKKA_00817 | 358 |  |  |  |  |  |  |  |  |  |  |  |  |  |  |  |  |  |  |  |  |  |  |  |  |  |  |  |  |  |  | ARI_484356542 | 0.243 |
| **126** | P0817 | PROKKA_00820 | 222 |  |  |  |  |  |  |  |  |  |  |  |  | Prophage_56693115 | 0.302 |  |  |  |  |  |  |  |  |  |  |  |  |  |  |  |  |  |  |
| **127** | P0818 | PROKKA_00821 | 205 |  |  |  |  |  |  |  |  |  |  |  |  | Prophage_56693115 | 0.271 |  |  |  |  |  |  |  |  |  |  |  |  |  |  |  |  |  |  |
| **128** | P0833 | PROKKA_00836 | 455 |  |  |  |  |  |  |  |  |  |  |  |  | Prophage_38707949 | 0.284 |  |  |  |  |  |  |  |  |  |  |  |  |  |  | PAI_CAA21369 | 0.169 |  |  |
| **129** | P0836 | PROKKA_00839 | 333 | VFG2162 | 0.276 |  |  |  |  |  |  |  |  |  |  |  |  |  |  |  |  |  |  |  |  |  |  |  |  |  |  |  |  |  |  |
| **130** | P0865 | PROKKA_00868 | 232 |  |  |  |  |  |  |  |  |  |  |  |  |  |  |  |  |  |  |  |  |  |  |  |  |  |  | IS231K_PEP3 | 0.310 |  |  |  |  |
| **131** | P0878 | PROKKA_00882 | 487 |  |  |  |  |  |  |  |  |  |  |  |  |  |  |  |  |  |  |  |  |  |  |  |  |  |  |  |  | PAI_CAF28571 | 0.334 |  |  |
| **132** | P0895 | PROKKA_00899 | 242 |  |  |  |  |  |  |  |  |  |  |  |  | Prophage_9630246 | 0.289 |  |  |  |  |  |  |  |  |  |  |  |  |  |  |  |  |  |  |
| **133** | P0896 | PROKKA_00900 | 472 |  |  | AR_YP_001419928 | 0.371 |  |  |  |  |  |  |  |  |  |  |  |  |  |  |  |  |  |  |  |  |  |  |  |  |  |  |  |  |
| **134** | P0898 | PROKKA_00902 | 400 | VFG1661 | 0.263 |  |  |  |  |  |  |  |  |  |  |  |  |  |  |  |  |  |  |  |  |  |  |  |  |  |  | PAI_CAD66186 | 0.263 |  |  |
| **135** | P0919 | PROKKA_00923 | 213 |  |  |  |  |  |  |  |  |  |  |  |  |  |  |  |  |  |  |  |  |  |  |  |  |  |  |  |  | PAI_AAO17184 | 0.299 |  |  |
| **136** | P0920 | PROKKA_00924 | 189 | VFG1404 | 0.270 |  |  |  |  |  |  |  |  |  |  |  |  |  |  |  |  |  |  |  |  |  |  |  |  |  |  |  |  |  |  |
| **137** | P0929 | PROKKA_00933 | 104 |  |  | AR_NP_389193 | 0.538 |  |  |  |  |  |  |  |  |  |  |  |  |  |  |  |  |  |  |  |  |  |  |  |  |  |  |  |  |
| **138** | P0930 | PROKKA_00934 | 108 |  |  | AR_YP_001420884 | 0.546 |  |  |  |  |  |  |  |  |  |  |  |  |  |  |  |  |  |  |  |  |  |  |  |  |  |  |  |  |
| **139** | P0934 | PROKKA_00938 | 382 |  |  |  |  |  |  |  |  |  |  |  |  |  |  |  |  |  |  |  |  |  |  |  |  |  |  |  |  |  |  | ARI_28465863 | 0.675 |
| **140** | P0942 | PROKKA_00946 | 472 |  |  |  |  |  |  |  |  |  |  |  |  |  |  |  |  |  |  |  |  |  |  |  |  |  |  |  |  | PAI_CAA21342 | 0.263 |  |  |
| **141** | P0944 | PROKKA_00948 | 220 |  |  | AR_YP_002382193 | 0.376 |  |  |  |  |  |  |  |  |  |  |  |  |  |  |  |  |  |  |  |  |  |  |  |  |  |  |  |  |
| **142** | P0946 | PROKKA_00950 | 125 |  |  |  |  |  |  |  |  |  |  |  |  | Prophage_134287378 | 0.200 |  |  |  |  |  |  |  |  |  |  |  |  |  |  |  |  | ARI_5360848 | 0.432 |
| **143** | P0947 | PROKKA_00951 | 583 |  |  |  |  |  |  |  |  |  |  |  |  |  |  |  |  |  |  |  |  |  |  |  |  |  |  |  |  |  |  | ARI_28465862 | 0.324 |
| **144** | P0948 | PROKKA_00952 | 282 |  |  | AR_NP_395546 | 0.479 |  |  |  |  |  |  |  |  |  |  |  |  |  |  |  |  |  |  |  |  |  |  |  |  |  |  |  |  |
| **145** | P0949 | PROKKA_00953 | 141 |  |  |  |  |  |  |  |  |  |  |  |  |  |  |  |  |  |  |  |  |  |  |  |  |  |  |  |  |  |  | ARI_7592627 | 0.617 |
| **146** | P0952 | PROKKA_00956 | 311 |  |  |  |  |  |  |  |  |  |  |  |  |  |  |  |  |  |  |  |  |  |  |  |  |  |  |  |  |  |  | ARI_221148478 | 0.286 |
| **147** | P0958 | PROKKA_00962 | 250 |  |  |  |  |  |  |  |  |  |  |  |  | Prophage_9630246 | 0.336 |  |  |  |  |  |  |  |  |  |  |  |  |  |  |  |  |  |  |
| **148** | P0960 | PROKKA_00964 | 177 |  |  |  |  |  |  |  |  |  |  |  |  | Prophage_66396047 | 0.407 |  |  |  |  |  |  |  |  |  |  |  |  |  |  |  |  |  |  |
| **149** | P0961 | PROKKA_00965 | 186 |  |  |  |  |  |  |  |  |  |  |  |  |  |  |  |  |  |  |  |  |  |  |  |  |  |  |  |  | PAI_AAW49312 | 0.317 |  |  |
| **150** | P0965 | PROKKA_00969 | 153 |  |  |  |  |  |  |  |  |  |  |  |  |  |  |  |  |  |  |  |  |  |  |  |  |  |  |  |  |  |  | ARI_14021013 | 0.301 |
| **151** | P0968 | PROKKA_00972 | 220 |  |  |  |  |  |  |  |  |  |  |  |  | Prophage_134288743 | 0.314 |  |  |  |  |  |  |  |  |  |  |  |  |  |  |  |  |  |  |
| **152** | P0970 | PROKKA_00974 | 231 | VFG0169 | 0.268 |  |  |  |  |  |  |  |  |  |  |  |  |  |  |  |  |  |  |  |  |  |  |  |  |  |  | PAI_CAA21393 | 0.255 |  |  |
| **153** | P0972 | PROKKA_00976 | 1658 | VFG0161 | 0.281 |  |  |  |  |  |  |  |  |  |  |  |  |  |  |  |  |  |  |  |  |  |  |  |  |  |  |  |  |  |  |
| **154** | P0973 | PROKKA_00977 | 921 | VFG0161 | 0.329 |  |  |  |  |  |  |  |  |  |  |  |  |  |  |  |  |  |  |  |  |  |  |  |  |  |  |  |  |  |  |
| **155** | P0974 | PROKKA_00978 | 912 | VFG0161 | 0.290 |  |  |  |  |  |  |  |  |  |  |  |  |  |  |  |  |  |  |  |  |  |  |  |  |  |  |  |  |  |  |
| **156** | P0975 | PROKKA_00979 | 173 |  |  |  |  |  |  |  |  |  |  |  |  | Prophage_118725075 | 0.630 |  |  |  |  |  |  |  |  |  |  |  |  |  |  |  |  |  |  |
| **157** | P0977 | PROKKA_00981 | 261 |  |  |  |  |  |  |  |  |  |  |  |  | Prophage_56693136 | 0.310 |  |  |  |  |  |  |  |  |  |  |  |  |  |  |  |  |  |  |
| **158** | P0990 | PROKKA_00994 | 289 |  |  |  |  |  |  |  |  |  |  |  |  |  |  |  |  |  |  |  |  |  |  |  |  |  |  |  |  | PAI_AAO17195 | 0.304 | ARI_12698385 | 0.291 |
| **159** | P0998 | PROKKA_01002 | 178 | VFG1601 | 0.264 |  |  |  |  |  |  |  |  |  |  |  |  |  |  |  |  |  |  |  |  |  |  |  |  |  |  |  |  |  |  |
| **160** | P0999 | PROKKA_01003 | 102 |  |  | AR_NP_783299 | 0.333 |  |  |  |  |  |  |  |  |  |  |  |  |  |  |  |  |  |  |  |  |  |  |  |  |  |  |  |  |
| **161** | P1000 | PROKKA_01004 | 113 | VFG1586 | 0.248 | AR_O87866 | 0.345 |  |  |  |  |  |  |  |  |  |  |  |  |  |  |  |  |  |  |  |  |  |  |  |  |  |  |  |  |
| **162** | P1005 | PROKKA_01009 | 322 |  |  |  |  |  |  |  |  |  |  |  |  | Prophage_48696469 | 0.552 |  |  |  |  |  |  |  |  |  |  |  |  |  |  |  |  |  |  |
| **163** | P1006 | PROKKA_01010 | 701 |  |  |  |  |  |  |  |  |  |  |  |  | Prophage_48696468 | 0.479 |  |  |  |  |  |  |  |  |  |  |  |  |  |  |  |  |  |  |
| **164** | P1007 | PROKKA_01011 | 132 |  |  |  |  |  |  |  |  |  |  |  |  | Prophage_9630285 | 0.341 |  |  |  |  |  |  |  |  |  |  |  |  |  |  |  |  |  |  |
| **165** | P1010 | PROKKA_01014 | 504 | VFG1857 | 0.222 |  |  |  |  |  |  |  |  |  |  |  |  |  |  |  |  |  |  |  |  |  |  |  |  |  |  | PAI_AAO17199 | 0.246 |  |  |
| **166** | P1013 | PROKKA_01017 | 178 |  |  |  |  |  |  |  |  |  |  |  |  | Prophage_9630265 | 0.343 |  |  |  |  |  |  |  |  |  |  |  |  |  |  |  |  |  |  |
| **167** | P1014 | PROKKA_01018 | 351 | VFG1965 | 0.279 |  |  |  |  |  |  |  |  |  |  |  |  |  |  |  |  |  |  |  |  |  |  |  |  |  |  |  |  |  |  |
| **168** | P1016 | PROKKA_01020 | 318 | VFG1206 | 0.270 |  |  |  |  |  |  |  |  |  |  |  |  |  |  |  |  |  |  |  |  |  |  |  |  |  |  |  |  |  |  |
| **169** | P1018 | PROKKA_01022 | 627 |  |  | AR_AAC32027 | 0.254 |  |  |  |  |  |  |  |  |  |  |  |  |  |  |  |  |  |  |  |  |  |  |  |  |  |  |  |  |
| **170** | P1027 | PROKKA_01031 | 224 |  |  |  |  |  |  |  |  |  |  |  |  | Prophage_146329947 | 0.385 |  |  |  |  |  |  |  |  |  |  |  |  |  |  |  |  |  |  |
| **171** | P1029 | PROKKA_01033 | 238 |  |  |  |  |  |  |  |  |  |  |  |  | Prophage_109521763 | 0.249 |  |  |  |  |  |  |  |  |  |  |  |  |  |  |  |  |  |  |
| **172** | P1032 | PROKKA_01036 | 348 |  |  |  |  |  |  |  |  |  |  |  |  | Prophage_9635491 | 0.379 |  |  |  |  |  |  |  |  |  |  |  |  |  |  |  |  |  |  |
| **173** | P1035 | PROKKA_01039 | 387 | VFG1116 | 0.253 |  |  |  |  |  |  |  |  |  |  |  |  |  |  |  |  |  |  |  |  |  |  |  |  |  |  |  |  |  |  |
| **174** | P1038 | PROKKA_01042 | 252 |  |  |  |  |  |  |  |  |  |  |  |  |  |  |  |  |  |  |  |  |  |  |  |  |  |  |  |  | PAI_CAA21363 | 0.333 |  |  |
| **175** | P1039 | PROKKA_01043 | 290 | VFG0964 | 0.543 |  |  |  |  |  |  |  |  |  |  |  |  |  |  |  |  |  |  |  |  |  |  |  |  |  |  |  |  | ARI_697403908 | 0.428 |
| **176** | P1041 | PROKKA_01045 | 388 |  |  | AR_YP_302113 | 0.833 |  |  |  |  |  |  |  |  |  |  |  |  |  |  |  |  |  |  |  |  |  |  |  |  |  |  |  |  |
| **177** | P1053 | PROKKA_01057 | 291 |  |  | AR_YP_302125 | 0.787 |  |  |  |  |  |  |  |  |  |  |  |  |  |  |  |  |  |  |  |  |  |  |  |  |  |  |  |  |
| **178** | P1056 | PROKKA_01060 | 188 |  |  |  |  |  |  |  |  |  |  |  |  |  |  |  |  |  |  |  |  |  |  |  |  |  |  |  |  | PAI_AAW49312 | 0.356 |  |  |
| **179** | P1062 | PROKKA_01066 | 386 |  |  | AR_AAB36568 | 0.241 |  |  |  |  |  |  |  |  |  |  |  |  |  |  |  |  |  |  |  |  |  |  | IS5564_PEP | 0.205 |  |  |  |  |
| **180** | P1075 | PROKKA_01079 | 224 | VFG0596 | 0.304 |  |  |  |  |  |  |  |  |  |  |  |  |  |  |  |  |  |  |  |  |  |  |  |  |  |  |  |  |  |  |
| **181** | P1084 | PROKKA_01088 | 338 | VFG0922 | 0.278 |  |  |  |  |  |  |  |  |  |  |  |  |  |  |  |  |  |  |  |  |  |  |  |  |  |  |  |  | ARI_15808723 | 0.305 |
| **182** | P1085 | PROKKA_01089 | 333 | VFG0926 | 0.333 |  |  |  |  |  |  |  |  |  |  |  |  |  |  |  |  |  |  |  |  |  |  |  |  |  |  |  |  |  |  |
| **183** | P1086 | PROKKA_01090 | 267 | VFG0925 | 0.457 |  |  |  |  |  |  |  |  |  |  |  |  |  |  |  |  |  |  |  |  |  |  |  |  |  |  |  |  | ARI_15808722 | 0.431 |
| **184** | P1089 | PROKKA_01093 | 573 | VFG0907 | 0.274 |  |  |  |  |  |  |  |  |  |  |  |  |  |  |  |  |  |  |  |  |  |  |  |  |  |  | PAI_CAD33760 | 0.274 |  |  |
| **185** | P1091 | PROKKA_01095 | 132 | VFG2027 | 0.227 |  |  |  |  |  |  |  |  |  |  | Prophage_61806180 | 0.333 |  |  |  |  |  |  |  |  |  |  |  |  |  |  |  |  |  |  |
| **186** | P1095 | PROKKA_01099 | 264 | VFG0258 | 0.307 |  |  |  |  |  |  |  |  |  |  |  |  |  |  |  |  |  |  |  |  |  |  |  |  |  |  |  |  |  |  |
| **187** | P1099 | PROKKA_01103 | 246 | VFG0526 | 0.325 |  |  |  |  |  |  |  |  |  |  |  |  |  |  |  |  |  |  |  |  |  |  |  |  |  |  | PAI_CAD33755 | 0.264 |  |  |
| **188** | P1100 | PROKKA_01104 | 278 | VFG0528 | 0.320 |  |  |  |  |  |  |  |  |  |  |  |  |  |  |  |  |  |  |  |  |  |  |  |  |  |  |  |  |  |  |
| **189** | P1101 | PROKKA_01105 | 310 | VFG2157 | 0.490 |  |  |  |  |  |  |  |  |  |  |  |  |  |  |  |  |  |  |  |  |  |  |  |  |  |  |  |  |  |  |
| **190** | P1110 | PROKKA_01114 | 183 |  |  |  |  |  |  |  |  |  |  |  |  | Prophage_31415840 | 0.317 |  |  |  |  |  |  |  |  |  |  |  |  |  |  |  |  |  |  |
| **191** | P1116 | PROKKA_01120 | 191 | VFG1411 | 0.393 |  |  |  |  |  |  |  |  |  |  |  |  |  |  |  |  |  |  |  |  |  |  |  |  |  |  |  |  |  |  |
| **192** | P1126 | PROKKA_01130 | 154 |  |  |  |  |  |  | T4SE_29654129 | 0.325 |  |  |  |  |  |  |  |  |  |  |  |  |  |  |  |  |  |  |  |  |  |  |  |  |
| **193** | P1130 | PROKKA_01134 | 639 |  |  | AR_CAA45050 | 0.246 |  |  |  |  |  |  |  |  |  |  |  |  |  |  |  |  |  |  |  |  |  |  |  |  |  |  |  |  |
| **194** | P1134 | PROKKA_01138 | 319 | VFG2197 | 0.244 |  |  |  |  |  |  |  |  |  |  |  |  |  |  |  |  |  |  |  |  |  |  |  |  |  |  |  |  |  |  |
| **195** | P1146 | PROKKA_01150 | 94 |  |  |  |  |  |  |  |  |  |  |  |  | Prophage_156564025 | 0.351 |  |  |  |  |  |  |  |  |  |  |  |  |  |  |  |  |  |  |
| **196** | P1147 | PROKKA_01151 | 540 | VFG1855 | 0.559 |  |  |  |  |  |  |  |  |  |  |  |  |  |  |  |  |  |  |  |  |  |  |  |  |  |  |  |  | ARI_481190498 | 0.106 |
| **197** | P1148 | PROKKA_01152 | 88 |  |  |  |  |  |  |  |  |  |  |  |  |  |  |  |  |  |  |  |  |  |  |  |  |  |  |  |  |  |  | ARI_672940411 | 0.307 |
| **198** | P1157 | PROKKA_01161 | 290 |  |  | AR_ZP_04081918 | 0.293 |  |  |  |  |  |  |  |  |  |  |  |  |  |  |  |  |  |  |  |  |  |  |  |  |  |  |  |  |
| **199** | P1165 | PROKKA_01169 | 132 |  |  |  |  |  |  |  |  |  |  |  |  |  |  |  |  |  |  |  |  |  |  |  |  |  |  |  |  |  |  | ARI_672940410 | 0.341 |
| **200** | P1167 | PROKKA_01171 | 104 |  |  |  |  |  |  |  |  |  |  |  |  |  |  |  |  |  |  |  |  |  |  |  |  |  |  |  |  |  |  | ARI_28465871 | 0.260 |
| **201** | P1169 | PROKKA_01173 | 459 |  |  |  |  |  |  |  |  |  |  |  |  |  |  |  |  |  |  |  |  |  |  |  |  |  |  |  |  | PAI_AAO17183 | 0.242 | ARI_12698392 | 0.200 |
| **202** | P1173 | PROKKA_01177 | 520 |  |  |  |  |  |  |  |  |  |  |  |  |  |  |  |  |  |  |  |  |  |  |  |  |  |  |  |  | PAI_AAO17231 | 0.275 |  |  |
| **203** | P1175 | PROKKA_01179 | 354 |  |  |  |  |  |  |  |  |  |  |  |  |  |  |  |  |  |  |  |  |  |  |  |  |  |  |  |  | PAI_AAW49313 | 0.452 |  |  |
| **204** | P1184 | PROKKA_01188 | 732 |  |  |  |  |  |  |  |  |  |  |  |  | Prophage_156564011 | 0.363 |  |  |  |  |  |  |  |  |  |  |  |  |  |  |  |  |  |  |
| **205** | P1185 | PROKKA_01189 | 668 |  |  |  |  |  |  |  |  |  |  |  |  | Prophage_80159718 | 0.327 |  |  |  |  |  |  |  |  |  |  |  |  |  |  |  |  |  |  |
| **206** | P1194 | PROKKA_01198 | 184 |  |  |  |  |  |  |  |  |  |  |  |  | Prophage_66395669 | 0.321 |  |  |  |  |  |  |  |  |  |  |  |  |  |  |  |  |  |  |
| **207** | P1203 | PROKKA_01207 | 209 | VFG1889 | 0.335 |  |  |  |  |  |  |  |  |  |  |  |  |  |  |  |  |  |  |  |  |  |  |  |  |  |  |  |  |  |  |
| **208** | P1206 | PROKKA_01210 | 154 |  |  |  |  |  |  |  |  |  |  |  |  |  |  |  |  |  |  |  |  |  |  |  |  |  |  |  |  |  |  | ARI_484359520 | 0.273 |
| **209** | P1220 | PROKKA_01226 | 256 | VFG1413 | 0.320 |  |  |  |  |  |  |  |  |  |  |  |  |  |  |  |  |  |  |  |  |  |  |  |  |  |  |  |  |  |  |
| **210** | P1224 | PROKKA_01230 | 118 |  |  |  |  |  |  |  |  |  |  |  |  | Prophage_80159713 | 0.317 |  |  |  |  |  |  |  |  |  |  |  |  |  |  |  |  |  |  |
| **211** | P1226 | PROKKA_01232 | 382 |  |  | AR_AAQ16274 | 0.309 |  |  |  |  |  |  |  |  |  |  |  |  |  |  |  |  |  |  |  |  |  |  |  |  |  |  |  |  |
| **212** | P1233 | PROKKA_01239 | 356 |  |  | AR_AAQ16273 | 0.325 |  |  |  |  |  |  |  |  |  |  |  |  |  |  |  |  |  |  |  |  |  |  |  |  |  |  |  |  |
| **213** | P1237 | PROKKA_01243 | 97 |  |  |  |  |  |  |  |  |  |  |  |  |  |  |  |  |  |  |  |  |  |  |  |  |  |  |  |  | PAI_950041 | 0.237 | ARI_13785453 | 0.247 |
| **214** | P1245 | PROKKA_01251 | 233 |  |  |  |  |  |  |  |  |  |  |  |  | Prophage_66395003 | 0.266 |  |  |  |  |  |  |  |  |  |  |  |  |  |  |  |  |  |  |
| **215** | P1246 | PROKKA_01252 | 122 |  |  |  |  |  |  |  |  |  |  |  |  | Prophage_66394702 | 0.361 |  |  |  |  |  |  |  |  |  |  |  |  |  |  |  |  |  |  |
| **216** | P1252 | PROKKA_01258 | 470 |  |  |  |  | T3SE_B0HZP9 | 0.274 |  |  |  |  |  |  |  |  |  |  | T3SS_YscN | 0.274 |  |  |  |  |  |  |  |  |  |  |  |  |  |  |
| **217** | P1260 | PROKKA_01266 | 376 | VFG1312 | 0.633 |  |  |  |  |  |  |  |  |  |  |  |  |  |  |  |  |  |  |  |  |  |  |  |  |  |  |  |  |  |  |
| **218** | P1262 | PROKKA_01268 | 412 |  |  |  |  |  |  |  |  |  |  |  |  | Prophage_66391812 | 0.495 |  |  |  |  |  |  |  |  |  |  |  |  |  |  |  |  |  |  |
| **219** | P1264 | PROKKA_01270 | 141 |  |  |  |  |  |  |  |  |  |  |  |  |  |  |  |  |  |  |  |  |  |  |  |  |  |  |  |  | PAI_AAO18065 | 0.270 | ARI_484359520 | 0.277 |
| **220** | P1268 | PROKKA_01274 | 199 |  |  |  |  |  |  |  |  |  |  |  |  | Prophage_156564177 | 0.347 |  |  |  |  |  |  |  |  |  |  |  |  |  |  |  |  |  |  |
| **221** | P1271 | PROKKA_01277 | 477 |  |  |  |  |  |  |  |  |  |  |  |  |  |  |  |  |  |  |  |  |  |  |  |  |  |  |  |  | PAI_AAO17179 | 0.371 |  |  |
| **222** | P1274 | PROKKA_01280 | 286 |  |  |  |  |  |  |  |  |  |  |  |  |  |  |  |  |  |  |  |  |  |  |  |  |  |  |  |  | PAI_AAO17213 | 0.256 |  |  |
| **223** | P1282 | PROKKA_01288 | 394 |  |  |  |  |  |  |  |  |  |  |  |  |  |  |  |  |  |  |  |  |  |  |  |  |  |  |  |  |  |  | ARI_16579875 | 0.254 |
| **224** | P1288 | PROKKA_01294 | 148 | VFG0307 | 0.331 |  |  |  |  |  |  |  |  |  |  |  |  |  |  |  |  |  |  |  |  |  |  |  |  |  |  |  |  |  |  |
| **225** | P1295 | PROKKA_01301 | 101 |  |  |  |  |  |  |  |  |  |  |  |  |  |  |  |  |  |  |  |  |  |  |  |  |  |  |  |  |  |  | ARI_28465871 | 0.327 |
| **226** | P1307 | PROKKA_01313 | 450 |  |  |  |  |  |  |  |  |  |  |  |  |  |  |  |  |  |  |  |  |  |  |  |  |  |  |  |  |  |  | ARI_697403912 | 0.263 |
| **227** | P1311 | PROKKA_01324 | 322 |  |  |  |  |  |  |  |  |  |  |  |  |  |  |  |  |  |  |  |  |  |  |  |  |  |  |  |  | PAI_AAL08452 | 0.258 | ARI_15808723 | 0.258 |
| **228** | P1312 | PROKKA_01325 | 319 | VFG0926 | 0.257 |  |  |  |  |  |  |  |  |  |  |  |  |  |  |  |  |  |  |  |  |  |  |  |  |  |  |  |  |  |  |
| **229** | P1313 | PROKKA_01326 | 264 |  |  |  |  |  |  |  |  |  |  |  |  |  |  |  |  |  |  |  |  |  |  |  |  |  |  |  |  | PAI_AAL08451 | 0.341 | ARI_15808722 | 0.341 |
| **230** | P1346 | PROKKA_01359 | 159 |  |  | AR_YP_001038094 | 0.170 |  |  |  |  |  |  |  |  |  |  |  |  |  |  |  |  |  |  |  |  |  |  |  |  |  |  | ARI_506703 | 0.245 |
| **231** | P1348 | PROKKA_01361 | 314 |  |  |  |  |  |  |  |  |  |  |  |  |  |  |  |  |  |  |  |  |  |  |  |  |  |  |  |  |  |  | ARI_672940407 | 0.404 |
| **232** | P1354 | PROKKA_01368 | 194 | VFG0077 | 0.773 |  |  |  |  |  |  |  |  |  |  | Prophage_109302855 | 0.273 |  |  |  |  |  |  |  |  |  |  |  |  |  |  |  |  |  |  |
| **233** | P1360 | PROKKA_01374 | 335 |  |  |  |  |  |  |  |  |  |  |  |  |  |  |  |  |  |  |  |  |  |  |  |  |  |  |  |  | PAI_AAO17215 | 0.391 |  |  |
| **234** | P1361 | PROKKA_01375 | 396 |  |  |  |  |  |  |  |  |  |  |  |  |  |  |  |  |  |  |  |  |  |  |  |  |  |  |  |  | PAI_AAO17214 | 0.444 |  |  |
| **235** | P1368 | PROKKA_01382 | 795 |  |  |  |  |  |  |  |  |  |  |  |  | Prophage_115304286 | 0.243 |  |  |  |  |  |  |  |  |  |  |  |  |  |  |  |  |  |  |
| **236** | P1371 | PROKKA_01386 | 368 |  |  |  |  |  |  |  |  |  |  |  |  | Prophage_20065985 | 0.302 |  |  |  |  |  |  |  |  |  |  |  |  |  |  |  |  |  |  |
| **237** | P1378 | PROKKA_01393 | 267 |  |  |  |  |  |  |  |  |  |  |  |  | Prophage_66395168 | 0.738 |  |  |  |  |  |  |  |  |  |  |  |  |  |  |  |  |  |  |
| **238** | P1379 | PROKKA_01394 | 91 |  |  |  |  |  |  |  |  |  |  |  |  | Prophage_66395181 | 0.615 |  |  |  |  |  |  |  |  |  |  |  |  |  |  |  |  |  |  |
| **239** | P1381 | PROKKA_01396 | 122 |  |  |  |  |  |  |  |  |  |  |  |  |  |  |  |  |  |  |  |  |  |  |  |  |  |  |  |  | PAI_AAL04135 | 0.271 |  |  |
| **240** | P1383 | PROKKA_01398 | 121 |  |  |  |  |  |  |  |  |  |  |  |  | Prophage_118725091 | 0.579 |  |  |  |  |  |  |  |  |  |  |  |  |  |  |  |  |  |  |
| **241** | P1386 | PROKKA_01401 | 156 |  |  |  |  |  |  |  |  |  |  |  |  |  |  |  |  |  |  |  |  |  |  |  |  |  |  |  |  | PAI_AAL04129 | 0.423 |  |  |
| **242** | P1387 | PROKKA_01402 | 111 |  |  |  |  |  |  |  |  |  |  |  |  | Prophage_66395178 | 0.342 |  |  |  |  |  |  |  |  |  |  |  |  |  |  | PAI_AAC28955 | 0.342 |  |  |
| **243** | P1394 | PROKKA_01409 | 151 |  |  | AR_YP_302016 | 0.815 |  |  |  |  |  |  |  |  |  |  |  |  |  |  |  |  |  |  |  |  |  |  |  |  |  |  |  |  |
| **244** | P1402 | PROKKA_01417 | 183 |  |  |  |  |  |  |  |  |  |  |  |  | Prophage_41179219 | 0.306 |  |  |  |  |  |  |  |  |  |  |  |  |  |  |  |  |  |  |
| **245** | P1411 | PROKKA_01426 | 429 |  |  |  |  |  |  |  |  |  |  |  |  |  |  |  |  |  |  |  |  |  |  |  |  |  |  |  |  | PAI_CAA21341 | 0.254 | ARI_12698391 | 0.245 |
| **246** | P1425 | PROKKA_01440 | 497 |  |  |  |  |  |  |  |  |  |  |  |  |  |  |  |  |  |  |  |  |  |  |  |  |  |  |  |  | PAI_CAA21364 | 0.266 |  |  |
| **247** | P1430 | PROKKA_01445 | 341 |  |  |  |  |  |  |  |  |  |  |  |  |  |  |  |  |  |  |  |  |  |  |  |  |  |  |  |  | PAI_AAO17215 | 0.393 |  |  |
| **248** | P1435 | PROKKA_01450 | 236 |  |  | AR_AAY52009 | 0.424 |  |  |  |  |  |  |  |  |  |  |  |  |  |  |  |  |  |  |  |  |  |  |  |  |  |  |  |  |
| **249** | P1438 | PROKKA_01453 | 453 |  |  |  |  |  |  |  |  |  |  |  |  |  |  |  |  |  |  |  |  |  |  |  |  |  |  |  |  | PAI_CAA21364 | 0.313 |  |  |
| **250** | P1439 | PROKKA_01454 | 586 | VFG0479 | 0.399 |  |  |  |  |  |  |  |  |  |  |  |  |  |  |  |  |  |  |  |  |  |  |  |  |  |  |  |  |  |  |
| **251** | P1444 | PROKKA_01459 | 1063 |  |  |  |  |  |  |  |  |  |  |  |  | Prophage_29566684 | 0.252 |  |  |  |  |  |  |  |  |  |  |  |  |  |  |  |  |  |  |
| **252** | P1456 | PROKKA_01471 | 164 | VFG0087 | 0.329 |  |  |  |  |  |  |  |  |  |  |  |  |  |  |  |  |  |  |  |  |  |  |  |  |  |  | PAI_AAK20781 | 0.329 |  |  |
| **253** | P1459 | PROKKA_01474 | 257 |  |  |  |  |  |  |  |  |  |  |  |  |  |  |  |  |  |  |  |  |  |  |  |  |  |  |  |  | PAI_ABW69077 | 0.296 |  |  |
| **254** | P1461 | PROKKA_01476 | 378 |  |  |  |  |  |  |  |  |  |  |  |  |  |  |  |  |  |  |  |  |  |  |  |  |  |  |  |  | PAI_ABP49156 | 0.330 |  |  |
| **255** | P1465 | PROKKA_01480 | 165 |  |  |  |  |  |  |  |  |  |  |  |  | Prophage_9630137 | 0.248 |  |  |  |  |  |  |  |  |  |  |  |  |  |  |  |  | ARI_484356517 | 0.261 |
| **256** | P1466 | PROKKA_01481 | 245 |  |  |  |  |  |  |  |  |  |  |  |  |  |  |  |  |  |  |  |  |  |  |  |  |  |  |  |  |  |  | ARI_28465859 | 0.316 |
| **257** | P1471 | PROKKA_01486 | 121 |  |  |  |  |  |  |  |  |  |  |  |  | Prophage_66395003 | 0.248 |  |  |  |  |  |  |  |  |  |  |  |  |  |  |  |  |  |  |
| **258** | P1473 | PROKKA_01488 | 476 |  |  | AR_NP_388149 | 0.382 |  |  |  |  |  |  |  |  |  |  |  |  |  |  |  |  |  |  |  |  |  |  |  |  |  |  |  |  |
| **259** | P1477 | PROKKA_01492 | 323 | VFG1330 | 0.272 |  |  |  |  |  |  |  |  |  |  |  |  |  |  |  |  |  |  |  |  |  |  |  |  |  |  |  |  |  |  |
| **260** | P1495 | PROKKA_01510 | 244 |  |  |  |  |  |  |  |  |  |  |  |  |  |  |  |  |  |  |  |  |  |  |  |  |  |  |  |  | PAI_AAO17195 | 0.329 | ARI_12698385 | 0.324 |
| **261** | P1518 | PROKKA_01533 | 224 | VFG0596 | 0.290 | AR_CAB61229 | 0.351 |  |  |  |  |  |  |  |  |  |  |  |  |  |  |  |  |  |  |  |  |  |  |  |  |  |  |  |  |
| **262** | P1520 | PROKKA_01535 | 222 |  |  | AR_YP_001176128 | 0.410 |  |  |  |  |  |  |  |  |  |  |  |  |  |  |  |  |  |  |  |  |  |  |  |  |  |  |  |  |
| **263** | P1525 | PROKKA_01540 | 396 |  |  | AR_ZP_03837521 | 0.303 |  |  |  |  |  |  |  |  |  |  |  |  |  |  |  |  |  |  |  |  |  |  |  |  |  |  |  |  |
| **264** | P1528 | PROKKA_01543 | 622 |  |  | AR_NP_388149 | 0.272 |  |  |  |  |  |  |  |  |  |  |  |  |  |  |  |  |  |  |  |  |  |  |  |  |  |  |  |  |
| **265** | P1538 | PROKKA_01553 | 299 |  |  | AR_ABB80128 | 0.244 |  |  |  |  |  |  |  |  |  |  |  |  |  |  |  |  |  |  |  |  |  |  |  |  |  |  |  |  |
| **266** | P1545 | PROKKA_01560 | 291 |  |  |  |  |  |  |  |  |  |  |  |  |  |  |  |  |  |  |  |  |  |  |  |  |  |  |  |  | PAI_CAA21367 | 0.244 | ARI_24461567 | 0.151 |
| **267** | P1547 | PROKKA_01562 | 412 |  |  |  |  |  |  |  |  |  |  |  |  |  |  |  |  |  |  |  |  |  |  |  |  |  |  |  |  | PAI_CAA21331 | 0.318 |  |  |
| **268** | P1548 | PROKKA_01563 | 373 |  |  |  |  |  |  |  |  |  |  |  |  |  |  |  |  |  |  |  |  |  |  |  |  |  |  |  |  |  |  | ARI_16579875 | 0.322 |
| **269** | P1552 | PROKKA_01567 | 253 | VFG1108 | 0.241 |  |  |  |  |  |  |  |  |  |  |  |  |  |  |  |  |  |  |  |  |  |  |  |  |  |  |  |  |  |  |
| **270** | P1560 | PROKKA_01575 | 458 |  |  |  |  |  |  |  |  |  |  |  |  |  |  |  |  |  |  |  |  |  |  |  |  |  |  |  |  | PAI_CAA21364 | 0.336 |  |  |
| **271** | P1567 | PROKKA_01582 | 231 |  |  |  |  |  |  |  |  |  |  |  |  |  |  |  |  |  |  |  |  |  |  |  |  |  |  |  |  |  |  | ARI_221148478 | 0.251 |
| **272** | P1573 | PROKKA_01588 | 258 |  |  |  |  |  |  |  |  |  |  |  |  | Prophage_66395374 | 0.372 |  |  |  |  |  |  |  |  |  |  |  |  |  |  |  |  |  |  |
| **273** | P1574 | PROKKA_01589 | 374 | VFG0173 | 0.246 | AR_Q01911 | 0.249 |  |  |  |  |  |  |  |  |  |  |  |  |  |  |  |  |  |  |  |  |  |  |  |  |  |  |  |  |
| **274** | P1575 | PROKKA_01590 | 318 |  |  | AR_AAD41881 | 0.267 |  |  |  |  |  |  |  |  |  |  |  |  |  |  |  |  |  |  |  |  |  |  |  |  |  |  |  |  |
| **275** | P1576 | PROKKA_01591 | 104 |  |  | AR_YP_001420885 | 0.505 |  |  |  |  |  |  |  |  |  |  |  |  |  |  |  |  |  |  |  |  |  |  |  |  |  |  |  |  |
| **276** | P1577 | PROKKA_01592 | 107 |  |  | AR_YP_001420884 | 0.551 |  |  |  |  |  |  |  |  |  |  |  |  |  |  |  |  |  |  |  |  |  |  |  |  |  |  |  |  |
| **277** | P1578 | PROKKA_01593 | 155 | VFG1355 | 0.181 |  |  |  |  |  |  |  |  |  |  |  |  |  |  |  |  |  |  |  |  |  |  |  |  |  |  |  |  | ARI_238773869 | 0.277 |
| **278** | P1590 | PROKKA_01605 | 249 | VFG0344 | 0.325 |  |  |  |  |  |  |  |  |  |  |  |  |  |  |  |  |  |  |  |  |  |  |  |  |  |  | PAI_CAA21355 | 0.321 |  |  |
| **279** | P1596 | PROKKA_01611 | 333 | VFG2197 | 0.273 |  |  |  |  |  |  |  |  |  |  |  |  |  |  |  |  |  |  |  |  |  |  |  |  |  |  |  |  |  |  |
| **280** | P1598 | PROKKA_01613 | 332 | VFG2361 | 0.394 |  |  |  |  |  |  |  |  |  |  |  |  |  |  |  |  |  |  |  |  |  |  |  |  |  |  |  |  | ARI_484359538 | 0.384 |
| **281** | P1602 | PROKKA_01617 | 207 | VFG1206 | 0.348 |  |  |  |  |  |  |  |  |  |  |  |  |  |  |  |  |  |  |  |  |  |  |  |  |  |  |  |  |  |  |
| **282** | P1613 | PROKKA_01628 | 422 |  |  |  |  |  |  |  |  |  |  |  |  | Prophage_9630142 | 0.441 |  |  |  |  |  |  |  |  |  |  |  |  |  |  | PAI_CAI36137 | 0.209 |  |  |
| **283** | P1624 | PROKKA_01639 | 800 |  |  |  |  |  |  |  |  |  |  |  |  |  |  |  |  |  |  |  |  |  |  |  |  |  |  |  |  | PAI_151500324 | 0.345 |  |  |
| **284** | P1625 | PROKKA_01640 | 666 |  |  |  |  |  |  |  |  |  |  |  |  | Prophage_116326225 | 0.281 |  |  |  |  |  |  |  |  |  |  |  |  |  |  |  |  |  |  |
| **285** | P1632 | PROKKA_01647 | 52 |  |  |  |  |  |  |  |  |  |  |  |  | Prophage_119443708 | 0.788 |  |  |  |  |  |  |  |  |  |  |  |  |  |  |  |  |  |  |
| **286** | P1638 | PROKKA_01653 | 662 |  |  |  |  |  |  |  |  |  |  |  |  |  |  |  |  |  |  |  |  |  |  |  |  |  |  |  |  | PAI_AAO17218 | 0.461 |  |  |
| **287** | P1644 | PROKKA_01659 | 325 |  |  |  |  |  |  |  |  |  |  |  |  | Prophage_66395520 | 0.886 |  |  |  |  |  |  |  |  |  |  |  |  |  |  |  |  |  |  |
| **288** | P1647 | PROKKA_01662 | 480 |  |  |  |  |  |  |  |  |  |  |  |  |  |  |  |  |  |  |  |  |  |  |  |  |  |  |  |  | PAI_CAA21364 | 0.279 |  |  |
| **289** | P1658 | PROKKA_01673 | 176 |  |  |  |  |  |  |  |  |  |  |  |  | Prophage_9630131 | 0.364 |  |  |  |  |  |  |  |  |  |  |  |  |  |  | PAI_569550109 | 0.205 |  |  |
| **290** | P1668 | PROKKA_01683 | 729 |  |  |  |  |  |  |  |  |  |  |  |  | Prophage_149882852 | 0.388 |  |  |  |  |  |  |  |  |  |  |  |  |  |  |  |  |  |  |
| **291** | P1670 | PROKKA_01685 | 344 |  |  |  |  |  |  |  |  |  |  |  |  | Prophage_61806048 | 0.422 |  |  |  |  |  |  |  |  |  |  |  |  |  |  |  |  |  |  |
| **292** | P1671 | PROKKA_01686 | 188 |  |  |  |  |  |  |  |  |  |  |  |  | Prophage_61805923 | 0.319 |  |  |  |  |  |  |  |  |  |  |  |  |  |  |  |  |  |  |
| **293** | P1672 | PROKKA_01687 | 492 |  |  |  |  |  |  |  |  |  |  |  |  | Prophage_61806062 | 0.303 |  |  |  |  |  |  |  |  |  |  |  |  |  |  |  |  |  |  |
| **294** | P1696 | PROKKA_01711 | 468 |  |  |  |  |  |  |  |  |  |  |  |  |  |  |  |  |  |  |  |  |  |  |  |  |  |  |  |  |  |  | ARI_14021018 | 0.333 |
| **295** | P1698 | PROKKA_01713 | 364 | VFG0344 | 0.313 |  |  |  |  |  |  |  |  |  |  |  |  |  |  |  |  |  |  |  |  |  |  |  |  |  |  | PAI_CAA21405 | 0.291 |  |  |
| **296** | P1717 | PROKKA_01732 | 295 |  |  |  |  |  |  |  |  |  |  |  |  |  |  |  |  |  |  |  |  |  |  |  |  |  |  |  |  | PAI_CAA21372 | 0.278 | ARI_481190496 | 0.176 |
| **297** | P1733 | PROKKA_01748 | 645 |  |  |  |  |  |  |  |  |  |  |  |  | Prophage_80159785 | 0.265 |  |  |  |  |  |  |  |  |  |  |  |  |  |  |  |  |  |  |
| **298** | P1734 | PROKKA_01749 | 891 |  |  |  |  |  |  |  |  |  |  |  |  |  |  |  |  |  |  |  |  |  |  |  |  |  |  |  |  | PAI_149774749 | 0.455 |  |  |
| **299** | P1746 | PROKKA_01761 | 463 |  |  |  |  |  |  |  |  |  |  |  |  | Prophage_28876155 | 0.488 |  |  |  |  |  |  |  |  |  |  |  |  |  |  |  |  |  |  |
| **300** | P1749 | PROKKA_01766 | 468 |  |  | AR_NP_388149 | 0.449 |  |  |  |  |  |  |  |  |  |  |  |  |  |  |  |  |  |  |  |  |  |  |  |  |  |  |  |  |
| **301** | P1750 | PROKKA_01767 | 233 |  |  | AR_CAB61229 | 0.446 |  |  |  |  |  |  |  |  |  |  |  |  |  |  |  |  |  |  |  |  |  |  |  |  |  |  |  |  |
| **302** | P1755 | PROKKA_01772 | 159 |  |  |  |  |  |  |  |  |  |  |  |  |  |  |  |  |  |  |  |  |  |  |  |  |  |  |  |  |  |  | ARI_28465880 | 0.855 |
| **303** | P1758 | PROKKA_01775 | 400 |  |  | AR_YP_402564 | 0.260 |  |  |  |  |  |  |  |  |  |  |  |  |  |  |  |  |  |  |  |  |  |  |  |  |  |  |  |  |
| **304** | P1765 | PROKKA_01782 | 463 |  |  | AR_NP_115321 | 0.235 |  |  |  |  |  |  |  |  |  |  |  |  |  |  |  |  |  |  |  |  |  |  |  |  |  |  | ARI_16579877 | 0.296 |
| **305** | P1767 | PROKKA_01784 | 246 | VFG0344 | 0.297 |  |  |  |  |  |  |  |  |  |  |  |  |  |  |  |  |  |  |  |  |  |  |  |  |  |  | PAI_CAA21405 | 0.337 |  |  |
| **306** | P1775 | PROKKA_01792 | 393 | VFG1206 | 0.242 |  |  |  |  |  |  |  |  |  |  |  |  |  |  |  |  |  |  |  |  |  |  |  |  |  |  |  |  |  |  |
| **307** | P1779 | PROKKA_01796 | 184 | VFG1601 | 0.299 |  |  |  |  |  |  |  |  |  |  |  |  |  |  |  |  |  |  |  |  |  |  |  |  |  |  | PAI_149774743 | 0.261 |  |  |
| **308** | P1784 | PROKKA_01801 | 216 |  |  | AR_YP_002850060 | 0.336 |  |  |  |  |  |  |  |  |  |  |  |  |  |  |  |  |  |  |  |  |  |  |  |  |  |  |  |  |
| **309** | P1793 | PROKKA_01810 | 433 | VFG1582 | 0.558 |  |  |  |  |  |  |  |  |  |  |  |  |  |  |  |  |  |  |  |  |  |  |  |  |  |  |  |  |  |  |
| **310** | P1810 | PROKKA_01827 | 279 |  |  |  |  |  |  |  |  |  |  |  |  |  |  |  |  |  |  |  |  |  |  |  |  |  |  |  |  | PAI_CAA21372 | 0.245 |  |  |
| **311** | P1811 | PROKKA_01828 | 181 |  |  |  |  |  |  |  |  |  |  |  |  |  |  |  |  |  |  |  |  |  |  |  |  |  |  |  |  |  |  | ARI_18148883 | 0.663 |
| **312** | P1815 | PROKKA_01832 | 201 |  |  |  |  |  |  |  |  |  |  |  |  |  |  |  |  |  |  |  |  |  |  |  |  |  |  |  |  |  |  | ARI_15808710 | 0.308 |
| **313** | P1816 | PROKKA_01833 | 251 |  |  |  |  |  |  |  |  |  |  |  |  |  |  |  |  |  |  |  |  |  |  |  |  |  |  |  |  |  |  | ARI_5360802 | 0.705 |
| **314** | P1817 | PROKKA_01834 | 86 |  |  |  |  |  |  |  |  |  |  |  |  |  |  |  |  |  |  |  |  |  |  |  |  |  |  |  |  |  |  | ARI_28465867 | 0.849 |
| **315** | P1818 | PROKKA_01835 | 355 |  |  |  |  |  |  |  |  |  |  |  |  |  |  |  |  |  |  |  |  |  |  |  |  |  |  |  |  |  |  | ARI_28465866 | 0.783 |
| **316** | P1819 | PROKKA_01836 | 444 |  |  |  |  |  |  |  |  |  |  |  |  |  |  |  |  |  |  |  |  |  |  |  |  |  |  |  |  |  |  | ARI_18148888 | 0.741 |
| **317** | P1820 | PROKKA_01837 | 397 |  |  |  |  |  |  |  |  |  |  |  |  |  |  |  |  |  |  |  |  |  |  |  |  |  |  |  |  |  |  | ARI_24461549 | 0.413 |
| **318** | P1821 | PROKKA_01838 | 182 |  |  |  |  |  |  |  |  |  |  |  |  |  |  |  |  |  |  |  |  |  |  |  |  |  |  | ISArsp6_PEP | 0.409 |  |  | ARI_481190461 | 0.423 |
| **319** | P1823 | PROKKA_01840 | 546 |  |  |  |  |  |  |  |  |  |  |  |  |  |  |  |  |  |  |  |  |  |  |  |  |  |  |  |  |  |  | ARI_14021018 | 0.678 |
| **320** | P1824 | PROKKA_01841 | 132 |  |  |  |  |  |  |  |  |  |  |  |  |  |  |  |  |  |  |  |  |  |  |  |  | Integron_371449816 | 0.364 |  |  |  |  | ARI_14021022 | 0.583 |
| **321** | P1825 | PROKKA_01842 | 121 |  |  |  |  |  |  |  |  |  |  |  |  |  |  |  |  |  |  |  |  |  |  |  |  |  |  |  |  |  |  | ARI_28465872 | 0.537 |
| **322** | P1826 | PROKKA_01843 | 99 |  |  |  |  |  |  |  |  |  |  |  |  |  |  |  |  |  |  |  |  |  |  |  |  |  |  |  |  | PAI_CAA21354 | 0.263 | ARI_24461530 | 0.320 |
| **323** | P1828 | PROKKA_01845 | 204 |  |  |  |  |  |  |  |  |  |  |  |  |  |  |  |  |  |  |  |  |  |  |  |  |  |  |  |  |  |  | ARI_28465869 | 0.941 |
| **324** | P1829 | PROKKA_01846 | 797 |  |  |  |  |  |  |  |  |  |  |  |  |  |  |  |  |  |  |  |  |  |  |  |  |  |  |  |  |  |  | ARI_28465870 | 0.922 |
| **325** | P1830 | PROKKA_01847 | 122 |  |  |  |  |  |  |  |  |  |  |  |  |  |  |  |  |  |  |  |  |  |  |  |  |  |  |  |  |  |  | ARI_28465871 | 0.459 |
| **326** | P1831 | PROKKA_01848 | 129 |  |  |  |  |  |  |  |  |  |  |  |  |  |  |  |  |  |  |  |  |  |  |  |  |  |  | ISArsp6_PEP | 0.328 |  |  |  |  |
| **327** | P1832 | PROKKA_01849 | 131 |  |  |  |  |  |  |  |  |  |  |  |  |  |  |  |  |  |  |  |  |  |  |  |  |  |  |  |  |  |  | ARI_672940410 | 0.336 |
| **328** | P1834 | PROKKA_01851 | 104 |  |  |  |  |  |  |  |  |  |  |  |  |  |  |  |  |  |  |  |  |  |  |  |  |  |  |  |  |  |  | ARI_672940411 | 0.240 |
| **329** | P1839 | PROKKA_01856 | 106 |  |  |  |  |  |  |  |  |  |  |  |  |  |  |  |  |  |  |  |  |  |  |  |  |  |  |  |  |  |  | ARI_672940411 | 0.274 |
| **330** | P1849 | PROKKA_01866 | 224 |  |  |  |  |  |  |  |  |  |  |  |  |  |  |  |  |  |  |  |  |  |  |  |  |  |  | IS_PEP | 0.924 |  |  |  |  |
| **331** | P1854 | PROKKA_01871 | 280 |  |  |  |  |  |  |  |  |  |  |  |  |  |  |  |  |  |  |  |  |  |  |  |  |  |  |  |  | PAI_CAF28565 | 0.309 |  |  |
| **332** | P1858 | PROKKA_01875 | 380 |  |  |  |  |  |  |  |  |  |  |  |  | Prophage_80159731 | 0.529 |  |  |  |  |  |  |  |  |  |  |  |  | ISEfa4_PEP2 | 0.753 |  |  |  |  |
| **333** | P1859 | PROKKA_01876 | 134 |  |  |  |  |  |  |  |  |  |  |  |  |  |  |  |  |  |  |  |  |  |  |  |  |  |  | ISEfa4_PEP | 0.798 |  |  |  |  |
| **334** | P1869 | PROKKA_01886 | 385 | VFG0682 | 0.491 |  |  |  |  |  |  |  |  |  |  |  |  |  |  |  |  |  |  |  |  |  |  |  |  |  |  |  |  |  |  |
| **335** | P1870 | PROKKA_01887 | 150 | VFG0681 | 0.560 |  |  |  |  |  |  |  |  |  |  |  |  |  |  |  |  |  |  |  |  |  |  |  |  |  |  |  |  |  |  |
| **336** | P1871 | PROKKA_01888 | 358 | VFG0680 | 0.271 |  |  |  |  |  |  |  |  |  |  |  |  |  |  |  |  |  |  |  |  |  |  |  |  |  |  |  |  |  |  |
| **337** | P1873 | PROKKA_01890 | 534 | VFG0679 | 0.294 |  |  |  |  |  |  |  |  |  |  |  |  |  |  |  |  |  |  |  |  |  |  |  |  |  |  |  |  |  |  |
| **338** | P1874 | PROKKA_01891 | 238 | VFG0700 | 0.319 |  |  |  |  |  |  |  |  |  |  |  |  |  |  |  |  |  |  |  |  |  |  |  |  |  |  |  |  |  |  |
| **339** | P1883 | PROKKA_01900 | 453 | VFG1583 | 0.358 |  |  |  |  |  |  |  |  |  |  |  |  |  |  |  |  |  |  |  |  |  |  |  |  |  |  |  |  |  |  |
| **340** | P1886 | PROKKA_01903 | 225 | VFG1114 | 0.316 |  |  |  |  |  |  |  |  |  |  |  |  |  |  |  |  |  |  |  |  |  |  |  |  |  |  |  |  |  |  |
| **341** | P1888 | PROKKA_01905 | 291 | VFG1115 | 0.241 |  |  |  |  |  |  |  |  |  |  |  |  |  |  |  |  |  |  |  |  |  |  |  |  |  |  |  |  |  |  |
| **342** | P1889 | PROKKA_01906 | 293 | VFG1109 | 0.270 |  |  |  |  |  |  |  |  |  |  |  |  |  |  |  |  |  |  |  |  |  |  |  |  |  |  |  |  |  |  |
| **343** | P1896 | PROKKA_01913 | 593 | VFG1269 | 0.314 |  |  |  |  |  |  |  |  |  |  |  |  |  |  |  |  |  |  |  |  |  |  |  |  |  |  |  |  |  |  |
| **344** | P1897 | PROKKA_01914 | 592 | VFG0907 | 0.269 |  |  |  |  |  |  |  |  |  |  |  |  |  |  |  |  |  |  |  |  |  |  |  |  |  |  | PAI_CAD33760 | 0.269 |  |  |
| **345** | P1903 | PROKKA_01920 | 291 |  |  |  |  |  |  |  |  |  |  |  |  |  |  |  |  |  |  |  |  |  |  |  |  |  |  |  |  | PAI_CAA21372 | 0.251 |  |  |
| **346** | P1914 | PROKKA_01931 | 391 |  |  |  |  |  |  |  |  |  |  |  |  |  |  |  |  |  |  |  |  |  |  |  |  |  |  |  |  |  |  | ARI_16579875 | 0.762 |
| **347** | P1917 | PROKKA_01934 | 514 |  |  |  |  |  |  |  |  |  |  |  |  |  |  |  |  |  |  |  |  |  |  |  |  |  |  |  |  | PAI_AAO17179 | 0.294 | ARI_12698392 | 0.274 |
| **348** | P1924 | PROKKA_01941 | 473 |  |  |  |  |  |  |  |  |  |  |  |  |  |  |  |  |  |  |  |  |  |  |  |  |  |  |  |  | PAI_AAO17231 | 0.298 |  |  |
| **349** | P1927 | PROKKA_01944 | 253 |  |  | AR_NP_415400 | 0.344 |  |  |  |  |  |  |  |  |  |  |  |  |  |  |  |  |  |  |  |  |  |  |  |  |  |  |  |  |
| **350** | P1928 | PROKKA_01945 | 483 |  |  |  |  |  |  |  |  |  |  |  |  |  |  |  |  |  |  |  |  |  |  |  |  |  |  |  |  |  |  | ARI_409973527 | 0.520 |
| **351** | P1929 | PROKKA_01946 | 794 |  |  |  |  |  |  |  |  |  |  |  |  |  |  |  |  |  |  |  |  |  |  |  |  |  |  |  |  |  |  | ARI_28465870 | 0.270 |
| **352** | P1930 | PROKKA_01947 | 68 |  |  |  |  |  |  |  |  |  |  |  |  |  |  |  |  |  |  |  |  |  |  |  |  |  |  |  |  |  |  | ARI_672940427 | 0.324 |
| **353** | P1939 | PROKKA_01956 | 263 |  |  |  |  |  |  |  |  |  |  |  |  |  |  |  |  |  |  |  |  |  |  |  |  |  |  |  |  | PAI_AAO17195 | 0.274 | ARI_12698385 | 0.304 |
| **354** | P1942 | PROKKA_01959 | 712 |  |  |  |  |  |  |  |  |  |  |  |  |  |  |  |  |  |  |  |  |  |  |  |  |  |  |  |  | PAI_CAI36097 | 0.298 | ARI_24461623 | 0.296 |
| **355** | P1974 | PROKKA_01991 | 270 | VFG1557 | 0.293 |  |  |  |  |  |  |  |  |  |  |  |  |  |  |  |  |  |  |  |  |  |  |  |  |  |  | PAI_CAD33760 | 0.293 |  |  |
| **356** | P1980 | PROKKA_01997 | 348 |  |  |  |  |  |  |  |  |  |  |  |  | Prophage_23455720 | 0.285 |  |  |  |  |  |  |  |  |  |  |  |  |  |  |  |  |  |  |
| **357** | P1981 | PROKKA_01998 | 60 |  |  |  |  |  |  |  |  |  |  |  |  | Prophage_157311142 | 0.283 |  |  |  |  |  |  |  |  |  |  |  |  |  |  |  |  |  |  |
| **358** | P1987 | PROKKA_02004 | 465 |  |  | AR_CAD70268 | 0.510 |  |  |  |  |  |  |  |  |  |  |  |  |  |  |  |  |  |  |  |  |  |  |  |  |  |  |  |  |
| **359** | P2010 | PROKKA_02027 | 298 |  |  | AR_YP_001373621 | 0.292 |  |  |  |  |  |  |  |  |  |  |  |  |  |  |  |  |  |  |  |  |  |  |  |  |  |  |  |  |
| **360** | P2019 | PROKKA_02036 | 234 | VFG1206 | 0.308 | AR_YP_001373621 | 0.316 |  |  |  |  |  |  |  |  |  |  |  |  |  |  |  |  |  |  |  |  |  |  |  |  |  |  |  |  |
| **361** | P2031 | PROKKA_02049 | 206 |  |  |  |  |  |  |  |  |  |  |  |  | Prophage_82701134 | 0.364 |  |  |  |  |  |  |  |  |  |  |  |  |  |  |  |  |  |  |
| **362** | P2042 | PROKKA_02060 | 296 |  |  | AR_ZP_03319325 | 0.348 |  |  |  |  |  |  |  |  |  |  |  |  |  |  |  |  |  |  |  |  |  |  |  |  |  |  |  |  |
| **363** | P2063 | PROKKA_02081 | 267 |  |  | AR_YP_209353 | 0.333 |  |  |  |  |  |  |  |  |  |  |  |  |  |  |  |  |  |  |  |  |  |  |  |  | PAI_331029100 | 0.333 |  |  |
| **364** | P2067 | PROKKA_02085 | 495 | VFG1668 | 0.491 |  |  |  |  |  |  |  |  |  |  |  |  |  |  |  |  |  |  |  |  |  |  |  |  |  |  | PAI_CAD66193 | 0.491 |  |  |
| **365** | P2073 | PROKKA_02100 | 289 |  |  |  |  |  |  |  |  |  |  |  |  |  |  |  |  |  |  |  |  |  |  |  |  |  |  |  |  |  |  | ARI_484356542 | 0.260 |
| **366** | P2087 | PROKKA_02114 | 329 | VFG2197 | 0.266 |  |  |  |  |  |  |  |  |  |  |  |  |  |  |  |  |  |  |  |  |  |  |  |  |  |  |  |  |  |  |
| **367** | P2092 | PROKKA_02119 | 305 |  |  | AR_NP_438601 | 0.252 |  |  |  |  |  |  |  |  |  |  |  |  |  |  |  |  |  |  |  |  |  |  |  |  |  |  |  |  |
| **368** | P2106 | PROKKA_02134 | 263 |  |  |  |  |  |  |  |  |  |  |  |  |  |  |  |  |  |  |  |  |  |  |  |  |  |  |  |  | PAI_AAR03910 | 0.300 |  |  |
| **369** | P2111 | PROKKA_02139 | 104 |  |  |  |  |  |  |  |  |  |  |  |  | Prophage_9630289 | 0.260 |  |  |  |  |  |  |  |  |  |  |  |  |  |  |  |  |  |  |
| **370** | P2119 | PROKKA_02147 | 246 |  |  | AR_ZP_04606269 | 0.264 |  |  |  |  |  |  |  |  |  |  |  |  |  |  |  |  |  |  |  |  |  |  |  |  |  |  |  |  |
| **371** | P2124 | PROKKA_02152 | 161 | VFG0320 | 0.404 |  |  |  |  |  |  |  |  |  |  |  |  |  |  |  |  |  |  |  |  |  |  |  |  |  |  |  |  |  |  |
| **372** | P2137 | PROKKA_02165 | 337 | VFG1398 | 0.258 |  |  |  |  |  |  |  |  |  |  |  |  |  |  |  |  |  |  |  |  |  |  |  |  |  |  |  |  |  |  |
| **373** | P2149 | PROKKA_02177 | 294 | VFG0344 | 0.259 |  |  |  |  |  |  |  |  |  |  |  |  |  |  |  |  |  |  |  |  |  |  |  |  |  |  |  |  |  |  |
| **374** | P2154 | PROKKA_02182 | 534 |  |  | AR_AAC32027 | 0.271 |  |  |  |  |  |  |  |  |  |  |  |  |  |  |  |  |  |  |  |  |  |  |  |  |  |  |  |  |
| **375** | P2161 | PROKKA_02189 | 385 |  |  |  |  |  |  |  |  |  |  |  |  |  |  |  |  |  |  |  |  |  |  |  |  |  |  |  |  |  |  | ARI_16579875 | 0.270 |
| **376** | P2162 | PROKKA_02190 | 357 |  |  | AR_Q93A44 | 0.252 |  |  |  |  |  |  |  |  |  |  |  |  |  |  |  |  |  |  |  |  |  |  |  |  |  |  |  |  |
| **377** | P2164 | PROKKA_02192 | 66 |  |  |  |  |  |  |  |  |  |  |  |  | Prophage_13095918 | 0.636 |  |  |  |  |  |  |  |  |  |  |  |  |  |  | PAI_AAO18076 | 0.545 |  |  |
| **378** | P2166 | PROKKA_02194 | 320 | VFG1330 | 0.255 |  |  |  |  |  |  |  |  |  |  |  |  |  |  |  |  |  |  |  |  |  |  |  |  |  |  |  |  |  |  |
| **379** | P2167 | PROKKA_02195 | 474 |  |  |  |  |  |  |  |  |  |  |  |  |  |  |  |  |  |  |  |  |  |  |  |  |  |  |  |  | PAI_AAO17183 | 0.352 | ARI_12698392 | 0.305 |
| **380** | P2168 | PROKKA_02196 | 150 | VFG1688 | 0.240 |  |  |  |  |  |  |  |  |  |  |  |  |  |  |  |  |  |  |  |  |  |  |  |  |  |  | PAI_CAD66213 | 0.240 |  |  |
| **381** | P2184 | PROKKA_02212 | 452 | VFG1583 | 0.374 |  |  |  |  |  |  |  |  |  |  |  |  |  |  |  |  |  |  |  |  |  |  |  |  |  |  |  |  |  |  |
| **382** | P2189 | PROKKA_02217 | 61 |  |  |  |  |  |  |  |  |  |  |  |  | Prophage_66396175 | 0.377 |  |  |  |  |  |  |  |  |  |  |  |  |  |  |  |  |  |  |
| **383** | P2204 | PROKKA_02240 | 245 |  |  | AR_YP_001373621 | 0.327 |  |  |  |  |  |  |  |  |  |  |  |  |  |  |  |  |  |  |  |  |  |  |  |  |  |  |  |  |
| **384** | P2218 | PROKKA_02254 | 442 |  |  | AR_ZP_03053667 | 0.251 |  |  |  |  |  |  |  |  |  |  |  |  |  |  |  |  |  |  |  |  |  |  |  |  |  |  |  |  |
| **385** | P2219 | PROKKA_02255 | 230 | DBETH_Q897Y4 | 0.283 |  |  |  |  |  |  |  |  |  |  |  |  |  |  |  |  |  |  |  |  |  |  |  |  |  |  |  |  |  |  |
| **386** | P2221 | PROKKA_02257 | 167 |  |  |  |  |  |  |  |  |  |  |  |  | Prophage_156564213 | 0.252 |  |  |  |  |  |  |  |  |  |  |  |  |  |  |  |  |  |  |
| **387** | P2223 | PROKKA_02259 | 103 | VFG1324 | 0.340 |  |  |  |  |  |  |  |  |  |  |  |  |  |  |  |  |  |  |  |  |  |  |  |  |  |  |  |  |  |  |
| **388** | P2236 | PROKKA_02272 | 178 |  |  |  |  |  |  |  |  |  |  |  |  | Prophage_9630137 | 0.247 |  |  |  |  |  |  |  |  |  |  |  |  | ISBce8_PEP2 | 0.292 |  |  |  |  |
| **389** | P2240 | PROKKA_02276 | 538 | VFG0841 | 0.283 |  |  |  |  |  |  |  |  |  |  |  |  |  |  |  |  |  |  |  |  |  |  |  |  |  |  | PAI_CAC43427 | 0.258 |  |  |
| **390** | P2242 | PROKKA_02278 | 257 | VFG2177 | 0.269 |  |  |  |  |  |  |  |  |  |  |  |  |  |  |  |  |  |  |  |  |  |  |  |  |  |  |  |  |  |  |
| **391** | P2249 | PROKKA_02285 | 376 |  |  | AR_ACI02941 | 0.306 |  |  |  |  |  |  |  |  |  |  |  |  |  |  |  |  |  |  |  |  |  |  |  |  |  |  |  |  |
| **392** | P2251 | PROKKA_02287 | 176 |  |  |  |  |  |  |  |  |  |  |  |  |  |  |  |  |  |  |  |  |  |  |  |  |  |  |  |  |  |  | ARI_18148883 | 0.713 |
| **393** | P2257 | PROKKA_02293 | 141 |  |  |  |  |  |  |  |  |  |  |  |  |  |  |  |  |  |  |  |  |  |  |  |  | Integron_374095345 | 0.525 |  |  |  |  | ARI_90265363 | 0.525 |
| **394** | P2267 | PROKKA_02303 | 103 |  |  |  |  |  |  |  |  |  |  |  |  |  |  |  |  |  |  |  |  |  |  |  |  |  |  |  |  |  |  | ARI_18148887 | 0.330 |
| **395** | P2282 | PROKKA_02318 | 284 |  |  |  |  |  |  |  |  |  |  |  |  | Prophage_66395217 | 0.331 |  |  |  |  |  |  |  |  |  |  |  |  |  |  |  |  |  |  |
| **396** | P2287 | PROKKA_02323 | 237 |  |  |  |  |  |  |  |  |  |  |  |  | Prophage_61806373 | 0.316 |  |  |  |  |  |  |  |  |  |  |  |  |  |  |  |  |  |  |
| **397** | P2290 | PROKKA_02326 | 100 |  |  |  |  |  |  |  |  |  |  |  |  |  |  |  |  |  |  |  |  |  |  |  |  |  |  |  |  |  |  | ARI_16579863 | 0.710 |
| **398** | P2300 | PROKKA_02336 | 131 |  |  |  |  |  |  |  |  |  |  |  |  |  |  |  |  |  |  |  |  |  |  |  |  |  |  |  |  |  |  | ARI_229002245 | 0.244 |
| **399** | P2302 | PROKKA_02338 | 146 |  |  |  |  |  |  |  |  |  |  |  |  |  |  |  |  |  |  |  |  |  |  |  |  |  |  |  |  |  |  | ARI_166197593 | 0.911 |
| **400** | P2303 | PROKKA_02339 | 309 |  |  |  |  |  |  |  |  |  |  |  |  |  |  |  |  |  |  |  |  |  |  |  |  |  |  |  |  |  |  | ARI_166197594 | 0.851 |
| **401** | P2304 | PROKKA_02340 | 221 | VFG1297 | 0.394 |  |  |  |  |  |  |  |  |  |  |  |  |  |  |  |  |  |  |  |  |  |  |  |  |  |  |  |  | ARI_506697 | 0.557 |
| **402** | P2305 | PROKKA_02341 | 229 | VFG1298 | 0.633 |  |  |  |  |  |  |  |  |  |  |  |  |  |  |  |  |  |  |  |  |  |  |  |  |  |  |  |  | ARI_506698 | 0.716 |
| **403** | P2306 | PROKKA_02342 | 257 | VFG1299 | 0.584 |  |  |  |  |  |  |  |  |  |  |  |  |  |  |  |  |  |  |  |  |  |  |  |  |  |  |  |  | ARI_506699 | 0.607 |
| **404** | P2307 | PROKKA_02343 | 603 | VFG1300 | 0.720 |  |  |  |  |  |  |  |  |  |  |  |  |  |  |  |  |  |  |  |  |  |  |  |  |  |  |  |  | ARI_506700 | 0.766 |
| **405** | P2317 | PROKKA_02353 | 183 |  |  |  |  |  |  |  |  |  |  |  |  |  |  |  |  |  |  |  |  |  |  |  |  |  |  |  |  |  |  | ARI_481190461 | 0.355 |
| **406** | P2320 | PROKKA_02356 | 202 |  |  |  |  |  |  |  |  |  |  |  |  |  |  |  |  |  |  |  |  |  |  |  |  |  |  |  |  |  |  | ARI_15808710 | 0.337 |
| **407** | P2324 | PROKKA_02360 | 514 |  |  | AR_AAQ10697 | 0.996 |  |  |  |  |  |  |  |  |  |  |  |  |  |  |  |  |  |  |  |  |  |  |  |  |  |  |  |  |
| **408** | P2333 | PROKKA_02369 | 209 | VFG1824 | 0.365 |  |  |  |  |  |  |  |  |  |  |  |  |  |  |  |  |  |  |  |  |  |  |  |  |  |  |  |  |  |  |
| **409** | P2342 | PROKKA_02378 | 240 | VFG1206 | 0.350 |  |  |  |  |  |  |  |  |  |  |  |  |  |  |  |  |  |  |  |  |  |  |  |  |  |  |  |  |  |  |
| **410** | P2360 | PROKKA_02423 | 248 |  |  |  |  |  |  |  |  |  |  |  |  | Prophage_66395003 | 0.340 |  |  |  |  |  |  |  |  |  |  |  |  |  |  |  |  |  |  |
| **411** | P2366 | PROKKA_02429 | 193 |  |  |  |  |  |  |  |  |  |  |  |  | Prophage_47073737 | 0.269 |  |  |  |  |  |  |  |  |  |  |  |  |  |  |  |  |  |  |
| **412** | P2368 | PROKKA_02431 | 454 |  |  |  |  |  |  |  |  |  |  |  |  |  |  |  |  |  |  |  |  |  |  |  |  |  |  |  |  | PAI_CAA21364 | 0.317 |  |  |
| **413** | P2372 | PROKKA_02435 | 326 |  |  |  |  |  |  |  |  |  |  |  |  |  |  |  |  |  |  |  |  |  |  |  |  |  |  |  |  |  |  | ARI_229002247 | 0.850 |
| **414** | P2377 | PROKKA_02440 | 380 | VFG2226 | 0.276 |  |  |  |  |  |  |  |  |  |  |  |  |  |  |  |  |  |  |  |  |  |  |  |  |  |  |  |  | ARI_697403887 | 0.321 |
| **415** | P2378 | PROKKA_02441 | 206 | VFG1336 | 0.291 |  |  |  |  |  |  |  |  |  |  |  |  |  |  |  |  |  |  |  |  |  |  |  |  |  |  |  |  | ARI_697403890 | 0.264 |
| **416** | P2379 | PROKKA_02442 | 197 |  |  |  |  |  |  |  |  |  |  |  |  |  |  |  |  |  |  |  |  |  |  |  |  |  |  |  |  |  |  | ARI_484355524 | 0.452 |
| **417** | P2380 | PROKKA_02443 | 192 |  |  |  |  |  |  |  |  |  |  |  |  |  |  |  |  |  |  |  |  |  |  |  |  |  |  |  |  |  |  | ARI_587656398 | 0.255 |
| **418** | P2384 | PROKKA_02447 | 578 | VFG0907 | 0.348 |  |  |  |  |  |  |  |  |  |  |  |  |  |  |  |  |  |  |  |  |  |  |  |  |  |  |  |  |  |  |
| **419** | P2389 | PROKKA_02452 | 147 | VFG0478 | 0.272 |  |  |  |  |  |  |  |  |  |  |  |  |  |  |  |  |  |  |  |  |  |  |  |  |  |  |  |  |  |  |
| **420** | P2392 | PROKKA_02455 | 51 |  |  |  |  |  |  |  |  |  |  |  |  |  |  |  |  |  |  |  |  |  |  |  |  |  |  | ISSep3_PEP | 0.823 |  |  |  |  |
| **421** | P2394 | PROKKA_02457 | 534 |  |  |  |  |  |  |  |  |  |  |  |  |  |  |  |  |  |  |  |  |  |  |  |  |  |  |  |  |  |  | ARI_484355532 | 0.433 |
| **422** | P2403 | PROKKA_02466 | 259 | VFG0925 | 0.297 | AR_NP_415400 | 0.336 |  |  |  |  |  |  |  |  |  |  |  |  |  |  |  |  |  |  |  |  |  |  |  |  |  |  |  |  |
| **423** | P2406 | PROKKA_02469 | 192 |  |  |  |  |  |  |  |  |  |  |  |  |  |  |  |  |  |  |  |  |  |  |  |  |  |  | ISApl1_PEP | 0.500 |  |  |  |  |
| **424** | P2418 | PROKKA_02481 | 157 |  |  |  |  |  |  |  |  |  |  |  |  |  |  |  |  |  |  |  |  |  |  |  |  |  |  |  |  |  |  | ARI_14021013 | 0.350 |
| **425** | P2421 | PROKKA_02484 | 203 |  |  |  |  |  |  |  |  |  |  |  |  |  |  |  |  |  |  |  |  |  |  |  |  |  |  |  |  |  |  | ARI_15808710 | 0.340 |
| **426** | P2426 | PROKKA_02492 | 250 | VFG1296 | 0.428 |  |  |  |  |  |  |  |  |  |  |  |  |  |  |  |  |  |  |  |  |  |  |  |  |  |  |  |  |  |  |
| **427** | P2427 | PROKKA_02493 | 460 | VFG0168 | 0.215 |  |  |  |  |  |  |  |  |  |  |  |  |  |  |  |  |  |  |  |  |  |  |  |  |  |  |  |  | ARI_12698386 | 0.259 |
| **428** | P2438 | PROKKA_02504 | 333 |  |  |  |  |  |  |  |  |  |  |  |  |  |  |  |  |  |  |  |  |  |  |  |  |  |  |  |  |  |  | ARI_506705 | 0.940 |
| **429** | P2439 | PROKKA_02505 | 424 |  |  |  |  |  |  |  |  |  |  |  |  |  |  |  |  |  |  |  |  |  |  |  |  |  |  |  |  |  |  | ARI_506708 | 0.894 |
| **430** | P2440 | PROKKA_02506 | 250 |  |  | AR_Q07448 | 0.404 |  |  |  |  |  |  |  |  |  |  |  |  |  |  |  |  |  |  |  |  |  |  |  |  |  |  | ARI_28465855 | 0.376 |
| **431** | P2444 | PROKKA_02510 | 207 | VFG1336 | 0.276 |  |  |  |  |  |  |  |  |  |  |  |  |  |  |  |  |  |  |  |  |  |  |  |  |  |  |  |  | ARI_697403906 | 0.203 |
| **432** | P2445 | PROKKA_02511 | 382 | VFG2226 | 0.267 |  |  |  |  |  |  |  |  |  |  |  |  |  |  |  |  |  |  |  |  |  |  |  |  |  |  |  |  | ARI_697403887 | 0.322 |
| **433** | P2448 | PROKKA_02514 | 244 |  |  | AR_YP_271816 | 0.996 |  |  |  |  |  |  |  |  |  |  |  |  |  |  |  |  |  |  |  |  |  |  |  |  |  |  |  |  |
| **434** | P2450 | PROKKA_02516 | 140 |  |  | AR_YP_302016 | 0.864 |  |  |  |  |  |  |  |  |  |  |  |  |  |  |  |  |  |  |  |  |  |  |  |  |  |  |  |  |
| **435** | P2452 | PROKKA_02518 | 226 |  |  |  |  |  |  |  |  |  |  |  |  |  |  |  |  |  |  |  |  |  |  |  |  |  |  | IS231K_PEP3 | 0.434 | PAI_ABP49103 | 0.124 |  |  |
| **436** | P2454 | PROKKA_02520 | 192 |  |  |  |  |  |  |  |  |  |  |  |  |  |  |  |  |  |  |  |  |  |  |  |  |  |  | ISSau2_PEP3 | 0.672 |  |  |  |  |
| **437** | P2455 | PROKKA_02521 | 242 |  |  |  |  |  |  |  |  |  |  |  |  |  |  |  |  |  |  |  |  |  |  |  |  |  |  | ISSau2_PEP | 0.508 |  |  |  |  |
| **438** | P2458 | PROKKA_02524 | 280 |  |  |  |  |  |  |  |  |  |  |  |  |  |  |  |  |  |  |  |  |  |  |  |  |  |  | ISSau4_PEP3 | 0.807 |  |  | ARI_229002231 | 0.825 |
| **439** | P2459 | PROKKA_02525 | 104 |  |  |  |  |  |  |  |  |  |  |  |  |  |  |  |  |  |  |  |  |  |  |  |  |  |  | ISSau4_PEP | 0.760 |  |  |  |  |
| **440** | P2460 | PROKKA_02526 | 224 |  |  |  |  |  |  |  |  |  |  |  |  |  |  |  |  |  |  |  |  |  |  |  |  |  |  |  |  |  |  | ARI_5360868 | 0.995 |
| **Microbial Bioinformatics Group at MML, SJTU** | | | |  |  |  |  |  |  |  |  |  |  |  |  |  |  |  |  |  |  |  |  |  |  |  |  |  |  |  |  |  |  |  |  |

^*^ Data are from the original VRprofile output. **Virulence elements**: **VF**, Virulence Factors; **AR**, Acquired Antibiotic Resistance Determinants; **T3SE** Type III secretion effectors; **T4SE** Type IV secretion effectors; **T6SE** Type VI secretion effectors; **T7SE** Type VII secretion effectors; **ICE**, Integrative and Conjugative Elements; **T3SS** Type III secretion systems; **T6SS** Type VI secretion systems; **T7SS** Type VII secretion systems; **Integron**_Class I integrons; **IS**, insertion sequence elements; **PAI**, Pathogenicity islands; **ARI**, Antibiotic Resistance Islands.
