# Supplementary material for: Staphylococcus arlettae Genomics: Novel Insights on Candidate Antibiotic Resistance and Virulence Genes in an Emerging Opportunistic Pathogen
Source: Microorganisms. 2019 Nov 19;7(11):580. doi: 10.3390/microorganisms7110580 (PMC6920755; doi:10.3390/microorganisms7110580)
Supplement: Supplementary file 1 [file microorganisms-07-00580-s001.zip › Table S6.docx]

**Table S6.** Number of predicted genes correlated to virulence elements identified within species dataset

| Species | Strains | Abbreviation | VF | AR | T1 | T2 | T3 | T4 | Prophage | T5 | T6 | T7 | Integron | IS | PAI | ARI | TOT |
| --- | --- | --- | --- | --- | --- | --- | --- | --- | --- | --- | --- | --- | --- | --- | --- | --- | --- |
| Staphylococcus  arlettae | ***BARI*** | B | 135 | 66 | 3 | 2 | 0 | 0 | 84 | 1 | 2 | 0 | 2 | 15 | 111 | 121 | 542 |
|  | ***BARI1*** | B1 | 145 | 70 | 3 | 2 | 0 | 0 | 84 | 1 | 2 | 0 | 1 | 19 | 109 | 143 | 579 |
|  | ***BARI2*** | B2 | 144 | 69 | 3 | 2 | 0 | 0 | 80 | 1 | 2 | 0 | 1 | 15 | 107 | 135 | 559 |
|  | ***BARI3*** | B3 | 137 | 65 | 3 | 2 | 0 | 0 | 82 | 1 | 2 | 0 | 1 | 14 | 104 | 128 | 539 |
|  | ***NCTC12413*** | TSAR | 143 | 69 | 2 | 2 | 0 | 0 | 151 | 1 | 2 | 0 | 1 | 11 | 116 | 146 | 644 |
|  | ***CDV059*** | AR1 | 146 | 66 | 3 | 2 | 0 | 0 | 78 | 1 | 3 | 0 | 1 | 22 | 107 | 122 | 551 |
|  | ***SNUC4786*** | AR2 | 149 | 68 | 3 | 2 | 1 | 0 | 124 | 1 | 2 | 0 | 1 | 30 | 120 | 156 | 657 |
|  | ***SNUC1715.2*** | AR3 | 147 | 71 | 3 | 2 | 1 | 0 | 121 | 1 | 2 | 0 | 1 | 16 | 117 | 121 | 603 |
|  | ***SNUC4426*** | AR4 | 145 | 67 | 3 | 2 | 1 | 0 | 70 | 1 | 2 | 0 | 1 | 8 | 106 | 115 | 521 |
|  | ***SNUC1330*** | AR5 | 142 | 66 | 3 | 3 | 1 | 0 | 74 | 1 | 2 | 0 | 0 | 17 | 108 | 127 | 544 |
|  | ***SNUC4292*** | AR6 | 145 | 71 | 3 | 2 | 1 | 0 | 85 | 1 | 2 | 0 | 0 | 13 | 115 | 129 | 567 |
|  | ***SNUC3447*** | AR7 | 146 | 70 | 3 | 2 | 1 | 0 | 110 | 1 | 2 | 0 | 0 | 7 | 104 | 114 | 560 |
|  | ***SNUC2101*** | AR8 | 155 | 71 | 3 | 2 | 1 | 0 | 72 | 1 | 2 | 0 | 0 | 13 | 106 | 128 | 554 |
|  | ***SNUC3029*** | AR9 | 147 | 70 | 3 | 2 | 1 | 0 | 81 | 1 | 2 | 0 | 1 | 11 | 116 | 119 | 554 |
|  | ***SNUC1576*** | AR10 | 152 | 74 | 3 | 3 | 1 | 0 | 86 | 1 | 1 | 0 | 1 | 15 | 115 | 122 | 574 |
| Staphylococcus  aureus | ***DSM20714*** | TSA1 | 170 | 47 | 3 | 2 | 1 | 2 | 124 | 1 | 3 | 0 | 1 | 2 | 94 | 87 | 537 |
|  | ***DSM20231*** | TSA2 | 212 | 60 | 3 | 2 | 1 | 2 | 178 | 1 | 4 | 0 | 2 | 11 | 112 | 130 | 718 |
|  | ***RF122*** | SA1 | 213 | 60 | 2 | 2 | 1 | 2 | 181 | 1 | 3 | 0 | 0 | 17 | 129 | 118 | 729 |
|  | ***N315*** | SA2 | 217 | 63 | 2 | 2 | 1 | 2 | 350 | 1 | 3 | 0 | 2 | 17 | 114 | 172 | 946 |
|  | ***JH1*** | SA3 | 230 | 65 | 2 | 2 | 1 | 2 | 164 | 1 | 3 | 0 | 2 | 23 | 128 | 208 | 831 |
|  | ***Mu50*** | SA4 | 229 | 64 | 2 | 2 | 1 | 2 | 232 | 1 | 3 | 0 | 2 | 32 | 145 | 193 | 908 |
|  | ***Mu3*** | SA5 | 231 | 62 | 2 | 2 | 1 | 2 | 232 | 1 | 3 | 0 | 2 | 26 | 145 | 187 | 896 |
|  | ***Newman*** | SA6 | 213 | 60 | 3 | 2 | 1 | 2 | 302 | 1 | 4 | 0 | 2 | 11 | 109 | 124 | 834 |
|  | ***COL*** | SA7 | 208 | 62 | 3 | 2 | 1 | 2 | 170 | 1 | 3 | 0 | 2 | 8 | 133 | 161 | 756 |
|  | ***Gv69*** | SA8 | 209 | 65 | 3 | 2 | 1 | 0 | 355 | 1 | 4 | 0 | 1 | 18 | 123 | 199 | 981 |
|  | ***DAR4145*** | SA9 | 219 | 72 | 3 | 2 | 1 | 0 | 235 | 1 | 3 | 0 | 2 | 10 | 126 | 171 | 845 |
|  | ***ILRI_Eymole1/1*** | SA10 | 216 | 66 | 2 | 2 | 1 | 0 | 328 | 1 | 3 | 0 | 1 | 24 | 159 | 123 | 926 |
|  | ***Mq2T*** | Mq2T | 213 | 64 | 2 | 3 | 1 | 2 | 282 | 1 | 3 | 0 | 2 | 13 | 123 | 132 | 841 |
| Staphylococcus epidermidis | ***ATCC14990*** | TSE | 133 | 56 | 3 | 2 | 0 | 0 | 75 | 2 | 2 | 0 | 0 | 18 | 92 | 161 | 544 |
|  | ***ATCC12228*** | SE1 | 132 | 60 | 3 | 2 | 0 | 0 | 103 | 2 | 2 | 0 | 0 | 27 | 95 | 159 | 585 |
|  | ***PM221*** | SE2 | 129 | 60 | 3 | 2 | 0 | 0 | 185 | 2 | 2 | 0 | 0 | 23 | 101 | 124 | 631 |
|  | ***FDAARGOS_153*** | SE3 | 131 | 58 | 3 | 2 | 0 | 0 | 69 | 2 | 2 | 0 | 0 | 27 | 90 | 120 | 504 |
|  | ***NCTC4133*** | SE4 | 126 | 70 | 2 | 2 | 0 | 0 | 92 | 1 | 3 | 0 | 0 | 3 | 119 | 83 | 501 |
|  | ***W23144*** | SE5 | 134 | 64 | 2 | 2 | 1 | 2 | 104 | 2 | 3 | 0 | 0 | 15 | 99 | 132 | 560 |
|  | ***M0881*** | SE6 | 139 | 64 | 3 | 2 | 0 | 0 | 156 | 2 | 2 | 0 | 0 | 25 | 94 | 146 | 633 |
|  | ***C10C*** | SE7 | 134 | 58 | 3 | 2 | 0 | 0 | 180 | 2 | 2 | 0 | 1 | 11 | 97 | 124 | 614 |
|  | ***VCU037*** | SE8 | 141 | 56 | 2 | 2 | 0 | 0 | 83 | 2 | 2 | 0 | 0 | 21 | 91 | 118 | 518 |
|  | ***IS-250*** | SE9 | 133 | 70 | 2 | 2 | 0 | 0 | 158 | 2 | 2 | 0 | 2 | 48 | 97 | 158 | 674 |
|  | ***VCU111*** | SE10 | 131 | 62 | 3 | 2 | 0 | 0 | 142 | 2 | 2 | 0 | 2 | 18 | 93 | 140 | 597 |
| Staphylococcus haemolyticus | ***NCTC11042*** | TSH | 140 | 56 | 1 | 2 | 1 | 0 | 145 | 1 | 2 | 0 | 0 | 28 | 105 | 141 | 622 |
|  | ***JCSC1435*** | SH1 | 140 | 67 | 1 | 2 | 1 | 0 | 198 | 1 | 2 | 0 | 0 | 44 | 110 | 193 | 759 |
|  | ***S167*** | SH2 | 136 | 57 | 1 | 2 | 1 | 0 | 138 | 1 | 2 | 0 | 0 | 22 | 108 | 137 | 605 |
|  | ***83131°*** | SH3 | 134 | 63 | 1 | 2 | 1 | 0 | 142 | 1 | 2 | 0 | 0 | 26 | 99 | 162 | 633 |
|  | ***SGAir0252*** | SH4 | 139 | 60 | 1 | 2 | 1 | 0 | 159 | 1 | 2 | 0 | 2 | 23 | 107 | 153 | 650 |
|  | ***FDAARGOS_517*** | SH5 | 133 | 64 | 0 | 2 | 1 | 0 | 128 | 1 | 2 | 0 | 0 | 12 | 102 | 147 | 592 |
|  | ***DNF00585*** | SH6 | 110 | 53 | 1 | 2 | 1 | 0 | 135 | 1 | 2 | 0 | 0 | 4 | 102 | 101 | 512 |
|  | ***1Ht3*** | SH7 | 126 | 55 | 1 | 2 | 1 | 0 | 95 | 1 | 2 | 0 | 0 | 24 | 102 | 127 | 536 |
|  | ***MTCC3383*** | SH8 | 140 | 55 | 1 | 2 | 1 | 0 | 122 | 1 | 2 | 0 | 0 | 20 | 106 | 134 | 584 |
|  | ***FDAARGOS_130*** | SH9 | 135 | 65 | 1 | 2 | 0 | 0 | 143 | 2 | 2 | 0 | 1 | 24 | 103 | 182 | 660 |
|  | ***864-1*** | SH10 | 137 | 59 | 1 | 2 | 1 | 0 | 118 | 1 | 2 | 0 | 2 | 14 | 108 | 128 | 573 |
| Staphylococcus saprophyticus | ***CCUG38042*** | TSS1 | 135 | 65 | 4 | 2 | 1 | 0 | 178 | 2 | 2 | 0 | 1 | 21 | 111 | 109 | 631 |
|  | ***ATCC15305*** | TSS2 | 143 | 70 | 4 | 2 | 1 | 0 | 82 | 2 | 2 | 0 | 1 | 20 | 108 | 118 | 553 |
|  | ***82C*** | SS1 | 135 | 82 | 2 | 2 | 1 | 0 | 150 | 1 | 2 | 0 | 1 | 25 | 106 | 145 | 652 |
|  | ***FDAARGOS_137*** | SS2 | 127 | 63 | 4 | 2 | 1 | 0 | 120 | 2 | 2 | 0 | 1 | 14 | 102 | 91 | 529 |
|  | ***FDAARGOS_366*** | SS3 | 132 | 65 | 4 | 2 | 1 | 0 | 90 | 2 | 2 | 0 | 1 | 16 | 104 | 109 | 528 |
|  | ***FDAARGOS_335*** | SS4 | 129 | 65 | 4 | 2 | 1 | 0 | 84 | 2 | 2 | 0 | 1 | 17 | 105 | 129 | 541 |
|  | ***FDAARGOS_168*** | SS5 | 127 | 66 | 4 | 2 | 1 | 0 | 159 | 2 | 2 | 0 | 2 | 15 | 115 | 98 | 593 |
|  | ***NCTC7666*** | SS6 | 140 | 71 | 3 | 2 | 1 | 0 | 81 | 2 | 2 | 0 | 1 | 19 | 113 | 121 | 556 |
|  | ***DPC5671*** | SS7 | 135 | 61 | 4 | 2 | 1 | 0 | 166 | 2 | 3 | 0 | 1 | 10 | 105 | 126 | 616 |
|  | ***725A-RS6*** | SS8 | 129 | 65 | 4 | 2 | 1 | 0 | 94 | 2 | 2 | 0 | 1 | 16 | 112 | 104 | 532 |
|  | ***AG1*** | SS9 | 134 | 66 | 4 | 2 | 1 | 0 | 117 | 2 | 2 | 0 | 1 | 14 | 102 | 98 | 543 |
|  | ***SS116*** | SS10 | 135 | 74 | 4 | 2 | 1 | 0 | 130 | 2 | 2 | 0 | 1 | 16 | 112 | 118 | 597 |
| Staphylococcus  kloosii | ***ATCC43959*** | TSK | 144 | 71 | 2 | 2 | 0 | 0 | 159 | 1 | 2 | 2 | 1 | 9 | 115 | 110 | 618 |
|  | ***CNV2*** | K1 | 164 | 74 | 3 | 2 | 0 | 0 | 110 | 1 | 2 | 0 | 1 | 9 | 112 | 110 | 588 |
|  | ***SNUC4696*** | K2 | 154 | 74 | 2 | 2 | 0 | 0 | 128 | 1 | 2 | 0 | 1 | 19 | 122 | 141 | 646 |
|  | ***NCTC12415*** | K3 | 145 | 7 | 1 | 2 | 0 | 0 | 165 | 1 | 2 | 0 | 1 | 10 | 114 | 110 | 558 |
|  | ***NCTC 12415*** | K4 | 146 | 71 | 2 | 2 | 0 | 0 | 160 | 1 | 2 | 0 | 1 | 9 | 115 | 109 | 618 |
| Staphylococcus  cohnii | ***NCTC11041*** | TSC1* | 134 | 67 | 2 | 2 | 1 | 0 | 132 | 1 | 2 | 0 | 2 | 55 | 105 | 140 | 643 |
|  | ***DSM6718*** | TSC2 | 129 | 62 | 3 | 2 | 1 | 0 | 127 | 1 | 2 | 0 | 3 | 16 | 109 | 103 | 558 |
|  | ***C*** | C | 133 | 64 | 3 | 2 | 1 | 0 | 96 | 1 | 2 | 0 | 2 | 13 | 107 | 101 | 525 |
|  | ***532*** | C1 | 128 | 67 | 3 | 2 | 1 | 0 | 150 | 1 | 2 | 0 | 2 | 29 | 101 | 148 | 634 |
|  | ***FDAARGOS_334*** | C2 | 128 | 62 | 3 | 2 | 1 | 0 | 83 | 1 | 2 | 0 | 2 | 10 | 111 | 122 | 527 |
|  | ***FDAARGOS_538*** | C3 | 126 | 63 | 2 | 2 | 1 | 0 | 126 | 1 | 2 | 0 | 2 | 62 | 103 | 129 | 619 |
|  | ***57*** | C4 | 129 | 72 | 3 | 2 | 1 | 0 | 147 | 1 | 2 | 0 | 2 | 35 | 103 | 162 | 659 |
|  | ***G22B2*** | C5 | 134 | 73 | 3 | 2 | 1 | 0 | 158 | 1 | 2 | 0 | 2 | 15 | 109 | 130 | 630 |
|  | ***H62*** | C6 | 123 | 68 | 3 | 2 | 1 | 0 | 124 | 1 | 2 | 0 | 3 | 23 | 104 | 126 | 580 |
|  | ***MF1844*** | C7 | 128 | 61 | 3 | 1 | 1 | 0 | 80 | 1 | 2 | 0 | 2 | 4 | 104 | 94 | 481 |
|  | ***SE4.1*** | C8 | 129 | 62 | 3 | 2 | 2 | 0 | 85 | 1 | 2 | 0 | 3 | 10 | 110 | 124 | 533 |
|  | ***SE3.10*** | C9 | 128 | 62 | 3 | 2 | 2 | 0 | 85 | 1 | 2 | 0 | 3 | 9 | 108 | 123 | 528 |
|  | ***SE4.2*** | C10 | 130 | 62 | 3 | 2 | 2 | 0 | 85 | 1 | 2 | 0 | 3 | 10 | 111 | 122 | 533 |
| Staphylococcus auricularis | ***NCTC12101*** | TSAU* | 123 | 58 | 2 | 2 | 0 | 0 | 90 | 2 | 2 | 0 | 0 | 2 | 84 | 88 | 453 |
|  | ***SNUC3034*** | SAU1 | 122 | 63 | 2 | 2 | 0 | 0 | 104 | 2 | 2 | 0 | 0 | 6 | 82 | 115 | 500 |
|  | ***SNUC993*** | SAU2 | 129 | 61 | 2 | 2 | 0 | 0 | 109 | 2 | 2 | 0 | 0 | 12 | 85 | 105 | 509 |
|  | ***NCTC 12101*** | SAU3 | 119 | 56 | 2 | 2 | 0 | 0 | 86 | 2 | 2 | 0 | 0 | 5 | 78 | 79 | 431 |
| Staphylococcus  hyicus | ***ATCC11249*** | TSHY | 155 | 53 | 3 | 2 | 2 | 0 | 120 | 2 | 2 | 0 | 1 | 6 | 95 | 97 | 538 |
|  | ***NCTC10350*** | HY1 | 158 | 52 | 2 | 3 | 2 | 0 | 123 | 2 | 2 | 0 | 1 | 9 | 94 | 99 | 547 |
|  | ***NCTC7944*** | HY2 | 154 | 54 | 1 | 1 | 0 | 0 | 156 | 2 | 4 | 0 | 1 | 2 | 104 | 107 | 586 |
|  | ***SNUC 5426*** | HY3 | 154 | 52 | 3 | 3 | 2 | 0 | 164 | 2 | 3 | 0 | 1 | 12 | 89 | 96 | 581 |
|  | ***SNUC4992*** | HY4 | 155 | 52 | 3 | 3 | 2 | 0 | 169 | 2 | 2 | 0 | 1 | 13 | 90 | 97 | 589 |
| Staphylococcus chromogenes | ***NCTC 10530*** | TSCH | 134 | 42 | 3 | 3 | 1 | 0 | 110 | 2 | 2 | 0 | 1 | 5 | 91 | 78 | 472 |
|  | ***MU 970*** | CH1 | 137 | 47 | 4 | 3 | 1 | 0 | 118 | 2 | 2 | 0 | 0 | 7 | 91 | 102 | 514 |
|  | ***SNUC 4584*** | CH2 | 134 | 54 | 4 | 3 | 1 | 0 | 117 | 2 | 2 | 0 | 0 | 6 | 90 | 92 | 505 |
|  | ***SNUC 5997*** | CH3 | 136 | 44 | 4 | 3 | 1 | 0 | 114 | 2 | 2 | 0 | 0 | 9 | 91 | 73 | 479 |
|  | ***SNUC5084*** | CH4 | 136 | 52 | 4 | 3 | 1 | 0 | 126 | 2 | 2 | 0 | 0 | 6 | 98 | 98 | 528 |
| Staphylococcus  agnetis | ***DSM 23656*** | TSAG | 154 | 65 | 3 | 3 | 1 | 0 | 185 | 2 | 2 | 0 | 0 | 29 | 98 | 109 | 651 |
|  | ***908*** | AG1 | 156 | 56 | 2 | 3 | 2 | 0 | 169 | 2 | 2 | 0 | 0 | 12 | 94 | 118 | 616 |
|  | ***CBMRN20813338*** | AG2 | 147 | 63 | 2 | 3 | 1 | 0 | 179 | 1 | 2 | 0 | 0 | 19 | 94 | 82 | 593 |
|  | ***722_260714_1_8_heart*** | AG3 | 143 | 63 | 2 | 3 | 2 | 0 | 172 | 1 | 2 | 0 | 0 | 19 | 91 | 122 | 620 |
|  | ***722_230714_2_5_spleen*** | AG4 | 144 | 62 | 2 | 3 | 2 | 0 | 172 | 1 | 2 | 0 | 0 | 18 | 91 | 122 | 619 |
| Staphylococcus  felis | ***DSM 7377*** | TSF | 161 | 65 | 2 | 2 | 0 | 0 | 91 | 2 | 2 | 0 | 0 | 16 | 96 | 75 | 512 |
|  | ***ATCC 49168*** | F1 | 159 | 66 | 2 | 2 | 0 | 0 | 91 | 2 | 2 | 0 | 0 | 35 | 100 | 74 | 533 |
|  | ***F19*** | F2 | 154 | 66 | 2 | 2 | 0 | 0 | 118 | 2 | 2 | 0 | 0 | 16 | 97 | 79 | 538 |
|  | ***F17*** | F3 | 151 | 67 | 2 | 2 | 0 | 0 | 130 | 2 | 2 | 0 | 0 | 25 | 94 | 90 | 565 |
|  | ***F4*** | F4 | 149 | 63 | 2 | 2 | 0 | 0 | 121 | 2 | 2 | 0 | 0 | 22 | 98 | 87 | 548 |
| Staphylococcus simulans | ***NCTC 11046*** | TSSI | 156 | 57 | 1 | 1 | 0 | 0 | 180 | 2 | 3 | 0 | 1 | 15 | 108 | 137 | 661 |
|  | ***FDAARGOS_124*** | SI1 | 152 | 52 | 1 | 1 | 0 | 0 | 90 | 2 | 3 | 0 | 1 | 12 | 108 | 126 | 548 |
|  | ***FDAARGOS_383*** | SI2 | 161 | 52 | 1 | 1 | 0 | 0 | 146 | 2 | 2 | 0 | 1 | 30 | 107 | 135 | 638 |
|  | ***MR3*** | SI3 | 162 | 59 | 1 | 1 | 1 | 0 | 138 | 2 | 3 | 0 | 1 | 25 | 119 | 120 | 632 |
|  | ***MR4*** | SI4 | 161 | 59 | 1 | 1 | 1 | 0 | 138 | 2 | 3 | 0 | 1 | 26 | 119 | 118 | 630 |
|  | ***MR2*** | SI5 | 166 | 59 | 2 | 1 | 1 | 0 | 138 | 2 | 3 | 0 | 1 | 26 | 120 | 120 | 639 |
|  | ***MR1*** | SI6 | 162 | 59 | 1 | 1 | 1 | 0 | 137 | 2 | 3 | 0 | 1 | 25 | 118 | 121 | 631 |
|  | ***ACS-120-V-Sch1*** | SI7 | 166 | 54 | 1 | 1 | 0 | 0 | 144 | 2 | 3 | 0 | 1 | 18 | 116 | 98 | 604 |
|  | ***MJR7712*** | SI8 | 165 | 57 | 1 | 1 | 0 | 0 | 198 | 2 | 3 | 0 | 1 | 8 | 117 | 112 | 665 |
|  | ***UMC-CNS-990*** | SI9 | 160 | 59 | 1 | 1 | 0 | 0 | 199 | 2 | 3 | 0 | 1 | 19 | 118 | 115 | 678 |
|  | ***SNUC 4566*** | SI10 | 161 | 55 | 2 | 1 | 0 | 0 | 167 | 2 | 3 | 0 | 1 | 13 | 109 | 108 | 622 |
| Staphylococcus  sciuri | ***NCTC12103*** | TSSC | 145 | 68 | 3 | 3 | 2 | 0 | 132 | 2 | 2 | 0 | 0 | 13 | 115 | 88 | 573 |
|  | ***FDAARGOS_285*** | SC1 | 161 | 69 | 3 | 3 | 2 | 0 | 100 | 2 | 2 | 0 | 0 | 8 | 128 | 90 | 568 |
|  | ***NS202*** | SC2 | 157 | 67 | 3 | 3 | 3 | 0 | 115 | 2 | 2 | 0 | 0 | 9 | 125 | 89 | 575 |
|  | ***NS53*** | SC3 | 155 | 68 | 3 | 3 | 1 | 0 | 203 | 2 | 2 | 0 | 0 | 11 | 139 | 101 | 688 |
|  | ***RSA37*** | SC4 | 154 | 68 | 3 | 3 | 1 | 0 | 184 | 2 | 2 | 0 | 0 | 10 | 135 | 100 | 662 |
|  | ***NS1*** | SC5 | 155 | 68 | 3 | 3 | 1 | 0 | 232 | 2 | 2 | 0 | 0 | 10 | 140 | 101 | 717 |
|  | ***NS36*** | SC6 | 154 | 69 | 3 | 3 | 1 | 0 | 173 | 2 | 2 | 0 | 0 | 11 | 136 | 102 | 656 |
|  | ***NS112*** | SC7 | 154 | 68 | 3 | 3 | 1 | 0 | 175 | 2 | 2 | 0 | 0 | 10 | 137 | 103 | 658 |
|  | ***NS44*** | SC8 | 158 | 71 | 3 | 3 | 1 | 0 | 173 | 2 | 2 | 0 | 0 | 10 | 137 | 101 | 661 |
|  | ***SAP15-1*** | SC9 | 163 | 71 | 3 | 3 | 3 | 0 | 114 | 2 | 2 | 0 | 0 | 8 | 129 | 107 | 605 |
|  | ***P575*** | SC10 | 156 | 71 | 3 | 3 | 3 | 0 | 126 | 2 | 2 | 0 | 0 | 10 | 121 | 89 | 586 |
| Bacillus  subtilis | ***KCTC 13429*** | TSBS1 | 267 | 98 | 4 | 4 | 1 | 1 | 187 | 3 | 2 | 3 | 3 | 11 | 187 | 180 | 951 |
|  | ***TU-B-10*** | TSBS2 | 261 | 92 | 3 | 4 | 2 | 1 | 194 | 3 | 2 | 2 | 3 | 14 | 184 | 176 | 941 |
|  | ***NCIB 3610*** | TSBS3 | 264 | 102 | 4 | 4 | 1 | 1 | 385 | 3 | 2 | 3 | 2 | 8 | 188 | 174 | 1141 |
|  | ***168*** | BS1 | 261 | 101 | 4 | 4 | 1 | 1 | 349 | 3 | 2 | 3 | 2 | 7 | 186 | 174 | 1098 |
|  | ***W23*** | BS2 | 259 | 94 | 3 | 4 | 1 | 1 | 175 | 4 | 2 | 3 | 2 | 31 | 185 | 167 | 931 |
|  | ***BSn5*** | BS3 | 271 | 103 | 4 | 4 | 1 | 1 | 198 | 3 | 2 | 3 | 2 | 6 | 179 | 172 | 949 |
|  | ***BEST195*** | BS4 | 259 | 93 | 3 | 4 | 1 | 1 | 231 | 3 | 1 | 3 | 1 | 69 | 174 | 160 | 1003 |
|  | ***RO-NN-1*** | BS5 | 273 | 91 | 3 | 5 | 1 | 1 | 150 | 3 | 2 | 3 | 2 | 17 | 178 | 157 | 886 |
|  | ***QB928*** | BS6 | 258 | 97 | 3 | 4 | 1 | 1 | 318 | 3 | 2 | 3 | 2 | 7 | 181 | 166 | 1046 |
|  | ***BSP1*** | BS7 | 272 | 95 | 4 | 3 | 1 | 1 | 146 | 3 | 2 | 2 | 2 | 12 | 183 | 164 | 890 |
|  | ***XF-1*** | BS8 | 255 | 97 | 4 | 3 | 1 | 1 | 156 | 3 | 2 | 3 | 1 | 6 | 175 | 155 | 862 |
|  | ***6051-HGW*** | BS9 | 264 | 102 | 4 | 4 | 1 | 1 | 351 | 3 | 2 | 3 | 2 | 7 | 187 | 177 | 1108 |
|  | ***BAB-1*** | BS10 | 272 | 99 | 4 | 3 | 1 | 1 | 144 | 3 | 2 | 3 | 2 | 8 | 185 | 163 | 890 |
| Macrococcus caseolyticus | ***DSM 20597*** | TSMC | 124 | 54 | 2 | 3 | 1 | 0 | 133 | 1 | 3 | 0 | 0 | 18 | 101 | 94 | 534 |
|  | ***JCSC5402*** | MC1 | 116 | 54 | 2 | 3 | 0 | 0 | 133 | 1 | 5 | 0 | 1 | 20 | 87 | 95 | 517 |
|  | ***IMD0819*** | MC2 | 122 | 55 | 2 | 4 | 1 | 0 | 156 | 1 | 3 | 0 | 0 | 61 | 100 | 114 | 619 |
|  | ***CCM7927*** | MC3 | 114 | 54 | 2 | 3 | 1 | 0 | 88 | 1 | 4 | 0 | 1 | 20 | 86 | 102 | 476 |
|  | ***5815_BC85*** | MC4 | 118 | 56 | 2 | 4 | 0 | 0 | 142 | 1 | 4 | 0 | 0 | 51 | 99 | 108 | 585 |
|  | ***5814_BC75*** | MC5 | 118 | 54 | 2 | 3 | 0 | 0 | 93 | 1 | 3 | 0 | 0 | 25 | 97 | 103 | 499 |
|  | ***5813_BC74*** | MC6 | 120 | 58 | 2 | 3 | 0 | 0 | 146 | 1 | 3 | 0 | 1 | 51 | 99 | 101 | 585 |
|  | ***5804_BC29*** | MC7 | 119 | 54 | 2 | 3 | 1 | 0 | 141 | 1 | 3 | 0 | 1 | 26 | 100 | 104 | 555 |
|  | ***5798_EF375*** | MC8 | 128 | 56 | 2 | 3 | 0 | 0 | 120 | 1 | 3 | 0 | 1 | 21 | 107 | 105 | 547 |
|  | ***5795_EF335*** | MC9 | 123 | 58 | 2 | 3 | 0 | 0 | 132 | 1 | 5 | 0 | 1 | 28 | 100 | 110 | 563 |
|  | ***5794_EF323*** | MC10 | 117 | 55 | 2 | 4 | 0 | 0 | 134 | 1 | 4 | 0 | 0 | 51 | 97 | 107 | 572 |

**Virulence elements**: **VF**, Virulence Factors; **AR**, Acquired Antibiotic Resistance Determinants; **T1**, T3SE_Type III secretion effectors; **T2**, T4SE_Type IV secretion effectors; **T3** ,T6SE_Type VI secretion effectors; **T4**, T7SE_ Type VII secretion effectors; **T5**,T3SS_Type III secretion systems; **T6**, T6SS_Type VI secretion systems; **T7**,T7SS_ Type VII secretion systems; **Int**, Integron_Class I integrons; **IS**, insertion sequence elements; **PAI**, Pathogenicity islands; **ARI**, Antibiotic Resistance Islands.
