# Supplementary material for: Staphylococcus arlettae Genomics: Novel Insights on Candidate Antibiotic Resistance and Virulence Genes in an Emerging Opportunistic Pathogen
Source: Microorganisms. 2019 Nov 19;7(11):580. doi: 10.3390/microorganisms7110580 (PMC6920755; doi:10.3390/microorganisms7110580)
Supplement: Supplementary file 1 [file microorganisms-07-00580-s001.zip › Table S7.docx]

**Table S7.** Distribution of putative genes related to virulence factors identified within species dataset.

| Genes related to virulence factors | *Staphylococcus* species^*^ | | | | | | | | | | | | | | Outgroup species | |
| --- | --- | --- | --- | --- | --- | --- | --- | --- | --- | --- | --- | --- | --- | --- | --- | --- |
|  | **SAR** | **SA** | **SAG** | **SAU** | **SC** | **SCH** | **SE** | **SF** | **SH** | **SHY** | **SK** | **SS** | **SSC** | **SI** | **BS** | **MC** |
| *acpXL* | + |  |  |  |  |  |  |  |  |  |  |  |  |  | + |  |
| *Allantoin utilization(Klebsiella)* | + |  |  |  | + |  |  |  |  | + |  |  | + | + |  |  |
| *atl* |  | + |  |  | + |  | + |  | + |  |  | + |  |  |  |  |
| *bopD* |  |  |  |  |  |  |  |  |  |  |  |  |  |  |  | + |
| *BopD(Enterococcus)* |  |  |  |  |  |  |  |  |  |  |  |  |  |  |  | + |
| *capA* |  |  |  |  |  |  |  |  |  |  |  |  |  |  | + |  |
| *capB* | + |  | + | + | + | + | + |  | + | + | + | + |  | + | + |  |
| *capC* | + |  | + | + | + | + | + |  | + | + | + | + |  | + | + |  |
| *capD* |  |  |  |  |  |  |  |  |  |  |  |  |  |  | + |  |
| *Capsule(Acinetobacter)* | + |  |  |  |  |  |  |  | + | + | + |  |  | + | + |  |
| *Capsule(Enterococcus)* | + |  |  | + |  |  |  |  |  |  |  |  |  |  | + |  |
| *Capsule(Klebsiella)* |  |  |  |  |  |  |  |  |  |  |  |  |  |  | + |  |
| *cbrB* |  |  |  |  |  |  |  |  |  |  |  |  |  |  | + |  |
| *cbrD* |  |  |  |  |  |  |  |  |  |  |  |  |  |  | + |  |
| *cdsN* |  |  |  |  |  |  |  |  |  |  |  |  |  |  | + |  |
| *cheY* |  |  |  |  |  |  |  |  |  |  |  |  |  |  | + |  |
| *clfA* |  | + |  | + |  | + | + |  | + | + | + |  |  |  |  |  |
| *clfB* |  | + |  | + | + | + |  | + | + |  | + | + | + | + |  |  |
| *cna* |  | + | + |  |  |  |  |  |  | + |  |  |  |  |  |  |
| *coa* |  | + |  |  |  |  |  |  |  |  |  |  |  |  |  |  |
| *cpsA* |  |  |  |  |  |  |  |  |  |  |  |  |  |  |  | + |
| *csrA* |  |  |  |  |  |  |  |  |  |  |  |  |  |  | + |  |
| *ctpV* |  |  |  |  |  |  |  |  |  |  |  |  |  |  | + |  |
| *cylL-l* |  |  |  |  |  |  |  |  |  |  |  |  |  | + |  |  |
| *cylR2* | + |  |  | + | + |  |  | + | + |  |  |  |  | + |  | + |
| *cysC1* |  |  | + |  |  | + |  |  |  | + |  |  |  | + |  |  |
| *dltA* |  |  |  |  |  |  |  |  |  |  |  |  |  |  | + |  |
| *eap/map* |  | + |  |  |  |  |  |  |  |  |  |  |  |  |  |  |
| *ebh* |  | + |  |  |  |  | + |  |  |  |  |  |  |  |  |  |
| *ebp* |  | + |  |  | + |  | + |  | + |  | + | + |  |  |  |  |
| *efb* |  | + | + |  |  |  |  |  |  |  |  |  |  |  |  |  |
| *eno* | + |  |  |  |  |  | + |  |  |  |  |  |  |  |  |  |
| *Ent siderophore(Klebsiella)* |  |  |  |  |  |  |  |  |  |  |  |  |  |  | + |  |
| *entB* |  |  |  |  |  |  |  |  |  |  |  |  |  |  | + |  |
| *esaA* | + | + | + |  |  |  | + |  |  |  |  |  |  | + |  |  |
| *esaB* | + | + | + |  |  |  | + |  |  | + |  |  |  | + |  |  |
| *esaC* |  | + |  |  |  |  | + |  |  |  |  |  |  |  |  |  |
| *essA* | + | + |  |  |  |  | + |  |  |  |  |  |  |  |  |  |
| *essB* | + | + | + |  |  |  | + |  |  | + |  |  |  | + |  |  |
| *essC* | + | + | + |  |  |  | + |  |  | + |  |  |  | + | + |  |
| *esxA* | + | + | + |  |  |  | + |  |  | + |  |  |  | + |  |  |
| *esxB* |  | + |  |  |  |  | + |  |  |  |  |  |  |  |  |  |
| *eta* |  | + | + |  |  |  |  |  |  | + |  |  |  |  |  |  |
| *fbpA* |  |  |  |  |  |  |  |  |  |  |  |  |  |  | + |  |
| *fliE* |  |  |  |  |  |  | + |  |  |  |  |  |  |  |  |  |
| *fliP* |  |  |  |  |  |  |  |  |  |  |  |  |  |  | + |  |
| *flmH* | + |  |  |  |  |  |  |  |  |  |  |  |  |  |  |  |
| *fnbA* |  | + | + |  |  | + |  |  |  | + |  | + |  |  |  |  |
| *fnbB* |  | + | + |  |  |  |  | + |  | + |  |  |  |  |  |  |
| *galE* | + |  |  | + | + |  |  | + |  | + | + | + |  | + | + |  |
| *galU* |  |  |  |  | + |  |  |  |  |  |  |  |  |  | + |  |
| *geh* |  | + | + |  |  | + | + | + |  | + |  | + |  | + |  |  |
| *gnd* | + |  |  |  |  |  |  |  |  |  |  |  |  |  | + |  |
| *groEL* | + |  |  |  |  |  |  |  |  |  |  |  |  |  |  |  |
| *gtaB* | + |  |  |  |  |  |  | + |  |  |  | + |  |  | + | + |
| *Hemolysin* |  |  |  |  |  |  |  |  |  |  |  |  |  |  | + |  |
| *Hemolysin (Clostr.)* |  |  |  |  |  |  |  |  |  |  |  |  |  |  | + | + |
| *hlb* |  |  | + |  |  | + | + | + |  | + |  |  |  |  |  |  |
| *hld* |  | + |  | + |  |  | + | + |  |  |  |  |  | + |  |  |
| *hlgA* |  | + |  |  |  |  |  |  |  |  |  |  |  |  |  |  |
| *hlgB* |  | + |  |  |  |  |  |  |  |  |  |  |  |  |  |  |
| *hlgC* |  | + |  |  |  |  |  |  |  |  |  |  |  |  |  |  |
| *hly/hla* |  | + |  |  |  |  |  |  |  |  |  |  |  |  |  |  |
| *hlyIII* |  |  |  |  |  |  |  |  |  |  |  |  |  |  | + |  |
| *hysA* |  | + | + |  |  |  |  |  |  | + |  |  |  |  |  |  |
| *icaA* | + | + |  |  | + |  | + |  |  |  | + | + | + | + |  | + |
| *icaB* | + | + |  |  | + |  | + |  |  |  | + | + | + | + |  |  |
| *icaC* | + | + |  |  | + |  | + |  |  |  | + | + | + | + |  |  |
| *icaD* |  | + |  |  |  |  | + |  |  |  | + |  |  |  |  |  |
| *icaR* |  | + |  |  |  |  | + |  |  |  | + | + |  |  |  |  |
| *katA* | + |  |  |  |  |  |  |  |  |  |  |  |  |  | + |  |
| *lgt* | + |  | + |  |  | + |  | + |  | + |  | + | + |  | + | + |
| *lip* | + | + |  |  | + | + | + | + | + |  | + | + |  |  |  |  |
| *lisR* | + |  | + |  |  | + |  |  |  | + |  |  | + |  |  | + |
| *lpeA* | + |  |  | + |  |  |  |  |  |  |  |  |  |  |  |  |
| *lplA1* | + |  |  |  |  |  |  |  |  |  |  |  |  |  | + | + |
| *LPS O-antigen (P. aeruginosa)* | + |  |  |  | + |  |  |  |  |  |  |  |  | + | + | + |
| *LPS rfb locus(Klebsiella)* | + |  |  |  |  |  |  |  |  |  | + |  |  |  |  |  |
| *lspA* | + |  | + |  |  |  |  |  |  | + | + |  |  | + | + |  |
| *lukD* |  | + |  |  |  |  |  |  |  |  |  |  |  |  |  |  |
| *lukE* |  | + |  |  |  |  |  |  |  |  |  |  |  |  |  |  |
| *lukM* |  | + |  |  |  |  |  |  |  |  |  |  |  |  |  |  |
| *lukF-PV* |  | + |  |  |  |  |  |  |  |  |  |  |  |  |  |  |
| *lukS-PV* |  | + |  |  |  |  |  |  |  |  |  |  |  |  |  |  |
| *lytR* |  |  |  |  |  |  |  |  |  |  |  |  |  |  | + |  |
| *manA* |  |  |  |  |  |  |  |  |  |  |  | + |  |  | + |  |
| *mbtH* |  |  |  |  |  |  |  |  |  |  |  |  |  |  | + |  |
| *narG* |  |  |  |  |  |  |  |  |  |  |  |  |  |  | + |  |
| *narH* |  |  |  |  |  |  |  |  |  |  |  | + |  |  | + |  |
| *ndk* | + |  |  |  |  |  |  |  |  |  |  |  | + |  | + | + |
| *nuc* | + |  | + | + | + | + | + | + | + | + | + | + |  | + |  |  |
| *oatA* |  |  |  |  |  |  |  |  |  |  |  |  |  |  | + |  |
| *panD* |  |  |  |  |  |  |  |  |  |  |  |  |  |  | + |  |
| *pchD* |  |  |  |  |  |  |  |  |  |  |  |  |  |  | + |  |
| *plr/gapA* | + |  |  |  |  |  |  |  |  |  |  |  |  |  | + |  |
| *Polysaccharide capsule(Bacillus)* |  |  |  |  |  |  |  |  |  | + | + | + |  | + | + |  |
| *sak* |  | + |  |  |  |  |  |  |  |  |  |  | + |  |  |  |
| *sdrC* |  | + |  |  |  |  | + |  | + |  |  | + |  | + |  |  |
| *sdrD* |  | + |  |  |  |  |  | + | + | + |  |  |  | + |  |  |
| *sdrE* |  | + | + |  | + |  | + | + | + | + |  | + |  | + |  |  |
| *sdrF* |  |  |  |  |  |  | + |  |  |  |  |  |  |  |  |  |
| *sdrG* |  |  |  |  |  |  | + |  |  |  |  |  |  |  |  |  |
| *sdrH* |  |  |  |  |  |  | + |  |  |  |  |  |  |  |  |  |
| *seb* |  | + |  |  |  |  |  |  |  |  |  |  |  |  |  |  |
| *sec* |  | + |  |  |  |  |  |  |  |  |  |  |  |  |  |  |
| *seg* |  | + |  |  |  |  |  |  |  |  |  |  |  |  |  |  |
| *sei* |  | + |  |  |  |  |  |  |  |  |  |  |  |  |  |  |
| *sell* |  | + |  |  |  |  |  |  |  |  |  |  |  |  |  |  |
| *selm* |  | + |  |  |  |  |  |  |  |  |  |  |  |  |  |  |
| *seln* |  | + |  |  |  |  |  |  |  |  |  |  |  |  |  |  |
| *selo* |  | + |  |  |  |  |  |  |  |  |  |  |  |  |  |  |
| *selp* |  | + |  |  |  |  |  |  |  |  |  |  |  |  |  |  |
| *selq* |  | + |  |  |  |  |  |  |  |  |  |  |  |  |  |  |
| *selu* |  | + |  |  |  |  |  |  |  |  |  |  |  |  |  |  |
| *Ser-Asp rich fibrinogen-binding proteins* |  |  |  |  |  |  |  |  |  |  |  |  |  | + |  |  |
| *set10* |  | + |  |  |  |  |  |  |  |  |  |  |  |  |  |  |
| *set11* |  | + |  |  |  |  |  |  |  |  |  |  |  |  |  |  |
| *set12* |  | + |  |  |  |  |  |  |  |  |  |  |  |  |  |  |
| *set13* |  | + |  |  |  |  |  |  |  |  |  |  |  |  |  |  |
| *set14* |  | + |  |  |  |  |  |  |  |  |  |  |  |  |  |  |
| *set15* |  | + | + |  |  |  |  |  |  |  |  |  |  |  |  |  |
| *set2* |  | + |  |  |  |  |  |  |  |  |  |  |  |  |  |  |
| *set26* |  | + |  |  |  | + |  |  |  | + |  |  |  |  |  |  |
| *set35* |  | + |  |  |  |  |  |  |  |  |  |  |  |  |  |  |
| *set6* |  | + |  |  |  |  |  |  |  |  |  |  |  |  |  |  |
| *set7* |  | + |  |  |  |  |  |  |  |  |  |  |  |  |  |  |
| *set8* |  | + |  |  |  |  |  |  |  |  |  |  |  |  |  |  |
| *set9* |  | + |  |  |  |  |  |  |  |  |  |  |  |  |  |  |
| *sigA/rpoV* | + |  |  |  |  |  |  |  |  |  |  |  |  |  |  |  |
| *slrA* | + |  |  |  |  |  |  |  |  |  |  |  |  |  |  |  |
| *spa* |  | + |  |  |  |  |  |  |  | + |  |  |  |  |  |  |
| *splA* |  | + |  |  |  |  |  |  |  |  |  |  |  |  |  |  |
| *splB* |  | + |  |  |  |  |  |  |  |  |  |  |  |  |  |  |
| *splC* |  | + |  |  |  |  |  |  |  |  |  |  |  |  |  |  |
| *splD* |  | + |  |  |  |  |  |  |  |  |  |  |  |  |  |  |
| *splE* |  | + |  |  |  |  |  |  |  |  |  |  |  |  |  |  |
| *splF* |  | + |  |  |  |  |  |  |  |  |  |  |  |  |  |  |
| *sspA* | + | + |  |  | + |  | + |  |  |  | + | + | + |  |  |  |
| *sspB* |  | + | + |  |  |  | + |  |  | + |  |  |  | + |  |  |
| *sspC* |  | + |  |  |  |  | + |  |  |  |  |  |  |  |  |  |
| *stp* |  |  |  |  |  |  |  |  |  |  |  |  |  |  | + |  |
| *sugC* |  |  |  |  |  |  |  |  |  |  |  |  | + |  | + | + |
| *T4SS effectors(Coxiella)* |  |  |  |  |  |  |  |  |  |  |  |  |  |  | + |  |
| *T6SS-II(Klebsiella)* | + |  |  |  |  |  |  |  |  |  |  |  |  |  |  |  |
| *tig/ropA* |  |  |  |  |  |  |  |  |  |  |  |  |  |  | + |  |
| *tsst* |  | + |  |  |  |  |  |  |  |  |  |  |  |  |  |  |
| *tuf* | + |  |  |  |  |  |  |  |  |  |  |  |  |  |  |  |
| *ugd* |  |  |  |  |  |  |  |  |  |  |  |  |  |  | + |  |
| *uge* | + |  |  |  | + |  |  |  | + | + |  |  |  | + |  | + |
| *undetermined_capsule* | + | + | + | + | + | + | + | + | + | + | + | + | + | + | + | + |
| *uppS* |  |  |  |  |  |  |  |  |  |  |  |  | + |  |  |  |
| *ureB* |  |  |  |  |  |  |  |  |  |  |  |  |  |  | + |  |
| *vctC* | + |  | + |  | + | + | + | + |  | + | + | + |  |  | + | + |
| *virR* |  |  |  |  |  |  |  |  |  |  |  |  |  |  |  | + |
| *wbtE* |  |  |  |  |  |  |  |  |  |  |  | + |  | + | + |  |
| *wbtF* |  |  |  |  |  |  |  |  |  |  |  |  |  |  | + |  |
| *wbtP* | + |  | + |  | + |  |  |  | + | + | + |  |  | + |  | + |
| *wcaJ* |  |  |  |  |  |  |  |  |  |  |  |  |  |  | + | + |
| *yent1* |  | + |  |  |  |  |  |  |  |  |  |  |  |  |  |  |
| *yent2* |  | + |  |  |  |  |  |  |  |  |  |  |  |  |  |  |

^*^**SA**: *S. aureus*; **SE**: *S. epidermidis*; **SAR***: S. arlettae*; **SAU**: *S. auricularis*; **SI**: *S. simulans*; **SSC**: *S. sciuri*; **SCH**: *S. chromogenes*; **SC**: *S. cohnii*; **SHY**: *S. hyicus*; **SS**: *S. saprophyticus*; **SAG**: *S. agnetis*; **SK**: *S. kloosii*; **SH**: *S. haemolyticus***; SF**: *S. felis*; **MC**: *Macrococcus caseolyticus*; **BS**: *Bacillus subtilis*.
